# Bridging the Gap between Charge Storage Site and Transportation Pathway in Molecular-Cage-Based Flexible Electrodes

**DOI:** 10.1021/acscentsci.3c00027

**Published:** 2023-04-05

**Authors:** Kang-Kai Liu, Zong-Jie Guan, Mengting Ke, Yu Fang

**Affiliations:** † State Key Laboratory for Chemo/Bio-Sensing and Chemometrics, College of Chemistry and Chemical Engineering, Hunan University, Changsha, Hunan 410082, People’s Republic of China; ‡ Innovation Institute of Industrial Design and Machine Intelligence Quanzhou-Hunan University, Quanzhou, Fujian 362801, People’s Republic of China

## Abstract

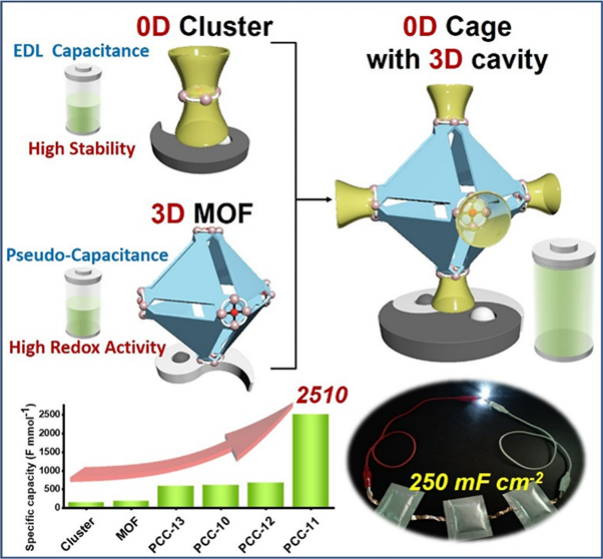

Porous materials have been widely applied for supercapacitors; however, the relationship between the electrochemical behaviors and the spatial structures has rarely been discussed before. Herein, we report a series of porous coordination cage (PCC) flexible supercapacitors with tunable three-dimensional (3D) cavities and redox centers. PCCs exhibit excellent capacitor performances with a superior molecular capacitance of 2510 F mmol^–1^, high areal capacitances of 250 mF cm^–2^, and unique cycle stability. The electrochemical behavior of PCCs is dictated by the size, type, and open–close state of the cavities. Both the charge binding site and the charge transportation pathway are unambiguously elucidated for PCC supercapacitors. These findings provide central theoretical support for the “structure–property relationship” for designing powerful electrode materials for flexible energy storage devices.

## Abstract 2

Developing powerful supercapacitors is essential for energy storage. We demonstrated the first example of a molecular cage supercapacitor that shows excellent performance and a controllable electron storage mechanism.

## Introduction

The growing trend for wearable electronics demands the evolution of flexible, high-performance, and durable energy storage systems.^1−4^ Supercapacitors with high capacitance and long cycle life have drawn great attention by taking advantage of tunable energy density and rate property.^5−10^ Typically, the electrodes with only electrical double-layer capacitors (EDLCs) exhibited excellent conductivity and cycling stability but usually showed intrinsic low capacitance.^11−14^ In contrast, pseudocapacitor-dominated electrodes have much improved capacitance while suffering from poor conductivity and low cycling stability. Therefore, a novel electrode material that combines both the advantages of EDLCs and pseudocapacitors is an essential approach to developing flexible high-performance supercapacitors.^15−17^

Conjugated porous polymers, such as metal–organic frameworks (MOFs), covalent–organic frameworks (COFs), and other conductive porous materials, have emerged as a new set of electrode materials for electrochemical energy storage applications because of the high surface area, the redox centers, and structural diversity.^18−26^ However, those materials frequently face several intrinsic problems, such as low electrochemical stability, low mechanical processability, and the lack of a “structure–property relationship”.^27−31^ Great efforts have been made to understand the working mechanism and reinforce the energy storage performance of MOF- or COF-based electrodes. Recently, Cheng et al. reported a group of MOFs and applied element and coordination number analysis to understand the lithium storage mechanism.^32^ The Zhang group elucidated that a COF underwent a proton-coupled electron transfer reaction for energy storage.^33^ Due to the lack of information on the charge storage site and charge transportation pathway in 3D porous networks, the energy storage mechanismd of the currently reported MOF- or COF-based electrodes are difficult to understand. Thus, a new flexible material with a 3D cavity/channel that can enhance the cycling stability and an investigation of the electron storage mechanism is highly demanded.

**Scheme 1:**
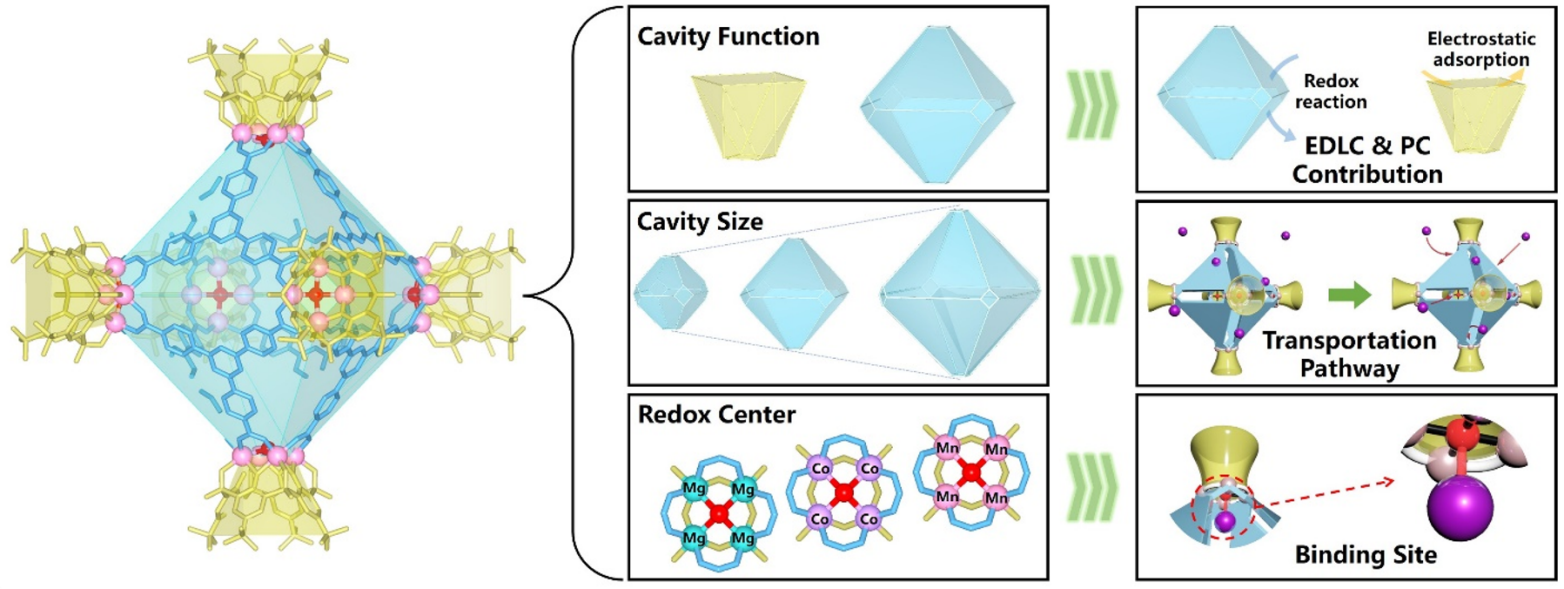
Different Components of PCCs Display Different Functions

As a small sibling of the MOF, the porous coordination cage (PCC) is a kind of discrete metal–organic assembly (diameter: 1–10 nm) with one or multiple 3D cavities, as well as tunable redox metal centers.^34−41^ PCCs inherited all the merits of MOFs and COFs by showing high porosity and a tunable redox center cavity (Scheme 1).^42−44^ These features of PCCs make them potential candidates for efficient energy storage materials; however, there have been no records about using them in supercapacitors.

Herein, we prepare a series of PCCs with different cavities and investigate their electrochemical behavior and energy storage mechanism as flexible supercapacitors. To our surprise, these PCCs exhibit excellent capacitor performances, especially for the Mn-based PCC, which has a superior molecular capacitance of up to 2514 F mmol^–1^, a high areal capacitance of 250 mF cm^–2^, and unique cycle stability. In addition, both the charge binding site and the transportation pathway are well illustrated for PCC-based supercapacitors by control experiments and theoretical calculations.

## Results and Discussion

### Synthesis and Structures of PCCs

**Figure 1:**
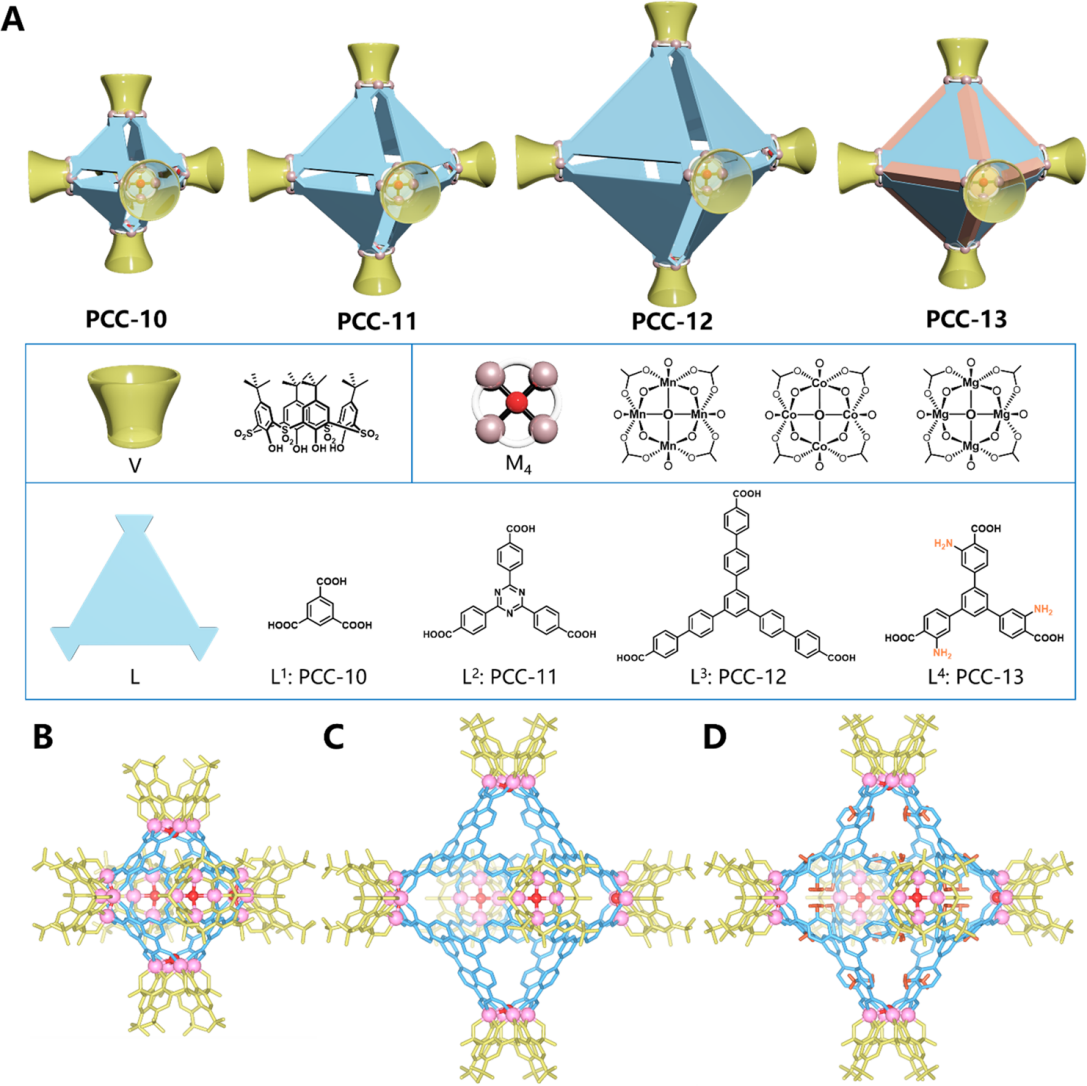
Structure details of PCCs. (A) Cartoon showing the structures of **PCC-10**, **PCC-11**, **PCC-12**, **PCC-13**, and their components. (B) SC-XRD structure of **PCC-10**. (C) SC-XRD structure of **PCC-11**. (D) SC-XRD structure of **PCC-13**.

Herein, we focused on a series of octahedral PCCs, containing a triangle panel ligand (H_3_L), vertex ligand (H_4_V), and metal cluster (M_4_O). First, we synthesized four PCCs, defined as **PCC-10**, **-11**, **-12**, and **-13**, with the same topologies and vertex ligands, as well as similar cavities and metal clusters (Figure 1A). For **PCC-10**, the panel ligand H_3_L^1^ is the smallest among the four H_3_L, thus making the inner cavity and the aperture the smallest among the four PCCs. When the panel ligand H_3_L^1^ was replaced by H_3_L^2^, **PCC-11** with a medium inner cavity was obtained. On further enlarging the panel ligand by using H_3_L^3^, **PCC-12** with the largest cavity could be formed. **PCC-13** was constructed by amino-functionalized H_3_L^2^ (i.e., H_3_L^4^), which adopted the same inner cavity in size as **PCC-11**; however, the cavity aperture is blocked by the functional groups. Besides, the M_4_-O cluster can be switched from redox-inert Mg(II) to redox-active Co(II) or Mn(II), without changing the cage topology (Tables S1–S7). Thus, **PCC-10**, **-11**, **-12**, and **-13** possess plentiful structures for investigating their energy storage applications.

The PCCs were synthesized by a solvothermal method (Section 1 in the Supporting Information). Single-crystal X-ray diffraction (SC-XRD) analyses indicate that all of the PCCs crystallized in the central-symmetric tetragonal space group *I*4/*m* (Figure 1 and Figures S1–S3). According to the crystal structures of the PCCs, the longest inner-cavity distances are 15.0, 25.1, and 35.2 Å for **PCC-10-Mn**, **PCC-11-Mn**, and **PCC-12-Co**, respectively (Figure 1B–D and Figure S5). We can ascertain the pore structure to testify to the intrinsic porosity of PCCs by a packing view and the nitrogen gas adsorption (Figures S1–S4 and S12–S14 and Table S8). FT-IR and UV spectra proved the successful preparation of **PCC-10**, **-11**, **-12**, and **-13** (Figures S6–S9).

### Electrochemical Characterizations

**Figure 2:**
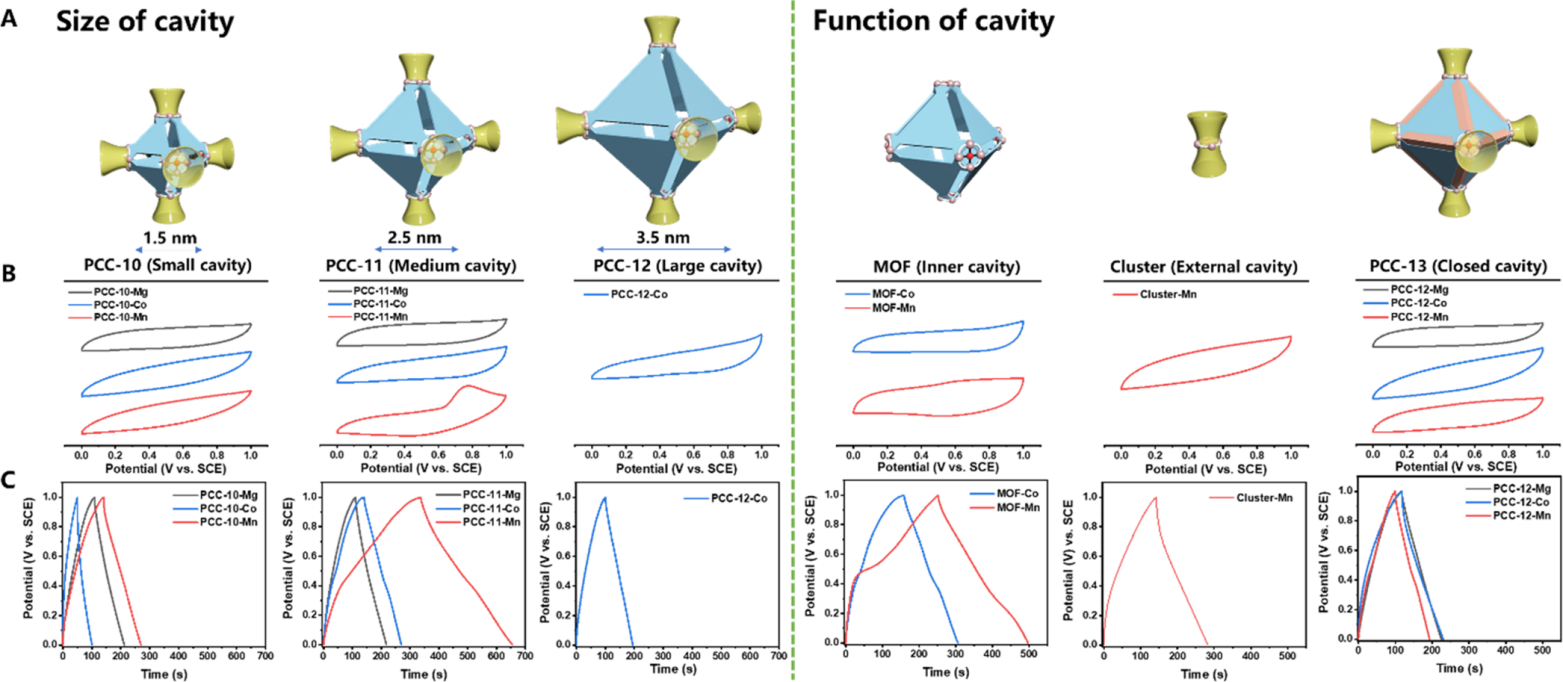
Electrochemical characterizations for PCC, MOF, and Cluster. (A) Schematic structures of **PCC-10**, **PCC-11**, **PCC-12**, MOF, Cluster, and **PCC-13**. (B) CV curves of PCC, MOF, and Cluster at 100 mV s^–1^. (C) Galvanostatic charge–discharge curves of PCC, MOF, and Cluster at 0.5 A g^–1^.

The electrochemical performances of PCCs were evaluated through a three-electrode system in a neutral aqueous electrolyte (3 M KCl) (for details see Section 4 in the Supporting Information). Initially, **PCC-10**, **-11**, and **-12** were chosen for investigating how the cavity size affects the electrochemical properties (Figure 2A). The electrodes of three **PCC-10-M** (M = Mg, Co, and Mn) with a small cavity (1.5 nm inner diameter) display rectangular CV curves, indicating that they only possess EDLC properties (Figure 2B). The highly symmetrical triangular GCD curves featured electrical energy storage physically based on an electrostatic interaction (Figure 2C). A maximum gravimetric capacitance of 75 F g^–1^ was attained for **the PCC-10-Mn** electrode in the **PCC-10** series, which exhibited moderate performance among the previously reported coordination materials. Interestingly, on enlarging the cavity size of the cage, the gravimetric capacitance was enhanced with the appearance of pseudocapacitive behavior. **PCC-11-Mg** exhibited a capacitance value of 40 F g^–1^ and a fully EDLC behavior, which are also consistent with the invariable valence of Mg (Figure 2B). **PCC-11-Co** exhibited an unsymmetrical right triangle CV curve, which is unique to Co complexes. The EDLC-based capacitance of **PCC-11-Co** provided a gravimetric capacitance of 78 F g^–1^. In stark contrast to the first two electrodes, **PCC-11-Mn** showed a reversible redox peak, verifying its pseudocapacitive properties. Owing to its discrete nature and ascertainable molecular weight, **PCC-11-Mn** reaches a record-high specific molecular capacitance, 1869.42 F mmol^–1^. By further enlarging the panel ligand, **PCC-12-Co** with a large cavity showed characteristics of the CV curve similar to those of **PCC-11-Co** with middle cavities; however, its gravimetric specific capacitance was reduced to 55 F g^–1^ (Figure 2B). This is probably because when the cavity size was enlarged, the percentage of metal center decreased, which mainly contributed to the pseudocapacitance. In addition, the specific molecular capacitance of **PCC-12-Co** was just 670 F mmol^–1^, even though the molecular weight increased significantly (Figure S20 and Table S9). As a result, when the cavity size was less than 2.5 nm, PCCs showed only EDLC regardless of the redox property of the metal centers. When the cavity size is larger than 2.5 nm, PCCs can exhibit pseudocapacitive properties according to the metal species, with the same metal center showing similar redox characteristics.

Since the PCC contains two cavities, an inner cavity (surrounded by eight-panel ligands) and six external cavities (defined by a single vertex ligand), we were curious about their roles in the electrochemical behavior. To determine the contribution of the two types of cavities, isostructural MOFs (**MOF-Mn** and **MOF-Co**, Figure S10)^45,46^ with only an inner cavity and an isostructural cluster (**Cluster-Mn**, Figure S11)^47^ bearing merely the external cavity were prepared, respectively (Figure 2A and Figure S4). In addition, to confirm whether the electrochemical behavior surely takes place inside the cavity, we designed **PCC-13** with a sealed cavity, by modifying −NH_2_ groups on the panel ligand that closed the apertures of the octahedral cage (Figure 2A).

As shown by the CV curves (Figure 2B), **MOF-Co** exhibited electrochemical characteristics similar to those of **PCC-11-Co**. Moreover, **MOF-Co** possessed a capacitance of 91 F g^–1^ comparable to that of **PCC-11-Co** (78 F g^–1^). Likewise, **MOF-Mn** showed redox peaks similar to those of **PCC-11-Mn**, exhibiting a capacitance of 130 F g^–1^. The observation of similar redox peaks from the CV spectra of both **PCC-11-M** (M = Co and Mn) and **MOF-M** (M = Co and Mn) indicated that the octahedral inner cavity of the PCC/MOF structure made a major contribution to pseudocapacitance. PCC exhibited much superior gravimetric capacitance (181 F g^–1^ vs 130 F g^–1^) and molecular capacitance (1873 F mmol^–1^ vs 182.61 F mmol^–1^) compared with MOFs with a similar topology and the same cavity.

Unexpectedly, **Cluster-Mn** did not exhibit the redox characteristic peak, even though it possesses the same tetranuclear Mn_4_ center as **MOF-Mn** and **PCC-11-Mn**. The gravimetric capacitance of **Cluster-Mn** only reached 75 F g^–1^, which is much lower than that of its two counterparts (Figure 2B). It is hypothesized that the hydrophobicity of the top *tert*-butyl on the vertex ligand could hinder the infiltration of the electrolyte, resulting in no effective contact between the charge carriers and the metal center. This stark contrast proved that the two cavities of PCC played different roles in specific capacitance: the inner cavity determined pseudocapacitance, while the external cavity governed EDLC. Given that there have only been a handful of discussions on the contribution of pseudocapacitance and EDLC in MOFs,^13^ this is the first report on quantitatively tuning the pseudocapacitance and EDLC for metal-complex-based electrodes under the same topology to obtain high-performance energy storage electrodes.

To further verify the necessity of the inner space in electrochemical energy storage, **PCC-13** with a closed cavity was introduced (Figure 2A). To our surprise, none of the **PCC-13** series exhibited the corresponding redox characteristics, and only EDLC was demonstrated (Figure 2B). The capacitances of **PCC-13-Mg**, **-Co,** and **-Mn** are 62, 64, and 55 F g^–1^ at 0.5 A g^–1^, respectively. Notably, the specific capacitance of **PCC-13-Mn** (55 F g^–1^) is slightly lower than that of **Cluster-Mn** (75 F g^–1^). The lack of pseudocapacitive behavior in the **PCC-13** series is probably due to the significant steric hindrance at the aperture of the inner cavity that arises from −NH_2_. Thus, the entry of charge carriers is significantly blocked, which suppresses the pseudocapacitance behavior.

**Figure 3:**
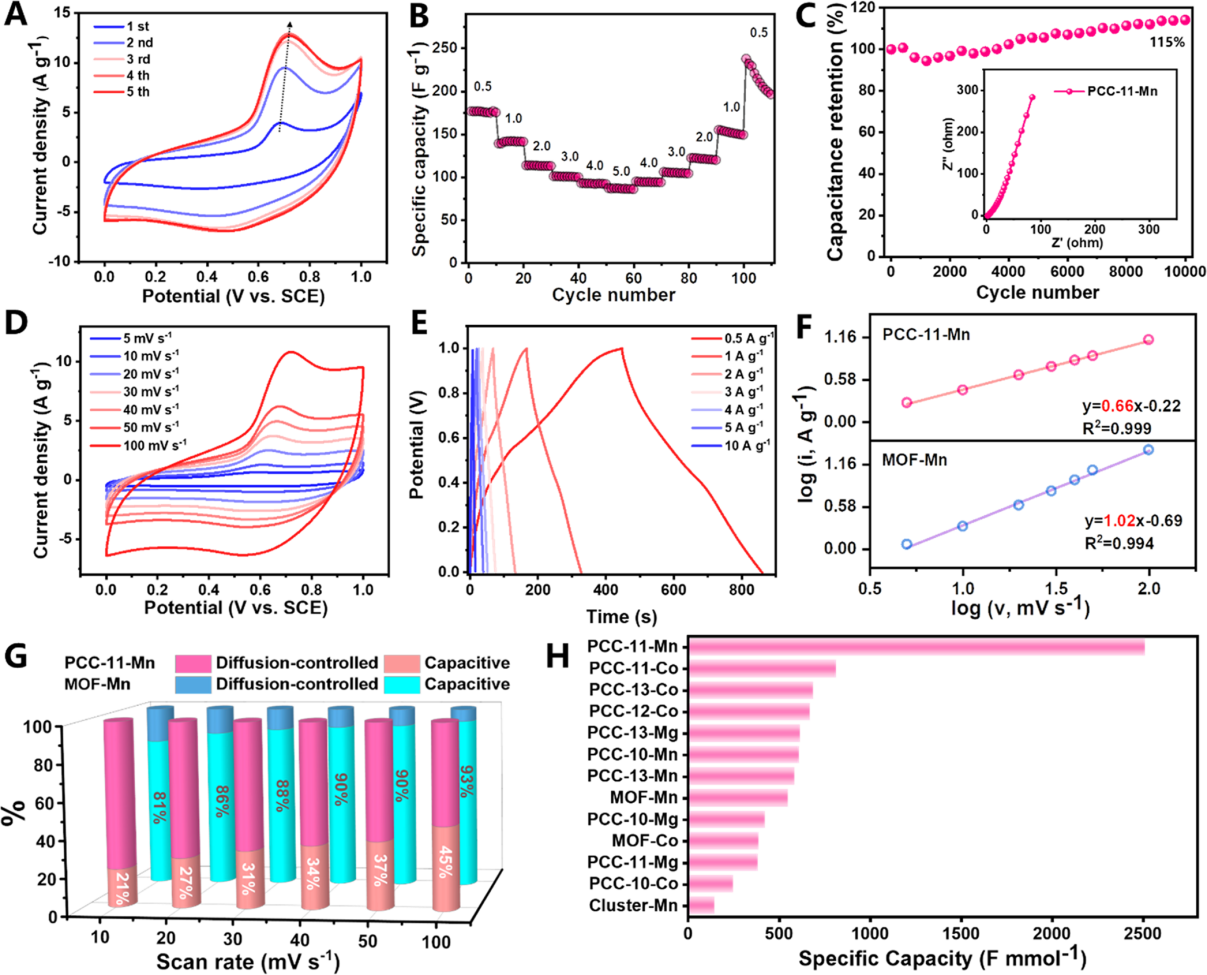
Electrochemical properties of **PCC-11-Mn**. (A) CV curves of **PCC-11-Mn** at 100 mV s^–1^. (B) Rate performance of **PCC-11-Mn**. (C) Cycle stability and EIS test of **PCC-11-Mn**. (D) CV curves of **PCC-11-Mn** after electrochemical activation. (E) GCD curves after electrochemical activation. (F) Linear fit of the peak current density for **PCC-11-Mn**. (G) Percentages of the capacitive and diffusion-controlled charge storage for **MOF-Mn** and **PCC-11-Mn**. (H) Specific capacities of all electrode materials.

Since the poor cycle stability of metal-complex-based supercapacitors hindered them from being used in practical applications,^7^ the performance of long-term reusability of PCC-based electrodes is considered to be a priority. Surprisingly, on examination of **PCC-11-Mn** at 100 mV s^–1^, CV curves exhibited a clear redox peak and gradually increased peak intensity in 5 cycles (Figure 3A). Unlike the swelling and shrinkage phenomenon commonly observed for other crystalline materials during the charge and discharge process,^5^**PCC-11-Mn** exhibited a rare activation behavior in the energy storage cycle, by showing a gradually increased CV curve area (Figure 3A). The rate performances of **PCC-11-Mn** were determined from 0.5 to 5 A g^–1^ for 120 cycling runs (Figure 3B). The specific capacitance was attenuated slightly in the first 60 runs and then was enhanced significantly in the following 60 runs, strongly indicating the activation behavior of PCC in the energy storage cycle. Thus, the retention rate of the specific capacitance of **PCC-11-Mn** was elevated to 115% with the staged growth after 10000 cycles (Figure 3C). Compared to MOF- or other framework-based electrodes^5^ bearing a maximum 99% initial retention capacitance after cycles, PCC underwent a gradual activation process during the charge–discharge cycle, yielding an unusual 15% increase in initial capacitance. To investigate this unusual activation behavior, contact angle and water vapor adsorption cycling tests were performed on **PCC-11-Mn** and **MOF-Mn** (Figures S27 and S28). The contact angles for pristine **PCC-11-Mn** and **MOF-Mn** are 107.26 and 98.63°, respectively, which proves that the MOF is more hydrophilic than the isostructural PCC. However, it is surprising that the contact angle of **PCC-11-Mn** was reduced to 76.82° after electrochemical activation, which is smaller than that of **MOF-Mn**. The much-decreased contact angle of the activated PCC suggested that the change from a hydrophobic to a hydrophilic nature may facilitate the penetration of water molecules and the electrolyte. Similarly, the water adsorption capacity of **PCC-11-Mn** (234 mg g^–1^) is slightly lower than that of **MOF-Mn** (276 mg g^–1^) in the first cycle. However, it increased significantly in the second (325 mg g^–1^) and third cycles (331 mg g^–1^), which surpassed those of the MOF counterpart (269 and 225 mg g^–1^). The increased water vapor adsorption further validated the gradual infiltration of the electrolyte, which facilitated the progressive activation of PCC in the aqueous electrolyte. These results validated that water molecules gradually penetrated the structural unit of discrete PCCs that facilitated the sustainable activation in electron storage. After full activation of **PCC-11-Mn** at 5 mV s^–1^ for 24 h, CV curves under scan rates ranging from 5 to 100 mV s^–1^ exhibited excellent electrochemical stability (Figure 3D). Meanwhile, the GCD curve (Figure 3E) shows a highly symmetrical charging and discharging process, presenting an approximately isosceles triangle with a small plateau. The gravimetric capacitance was calculated to be up to 243 F g^–1^ at 0.5 A g^–1^, which is a 34% enhancement compared to the pristine **PCC-11-Mn** (181 F g^–1^). This is among the best value for Mn-based supercapacitors in neutral aqueous electrodes.^48^ In short, a suitable cavity size, a quantitative combination of different cavity types, and a highly redox-active center will endow the PCC with the highest specific capacitance in the PCC series.

To comprehend the energy storage behavior of **PCC-11-Mn**, the capacitive contribution was compared with that of **MOF-Mn**. We can use the equation *i* = *av*^*b*^ (or log *i* = *b* log *v* + log *a*) to explore the connection between the peak current *i* in a CV curve and the scan rate *v*.^19,30^ The *b* values for **PCC-11-Mn** were close to 0.5 (0.66, Figure 3F), indicative of diffusion-controlled and battery-type reactions. The *b* values of **MOF-Mn** were close to 1 (1.02), indicating a pseudocapacitance-determined reaction. At 5 mV s^–1^, the capacitive contributiond to the total capacitance ertr 21% and 81% for **PCC-11-Mn** and **MOF-Mn**, respectively. As expected, because **MOF-Mn** only possesses an inner cavity, the total capacitance mainly comes from a capacitive contribution (93%). In contrast, since **PCC-11-Mn** has one inner cavity and six external cavities, the diffusion-controlled capacitance is roughly 1:1 (45%:55%). These results show that the significant high-speed capability of **PCC-11-Mn** comes from the strong capacitive effect at high scan rates, meanwhile verifying the claim that the ratio of inner cavity per unit reflected the percentage of pseudocapacitance. Furthermore, to investigate the molecular utilization of the electrodes during energy storage, we compared the molar-specific capacitance of all the materials in this work (Figure 3H). The postactivated **PCC-11-Mn** has a high capacitance value of 2509.77 F mmol^–1^, which is 13.8 times higher than that of **MOF-Mn** (182.61 F mmol^–1^) with the same inner cavity and 17.4 times higher than that of **Cluster-Mn** (144.43 F mmol^–1^) bearing an identical external cavity. It is observed that **MOF-M** (M = Mn, Co) with only an inner cavity and **Cluster-M** (M = Mn) with only an external cavity hardly catch up with the PCCs with both inner and external cavities in terms of molar-specific capacitance. Besides, **MOF-M** exhibited a gradual efficiency loss after cycles, while **Cluster-M** could maintain its performance (Figures S15–S19).

### Mechanism Study and DFT Calculations

**Figure 4:**
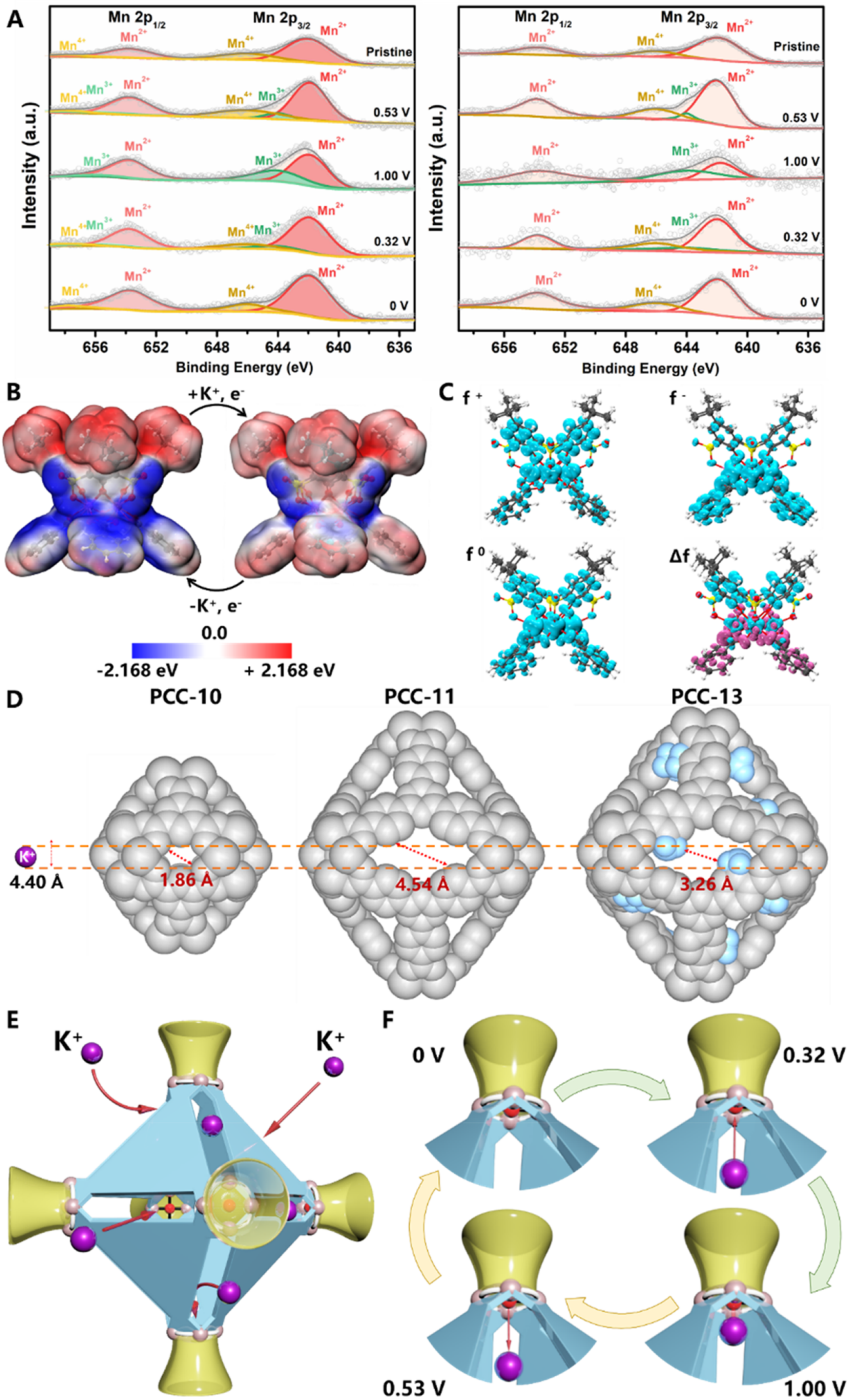
Mechanism study of the electrochemical process. (A) *Ex situ* XPS Mn 2p and O 1s spectra of **PCC-11-Mn** (left) and **MOF-Mn** (right) at different charged and discharged states. (B) Pseudocapacitive ESP images of **PCC-11-Mn** confirmed by DFT. (C) Graphical representation of the Fukui function for the **PCC-11-Mn** molecule. (D) Size of openings for **PCC-10**, **PCC-11**, **PCC-13**, and K^+^. (E) K^+^ pathway into the inner cavity of **PCC-11-Mn**. (F) The association–dissociation behavior of sodium cation to the electron storage center during the charging–discharging process.

To comprehend deeply the charge transportation pathway and binding site within the cage, *ex situ* X-ray photoelectron spectroscopy (XPS) analysis was applied. XPS results confirmed that the Mn and O elements in **PCC-11-Mn** underwent significant valence changes during the cycling process. The four peaks were assigned to Mn^2+^ at 642.03 and 653.93 eV and Mn^4+^ at 646.18 and 657.73 eV in two spin orbitals of Mn 2p_3/2_ and Mn 2p_1/2_ (Figure 4A).^49^ Although only Mn^2+^ was applied as the metal source in the formation of **PCC-11-Mn**, the newly appeared Mn^4+^ ion formed during the assembly indicated its potential for energy storage. In addition, the O 1s spectra were assigned to Mn–O (530.23 eV), S=O (531.18 eV), C–O (532.33 eV), and COO^–^ (533.28 eV), verifying the coordination to sulfone, phenolic hydroxyl groups, and the carboxylate ligand, respectively (Figure S21).^50−52^ When the electrode was charged to 0.53 V, new peaks at 656.18 and 644.28 eV were generated and could be assigned to Mn^3+^ 2p_3/2_ and Mn 2p_1/2_, respectively, verifying the onset of the pseudocapacitance process. On further charging to 1.00 V, the disappearance of peaks belonging to Mn^4+^ strongly indicated the significant transformation of Mn^4+^ to Mn^3+^. Meanwhile, a new peak at 529.58 eV in O 1s can be assigned to the K–O association. Thus, it is clear that the electronic charging facilitated the conversion of Mn^4+^ to Mn^3+^ in the tetranuclear Mn metal center, accompanied by a K^+^ ion coordinated to the μ_4_-O in the same metal center that compensated the charge balance. On discharging to 0.32 V, the signal of Mn^4+^ reappeared and the K–O peak at O 1s also decreased, which terminated the pseudocapacitance process. On further discharging to 0 V, Mn^3+^ was reversibly converted back to Mn^4+^ accompanied by the complete dissociation of K^+^ with the O. The “Mn^4+^–Mn^3+^ reversible interchange” accompanied by the association–dissociation of K^+^ with the μ_4_-O center facilitated the efficient energy storage of the PCC electrode and verified its excellent electrochemical stability. 1 has the same charging and discharging trend as 1 in Mn 2p_3/2_. However, the difference is that the Mn center with H_4_V (**PCC-10-Mn**, **PCC-13-Mn**, and **Mn-Cluster**) involved in the composition has a significant Mn^4+^ in Mn 2p_1/2_.

For comparison, **MOF-Mn** has the same redox trend behavior during the charging and discharging trend as **PCC-11-Mn** in Mn 2p_3/2_ (Figure 4A). It is worth noting that the Mn center with H_4_V (**PCC-10-Mn**, **PCC-13-Mn**, and **Mn-Cluster**) involved in the composition has a significant Mn^4+^ in Mn 2p_1/2_. However, no valence change of Mn was observed for **Cluster-Mn** (Figure S22), which accounts for its EDLC features. In addition, **PCC-10-Mn** and **PCC-13-Mn** both have the same valence distribution as **PCC-11-Mn** in their pristine Mn 2p spectra (Figures S23 and S24), but no valence changes during charging and discharging were observed due to their small and closed cavities, respectively. Coincidentally, **PCC-11-Co** and **PCC-11-Mg** show no valence change trends in the electrochemical cycle (Figures S25 and S26).

To further understand the charge docking site and kinetics of **PCC-11-Mn**, DFT calculations were carried out by Gaussian 16. In the calculations, an optimized structure based on the X-ray single-crystal structure was applied as a model (Figure 4B). The simulation results showed that K^+^ can only enter the cavity and approach the metal center of PCC during charging and coordinate with μ_4_-O, which is also strongly proved by the experimental data. In contrast, other O sites, such as carboxylic acid O, phenolic hydroxyl O, and sulfone O, cannot be used as the binding site for K^+^ according to the calculation results. The electrostatic potential (ESP) results displayed that the maximum positive ESP value of +2.168 eV was assigned to *tert*-butyl moieties in the vertex ligand of **PCC-11-Mn**, while the maximum negative point of −2.168 eV was assigned to the μ_4_-O group from the inner cavity.^53^ The high electronegativity of the μ_4_-O site is prone to nucleophilic reactions, which further verified the association with K^+^. A Fukui function analysis also confirmed that the conclusion matched well with ESP calculations (Figure 4C).^54−56^

Given the different capacitive behaviors induced by different cavity sizes, the opening sizes of several PCCs need to be measured for comparison with K^+^. **PCC-10** with a 0.5 nm cavity has an opening of only 1.86 Å (Figure 4D), which does not even allow H_2_O (4.00 Å) molecules to pass through. With an enlarged cavity, the **PCC-11** has an opening of 4.54 Å, which is sufficient to allow K^+^ (4.40 Å) and H_2_O to pass. However, **PCC-13** with the same inner cavity size (2.5 nm) as **PCC-11** is affected by the site resistance of modified −NH_2_. As a result, the opening is only 3.26 Å, which does not guarantee electrolyte entry into the cavity.

The energy storage process of **PCC-11-Mn** can be depicted through the analysis in Figure 4E,F. In the charging voltage range of 0–0.53 V, the energy storage mechanism of PCC is dominated by EDLCs. When the charging potential is over 0.53 V, the electronegative internal cavity starts to adsorb K^+^ from the electrolyte, while Mn^4+^ starts to convert to Mn^3+^. During the discharge process, a pseudocapacitive behavior occurs in the potential range from 1.0 to 0.32 V. Under this process, Mn^3+^ lost an e^–^ to generate Mn^4+^, while K^+^ gradually dissociated from the μ_4_-O site. As a result, the excellent performance of **PCC-11-Mn** can be ascribed to the gradual activation nature, reversible redox activity, and the tradeoff between EDLC and pseudocapacitance. We explore the charge storage pathways in the 3D space for the first time through a supramolecular coordination cage, demonstrating the rich designability of PCC structures and their potential electrochemical properties.

### Fabrication of PCC-Based Flexible Electrodes

**Figure 5:**
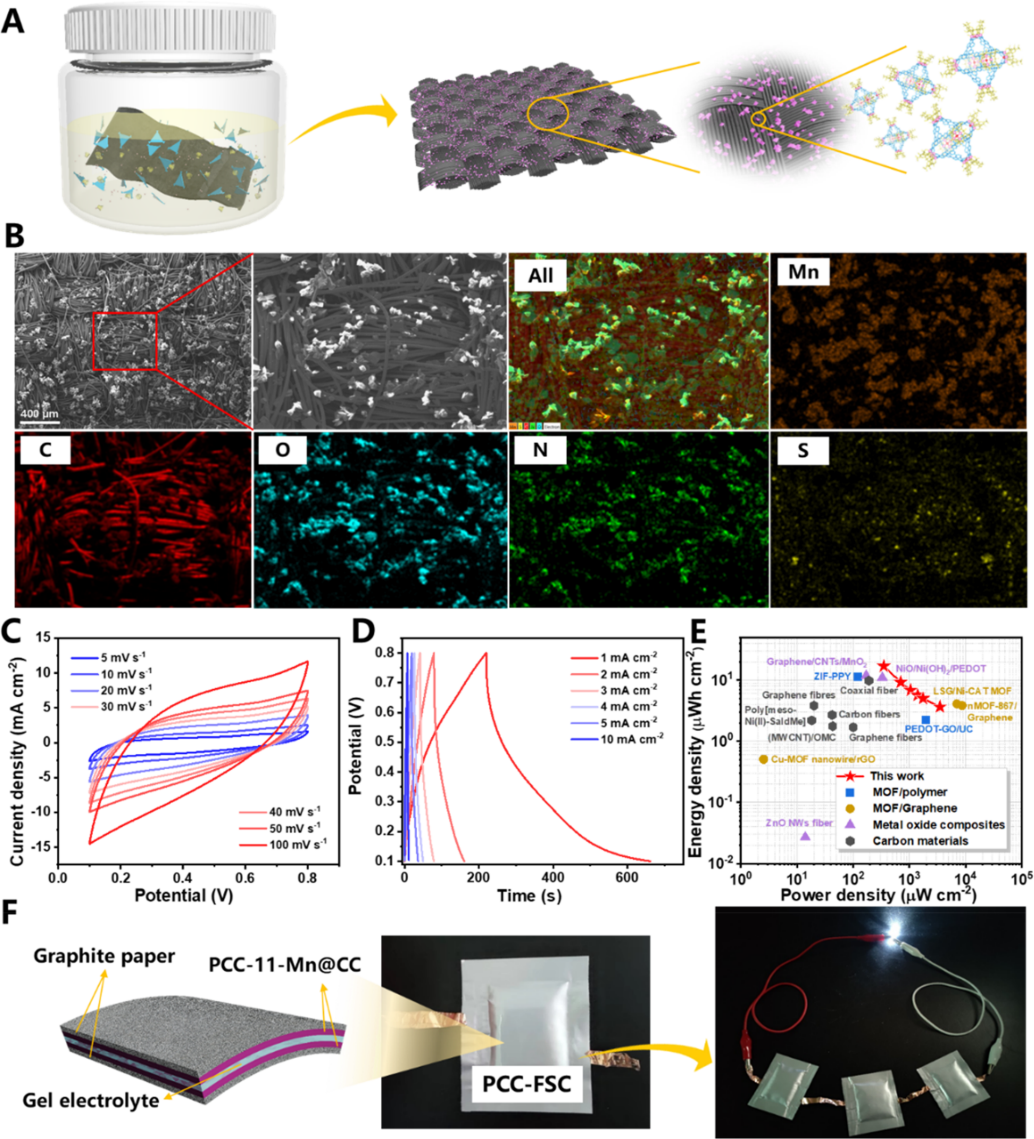
Fabrication and electrochemical property of **PCC-FSC**. (A) Schematic illustration of the in situ fabrication growth procedure for the **PCC-11-Mn@CC** electrode. (B) SEM and EDS mapping (all elements, Mn, C, O, N, S) images of **PCC-11-Mn@CC**. (C) CV curves of **PCC-FSC**. (D) GCD curves of **PCC-FSC**. (E) Ragone plots of **PCC-FSC** in comparison with other reported SCs. (F) Structure and application photos of **PCC-FSC**.

Unlike the poor mechanical property of framework materials, PCCs can be further immobilized on specific supports to build flexible electrodes with enhanced properties. Without a binder, **PCC-11-Mn** can be strongly immobilized in various current collectors, such as carbon cloth (CC), carbon paper (CP), Ni foam, and Cu foil, which facilitated its potential application as a flexible electrode. For example, by growing **PCC-11-Mn***in situ* on the surface of carbon cloth (CC) via a simple one-step method, a flexible **PCC-11-Mn@CC** electrode can be obtained (Figure 5A). Scanning electron microscopy (SEM) and energy dispersive X-ray spectroscopy (EDS) were applied to characterize the obtained electrodes (Figure 5B). The **PCC-11-Mn@CC** electrode exhibited extremely pronounced redox peaks at the same potentials (Figure S29a). The area capacitance was calculated to be up to 399 mF cm^–2^ at 0.5 mA cm^–2^, an exceptionally excellent result for crystalline materials with 3D topology (Figure S29b). This can be attributed to the good conductive matrix provided by CC, as also demonstrated by the Nyquist EIS (Figure S29c). Compared to PCC, the **PCC-11-Mn@CC** composite not only inherited the larger diffusion rate in the low-frequency region from the cage alone but also greatly reduced the charge transfer resistance (0.22 Ω) in the high-frequency region.

Finally, a PCC-based flexible symmetric all-solid-state supercapacitor **(PCC-FSC)** device was fabricated (Figure 5F) and the electronic property was measured (Figure 5C). The areal capacitance is calculated to be 250 mF cm^–2^ at 1 mA cm^–2^ (Figure 5D), which is much higher than those of most investigated devices (Table S10). **PCC-FSC** has excellent cycling stability, retaining 99% of its initial capacitance after 10000 cycles at 10 mA cm^–2^ (Figure S30). The bending experiment indicated excellent flexibility with no influence on the CV curves via bending (90°) or even folding (180°) the device (Figure S31). The Ragone plots (Figure 5E) correlating the areal energy densities and areal power densities demonstrate a maximum energy density of 17.04 μW cm^–2^ at a power density of 350 μWh cm^–2^. As the power density is increased by 1 order of magnitude, **PCC-FSC** retains a high energy density of 3.65 μW cm^–2^, which surpasses those of many types of porous-material-based devices (Figure 5E and Table S10).^15,57−69^ It was found that the PCC-based flexible device maintained the area capacitance and successfully lighted up a LED (Figure 5F). The results validated PCC as a potential flexible electrode to power small wearable electronic devices.

## Conclusions

In summary, a series of PCCs with tunable cavities and redox metal centers were prepared and fabricated into flexible electrodes. A high molecular capacitance of 2510 F mmol^–1^ at 0.5 A g^–1^ was achieved for **PCC-11-Mn**. Unlike previously reported crystalline electrodes, **PCC-11-Mn** exhibited a rare activation feature, resulting in an unprecedented 115% capacitance retention after 10000 cycles. This work is the first report on PCC-based supercapacitors, and the performance is among the best for 3D-framework-based electrodes. It was found that the inner cavity of the PCC dominated the pseudocapacitor, while the external cavity mainly contributed to the EDL capacitance. Moreover, the tailorable 3D cavity of PCC facilitated the investigation of both the charge storage site and transportation pathway at the same time. This not only proved the potential of PCC as an efficient energy storage device but also offered a new insight into designing supercapacitors as a way of trading off the EDL capacitance and pseudocapacitor contribution through the tuning of the spatial cavity.

## Supporting Information Available

Additional structural characterizations, including SC-XRD structures, PXRD patterns, FT-IR spectra, UV spectra, N_2_ adsorption–desorption isotherms, additional XPS spectra, additional SEM images, contact angle, water vapor adsorption isotherms, supported evidence for synthesis, electrochemical performance, and characterization of PCC and PCC-FSC (PDF). Structural information obtained by single crystal X-ray diffraction equipment (CIF files).
Crystallographic data (CIF)
checkCIF/PLATON report (PDF)
Crystallographic data (CIF)
checkCIF/PLATON report (PDF)
Crystallographic data (CIF)
checkCIF/PLATON report (PDF)
Crystallographic data (CIF)
checkCIF/PLATON report (PDF)
Crystallographic data (CIF)
checkCIF/PLATON report (PDF)
Crystallographic data (CIF)
checkCIF/PLATON report (PDF)
(Crystallographic data CIF)
checkCIF/PLATON report (PDF)

## Supplementary Material

oc3c00027_si_001.pdf

oc3c00027_si_002.cif

oc3c00027_si_003.pdf

oc3c00027_si_004.cif

oc3c00027_si_005.pdf

oc3c00027_si_006.cif

oc3c00027_si_007.pdf

oc3c00027_si_008.cif

oc3c00027_si_009.pdf

oc3c00027_si_010.cif

oc3c00027_si_011.pdf

oc3c00027_si_012.cif

oc3c00027_si_013.pdf

oc3c00027_si_014.cif
