# Supplementary material for: Bridging the Gap between Charge Storage Site and Transportation Pathway in Molecular-Cage-Based Flexible Electrodes
Source: ACS Cent Sci. 2023 Apr 5;9(4):805–15. doi: 10.1021/acscentsci.3c00027 (PMC10141610; doi:10.1021/acscentsci.3c00027)
Supplement: Supplementary file 1 — oc3c00027_si_001.pdf [file oc3c00027_si_001.pdf]

# Supporting Information

## Bridging the Gap between Charge Storage Site and Transportation Pathway in Molecular-Cage-Based Flexible Electrodes

*Kang-Kai Liu,* [1] *Zong-Jie Guan,* [1] *Mengting Ke,* [1] *and Yu Fang**[1][2]

[1] State Key Laboratory for Chemo/Bio-Sensing and Chemometrics, College of Chemistry and Chemical Engineering, Hunan University, Changsha, Hunan, 410082, China.

[2] Innovation Institute of Industrial Design and Machine Intelligence Quanzhou-Hunan University, Quanzhou 362801, Fujian, China.

*Corresponding Author(s): yu.fang@hnu.edu.cn.

# Table of Content


Section 1. Synthesis procedures and characterization methods................................3

Materials ........................................................................................................ 3

Characterization methods.................................................................................. 3

Calculation methods......................................................................................... 3

Theoretical Calculation ..................................................................................... 4

Synthesis of ligand ($H_3L$ ad $H_4V$)......................................................................... 4

Synthesis of PCC ............................................................................................. 6

Synthesis of Cluster .......................................................................................... 8

Synthesis of MOF ............................................................................................. 8

Synthesis of PCC-11-Mn@CC .......................................................................... 9

Section 2. Single-crystal X-ray structure of PCCs. ...............................................10

Section 3. Characterization of properties for the material. .....................................30

Section 4. $N_2$ adsorption-desorption measurements of PCCs................................33

Section 5. Electrochemical analysis of PCCs, MOFs, and Clusters. ......................37

Section 6. Summary and comparison of performance. ...........................................41

Section 7. XPS spectra of PCCs, MOFs, and Clusters. ..........................................44

Section 8.  Mechanism of charging and discharging process. ................................50

Section 9. Flexible electrode of PCC-11-Mn.........................................................53

Section 10. Comparison of electrochemical performance. .....................................56

## Section 1. Synthesis procedures and characterization methods

## Materials

Trimesic acid (99%, $H_3L^1$, Adamas), 1,3,5-Tri(4-carboxyphenyl)benzene (99%, $H_3BTB$, Leyan), 1，3，5-Tris(4'-carboxy[1，1'-biphenyl]-4-yl)benzene (99%, $H_3L^3$, Leyan) were used as obtained. All commercial chemicals were used without further purification.

## Characterization methods

The single-crystal X-ray structure was obtained on a Bruker "Quest" diffractometer equipped with a MoKα sealed-tube X-ray source (graphite radiation monochromator, λ = 0.71073) and a low-temperature device (110 K). The Brunauer-Emmett-Teller (BET) surface area and pore size distribution data were collected by $N_2$ adsorption/desorption tests at 77 K using BSD-660 (Beishide. Co. China). PXRD patterns were obtained by powder X-Ray diffractometer (D8 ADVANCE). Water vapor adsorption tests were performed by Vacuum & Dynamic Vapor/Gas Sorption Analyzer (BSD-VVS&DVS, Beishide. Co. China). FT-IR and UV spectra were obtained by IR-Spirit-T (206-31010-58) and UV-1900i from SHIMAZU. X-ray photoelectron spectroscopy (XPS) data were collected by a Thermo Scientific K-Alpha. SEM images and EDX mapping were taken with a Jeol JSM-7610FPlus scanning electron microscopy and an Oxford ULTIM MAX 40 Energy Dispersive Spectroscopy. Cyclic voltammetry (CV), galvanostatic charge-discharge (GCD), electrochemical impedance spectroscopy (EIS) measurements, and cyclic stability were carried out on a CHI660E electrochemical station (Shanghai Chenghua, China). All the electrochemical measurements were carried out at 25 ℃.

## Calculation methods

Gravimetric capacitances in three electrodes are calculated from GCD profiles according to the equation:

$$C = \frac{I_m \times \Delta t}{\Delta U}$$

Where $C$ (F $g^{-1}$) is the gravimetric capacitance, $I_m$ (A $g^{-1}$) stands for the current density, $\Delta t$ (s) is the discharge time, and $\Delta U$ (V) is the potential window interval.

The formula to calculate the areal capacity ($C_S$) is written as:

$$C_S = \frac{C \times m}{S}$$

$S$ ($cm^2$) is the working area of the electrode. The active mass of a single electrode is 0.7 mg, and the loadings mass of the PCC-11-Mn electrode is controlled to be 1 mg $cm^{-2}$.

The gravimetric energy density ($E$) and power density ($P$) are calculated according to the following two equations:

$$E(\text{Wh kg}^{-1}) = \frac{0.5 \times C \times \Delta U^2}{3.6}$$

$$P(\text{kW kg}^{-1}) = \frac{3.6 \times E}{\Delta t}$$

Where $E$ (W h $kg^{-1}$) is the gravimetric energy density, $P$ (kW $kg^{-1}$) is the gravimetric power density of the symmetrical supercapacitor system, $C$ is the gravimetric capacitances of the device, $\Delta U$ is the voltage window, $\Delta t$ is the discharge time.

## Theoretical Calculation

Quantum chemical studies are performed using density functional theory (DFT) implemented in GAUSSIAN 16 package. Geometry optimization and frequency analysis are calculated at B3LYP hybrid functional [Ref.1] with 6-31G(d) basis sets. Fukui function indices [Ref.2] and Electrostatic potential surfaces (ESP) are analyzed by Multiwfn 3.8 [Ref.3] and VMD v 1.9.3 molecular visualization software [Ref.4].

## Synthesis of ligand ($H_3L$ ad $H_4V$)

4, 4', 4''-s-triazine-2, 4, 6-triyltribenzoic acid ($H_3L^2$) was synthesized as described in the literature [Ref. 5]. Briefly, p-tolunitrile (1.45 mL, 12.15 mmol) was added slowly to trifluoromethane sulfonic acid (5 mL). The mixture was stirred overnight, then the reaction mixture was poured on crushed ice followed by neutralization with aqueous ammonia which gave white color precipitate.

The precipitate was collected by filtration and washed with water and acetone. 2, 4, 6-tri-p-tolyl-s-triazine was recrystallized from toluene which yielded 1.4 g (98 %). Then, 2, 4, 6-tri-p-tolyl-s-triazine (1.0 g, 2.8 mmol), acetic acid (24.85 mL), and sulfuric acid (1.58 mL) were taken in a 100 mL flask and stirred for 5 min. After heating the mixture to 50 ℃, chromium trioxide (2.58 g) dissolved in a mixture of acetic acid (5 mL) and sulfuric acid (5 mL) was added slowly. The dark brown slurry was stirred overnight followed by the filtration of the reaction mixture. The residue was collected and re-dissolved in 2 M NaOH and filtered to remove any insoluble impurities. $H_3L^2$ was obtained by acidifying the solution with 6 M HCl.

*p-tert*-Butylsulfonylcalix[4]arene ($H_4V$) was synthesized as described in the literature [Ref. 6]. p-tert-butylphenol (64.5 g, 0.43 mol), sulfur $S_8$ (27.5 g, 0.86 mol), and NaOH (8.86 g, 0.215 mol) was stirred in 35 mL tetraethylene glycol dimethyl ether under nitrogen. The stirred mixture was heated gradually to 230 ℃ for 4 h and kept at this temperature for a further 3 h with concomitant removal of the evolving hydrogen sulfide with a slow stream of nitrogen. The resulting dark red product was cooled to ambient temperature and diluted with toluene (35 mL) and 4 M aq. Sulfuric acid solution (4 M, 140 mL), followed by the addition of diethyl ether (140 mL) with stirring to give a suspension. The precipitate (TC4A) was collected by filtration, recrystallized from chloroform, and dried in vacuo. Then, a solution of TC4A (1.0 g, 1.38 mmol) in chloroform (30 mL) was added to acetic acid (50 mL) and $NaBO_3{\cdot}4H_2O$ (2.0 g, 13 mmol). After the mixture had been stirred at 50 ℃ for 18 h, $H_4V$ was extracted with chloroform and recrystallized from benzene-methanol, and dried in vacuo.

($H_3L^3$) was synthesized as described in the literature [Ref. 7]. A mixture of 4-acetyl-4'-bromobiphenyl (13.2 g, 48.0 mmol) and trifluoromethanesulfonic acid (2.4 mL) in toluene (100 mL) was heated to reflux under an argon atmosphere for 14 h. The formed precipitate was filtered off, washed with methanol, and re-crystallized from chloroform. To a solution of 1,3,5-tris(4′-bromobiphenyl-4-yl)benzene (2.01 g, 2.61 mmol) in dry THF (120 mL), n-butyllithium (6.3 mL, 2.5 mol L-1 solution in hexane) was added slowly at -70 ℃ under argon atmosphere. After stirring the solution for 6 h at 203 K, CO2 gas was passed through the reaction mixture for 30 min. After the solution was warmed up to room temperature under CO2 flow, water was added to remove the

excess of n-BuLi. Acidifying the solution with acetic acid gave a clear solution. The THF was removed under reduced pressure and the resulting white precipitate was collected by filtration.

Diethyl 3,3''-diamino-5'-(3-amino-4-(ethoxycarbonyl)phenyl)-[1,1':3',1''-terphenyl]-4,4''-dicarboxylate ($H_3L^4$) was synthesized as described in the literature [Ref. 9]. Anhydrous DMF (10 mL) was purged with $N_2$ and then transferred via a cannula into a three-neck round-bottomed flask charged with 1,3,5-tribromobenzene (1.00 g, 3.17 mmol) and bis(pinacolato)diboron (2.54 g, 9.53 mmol). Potassium acetate (1.87 g, 19.0 mmol) and Pd(dppf)$Cl_2$ (0.087 g, 0.12 mmol) were then quickly added into the flask. The resulting mixture was stirred vigorously and heated at 90 ℃ for 24 h. A mixture of resulted compound (0.79 g, 1.7 mmol) and methyl 2-amion-4-bromonbenzoate (1.35 g, 5.88 mmol) was dissolved in 48 mL mixed solvent of p-dioxane/$H_2O$ (1:1 v/v), which was deoxygenated by three freeze-pump-thaw cycles and protected under $N_2$ atmosphere. After quickly adding CsF (2.40 g, 15.7 mmol) and Pd(dppf)$Cl_2$ (0.095 g, 0.13 mmol), the suspension was heated and stirred vigorously at 90 ℃ for 24 h. After cooling down to room temperature, the resulting suspension was added with 150 mL of 20% $NH_4Cl$ solution and extracted three times with 3 × 50 mL EtOAc using a 250 mL separatory funnel. The organic layers were combined, washed with saturated brine, dried with anhydrous $Na_2SO_4$, and filtered. A crude product was obtained after removing all the solvents by rotary evaporation and further purified by quick chromatography using $CH_2Cl_2$/EtOAc (15:1 v/v) as eluent.

## Synthesis of PCC

**PCC-10-Mn**: $H_4V$ (84.9 mg, 0.1 mmol), $H_3L^1$ (21 mg, 0.1 mmol), and $MnCl_2$ (62.9 mg, 0.5 mmol) were suspended in 10 mL DMF with 5 mL MeOH. The mixture was heated at 100 ℃ in an oven for 24 h. After cooling to ambient temperature, large faint yellow crystals were collected and washed with methanol.

**PCC-10-Co**: The synthesis of PCC-10-Co using organic linker $H_4V$ and $H_3L^1$ was adapted from Wang (2012) named as MOSC-1-Co [Ref.8]. $H_4V$ (84.9 mg, 0.1 mmol), $H_3L^1$ (69.3 mg, 0.33 mmol), and $CoCl_2 \cdot 6H_2O$ (119.0 mg, 0.5 mmol) were suspended in 10 mL DMF. The mixture was

heated at 100 ℃ in an oven for 24 h. After cooling to ambient temperature, large purple crystals were collected and washed with methanol.

**PCC-10-Mg**: The synthesis of PCC-10-Mg using organic linker $H_4V$ and $H_3L^1$ was adapted from Wang (2012) and named as MOSC-1-Mg [Ref.8]. $H_4V$ (84.9 mg, 0.1 mmol), $H_3L^1$ (69.3 mg, 0.33 mmol), and $MgCl_2$ (47.6 mg, 0.5 mmol) were suspended in 10 mL DMF. The mixture was heated at 100 ℃ in an oven for 24 h. After cooling to ambient temperature, large colorless crystals were collected and washed with methanol.

**PCC-11-Mn**: $H_4V$ (8.5 mg, 0.01 mmol), $H_3L^2$ (13.4 mg, 0.033 mmol), and $MnCl_2$ (12.5 mg, 0.1 mmol) were suspended in 2 mL DMF with 1 mL MeOH. The mixture was heated at 100 ℃ in an oven for 24 h. After cooling to ambient temperature, large faint yellow crystals were collected and washed with methanol.

**PCC-11-Co**: $H_4V$ (8.5 mg, 0.01 mmol), $H_3L^2$ (13.4 mg, 0.033 mmol), and $CoCl_2{\cdot}6H_2O$ (23.8 mg, 0.1 mmol) were suspended in 2 mL DMF with 1 mL MeOH. The mixture was heated at 100 ℃ in an oven for 24 h. After cooling to ambient temperature, large purple crystals were collected and washed with methanol.

**PCC-11-Mg**: $H_4V$ (8.5 mg, 0.1 mmol), $H_3L^2$ (13.4 mg, 0.033 mmol), and $MgCl_2$ (9.5 mg, 0.1 mmol) were suspended in 2 mL DMF with 1 mL MeOH. The mixture was heated at 100 ℃ in an oven for 24 h. After cooling to ambient temperature, large colorless crystals were collected and washed with methanol.

**PCC-12-Co**: The synthesis of PCC-12-Co using organic linker $H_4V$ and $H_3L^3$ was adapted from Liao (2015) named as CIAC-114-Co [Ref.7]. $H_4V$ (43 mg, 0.05 mmol), $H_3L^4$ (30 mg, 0.05 mmol), and $CoCl_2{\cdot}6H_2O$ (50 mg, 0.2 mmol) were suspended in 3 mL DMA. The mixture was heated at 130 ℃ in an oven for 72 h. After cooling to ambient temperature, large purple crystals were collected and washed with methanol.

**PCC-13-Mn**: $H_4V$ (8.5 mg, 0.01 mmol), $H_3L^4$ (14 mg, 0.033 mmol), and $MnCl_2$ (12.5 mg, 0.1 mmol) were suspended in 1 mL DMF. The mixture was heated at 100 ℃ in an oven for 24 h. After cooling to ambient temperature, large faint yellow crystals were collected and washed with methanol.

**PCC-13-Co**: $H_4V$ (8.5 mg, 0.01 mmol), $H_3L^4$ (14 mg, 0.033 mmol), and $CoCl_2 \cdot 6H_2O$ (23.8 mg, 0.1 mmol) were suspended in 1 mL DMF. The mixture was heated at 100 ℃ in an oven for 24 h. After cooling to ambient temperature, large purple crystals were collected and washed with methanol.

**PCC-13-Mg**: $H_4V$ (8.5 mg, 0.01 mmol), $H_3L^4$ (14 mg, 0.033 mmol), and $MgCl_2$ (9.5 mg, 0.1 mmol) were suspended in 1 mL DMF with 2 drops of $Et_3N$. The mixture was heated at 100 ℃ in an oven for 24 h. After cooling to ambient temperature, large colorless crystals were collected and washed with methanol.

## Synthesis of Cluster

**Cluster-Mn**: Cluster-Mn was synthesized by the modified method [Ref.10]. $H_4V$ (17 mg, 0.02 mmol), and $MnCl_2$ (12.5 mg, 0.1 mmol) were suspended in 2 mL DMF with 2 drops of $Et_3N$. The mixture was heated at 100 ℃ in an oven for 24 h. After cooling to ambient temperature, large brown crystals were collected and washed with methanol.

## Synthesis of MOF

**MOF-Mn**: The synthesis of MOF-Mn using organic linker $H_3L^2$ was adapted from Zhou (2009) named PCN-9 (Mn) [Ref.11]. $H_3L^2$ (10 mg, 0.023 mmol), $Mn(ClO_4)_2 \cdot 6H_2O$ (15 mg, 0.052 mmol), and $Mn(CH_3COO)_3 \cdot 2H_2O$ (15 mg, 0.056 mmol) were suspended in 1.2 mL DMSO. The mixture was heated at 135 ℃ in an oven for 72 h. After cooling to ambient temperature, large faint yellow crystals were collected and washed with methanol.

**MOF-Co**: The synthesis of MOF-Co using organic linker $H_3L^2$ was adapted from Zhou (2009) named PCN-9 (Co) [Ref.11]. $H_3L^2$ (10 mg, 0.023 mmol), and $Co(NO_3)_2 \cdot 6H_2O$ (25 mg, 0.086 mmol) were suspended in 1.2 mL DMSO with 6 drops of DMF and 3 drops of $HBF_4$. The mixture was heated at 135 ℃ in an oven for 72 h. After cooling to ambient temperature, large purple crystals were collected and washed with methanol.

## Synthesis of PCC-11-Mn@CC

$H_4V$ (8.5 mg, 0.1 mmol), $H_3L^2$ (13.4 mg, 0.033 mmol), and $MnCl_2$ (12.5 mg, 0.1 mmol) were suspended in 2 mL DMF with 1 mL MeOH. The clean carbon cloth was immersed into the reaction solution and heated in an oven for 24 h. After cooling to ambient temperature, The PCC-11-Mn coated carbon cloth (PCC-11-Mn@CC) was collected and washed thoroughly with methanol 3 times, and then treated by vacuum drying.

## Section 2. Single-crystal X-ray structure of PCCs.

PCCs are octahedral supramolecular structures containing triangle panel ligand ($H_3L$), vertex ligand ($H_4V$), and metal cluster ($M_4O$). In each octahedron, six vertexes are composed of calix[4]arene ($H_4V$), and eight carboxylate ligands ($H_3L$) are placed at the eight faces. Tetranuclear $M_4$-$\mu_4$-O cluster (M=Mn, Co, and Mg) coordinated with carboxylate groups from $H_3L$ and phenolic moieties from $H_4V$, thus assembling a cage structure with an inner cavity and six external cavities. Because the panel ligand can be easily extended or functionalized, the diameter of the inner cavity can be finely adjusted. Besides, since the functional groups are located at the aperture of the cavity, they may block the entrance of the guest molecule, e. g. charge carrier, which will in turn affect the redox property of PCCs.

Single crystal X-ray diffraction (SC-XRD) analyses indicate that all the PCCs crystallized in the central-symmetric tetragonal space group *I*4/m (Figure 1, Figure S1~3, Table S1~7). As expected, the obtained PCCs consist of six tetranuclear metal clusters ($M_4$-$\mu_4$-O, M=Mn, Co, and Mg) as vertices and eight-panel ligands as faces ($L^1$, $L^2$, $L^3$, and $L^4$, respectively) assembled into an octahedron cage (Figure 1). Each metal atom in the $M_4$-$\mu_4$-O cluster is located at the four corners of the cluster square plane and held in a pseudo-octahedral configuration by coordination with six oxygen atoms. The six oxygen atoms include one sulfonic oxygen, two phenolate oxygen atoms from V, two carboxylate oxygens from two adjacent L, and a $\mu_4$-$OH_2$**.**

Crystallographic data for the reported crystal structures have been deposited at the Cambridge Crystallographic Data Centre via www.ccdc.cam.ac.uk with codes 2212168 (**PCC-10-Mn**), 2212171 (**PCC-11-Co**), 2212172 (**PCC-11-Mg**), 2212173 (**PCC-11-Mn**), 2212175 (**PCC-13-Co**), 2212178 (**PCC-13-Mg**), and 2212181 (**PCC-13-Mn**). The CIF files and checkcif files of PCCs were also attached in supporting information.

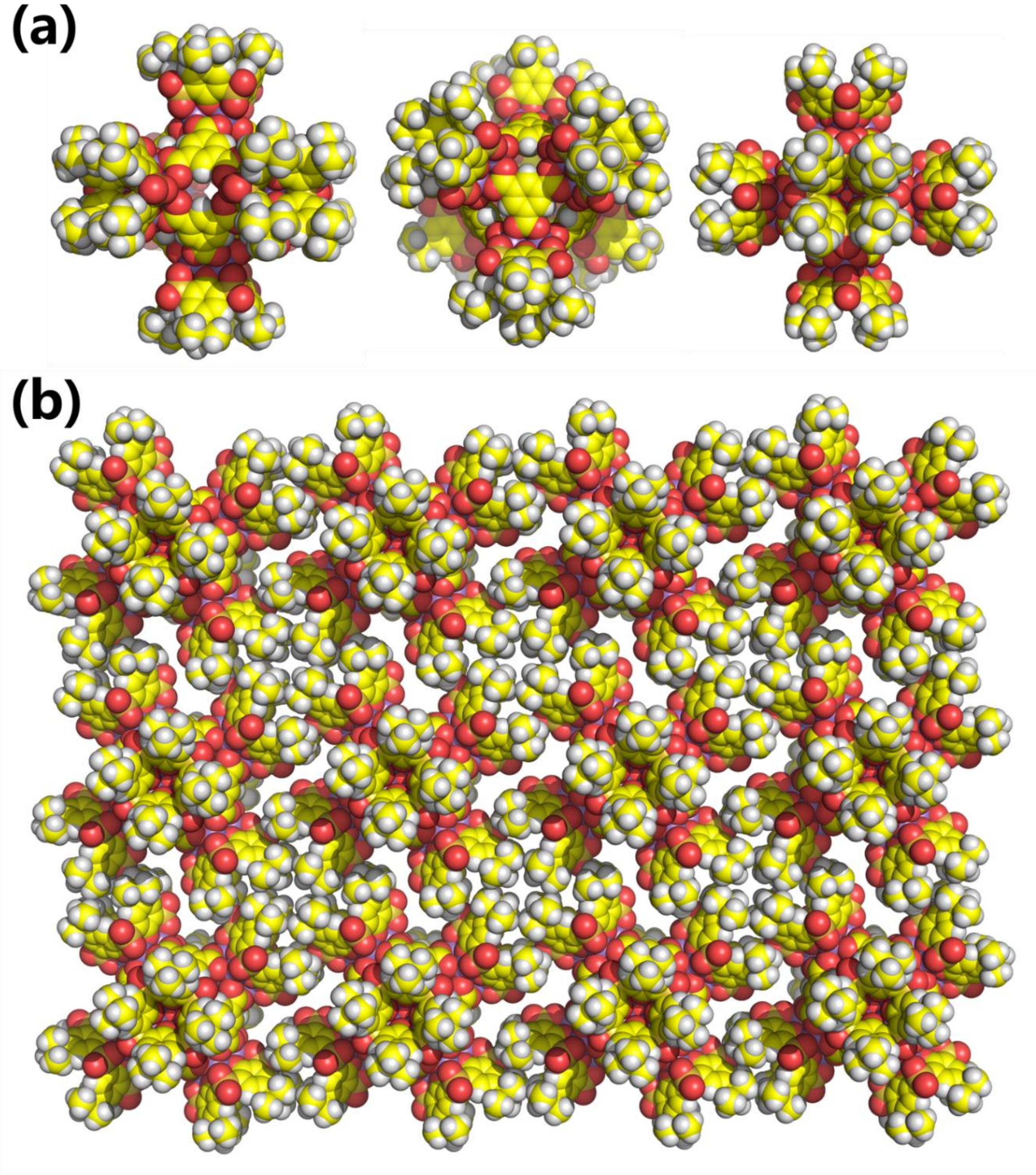


**Figure S1.** Different single view (a) and crystal packing view (b) of **PCC-10** as determined by SC-XRD.

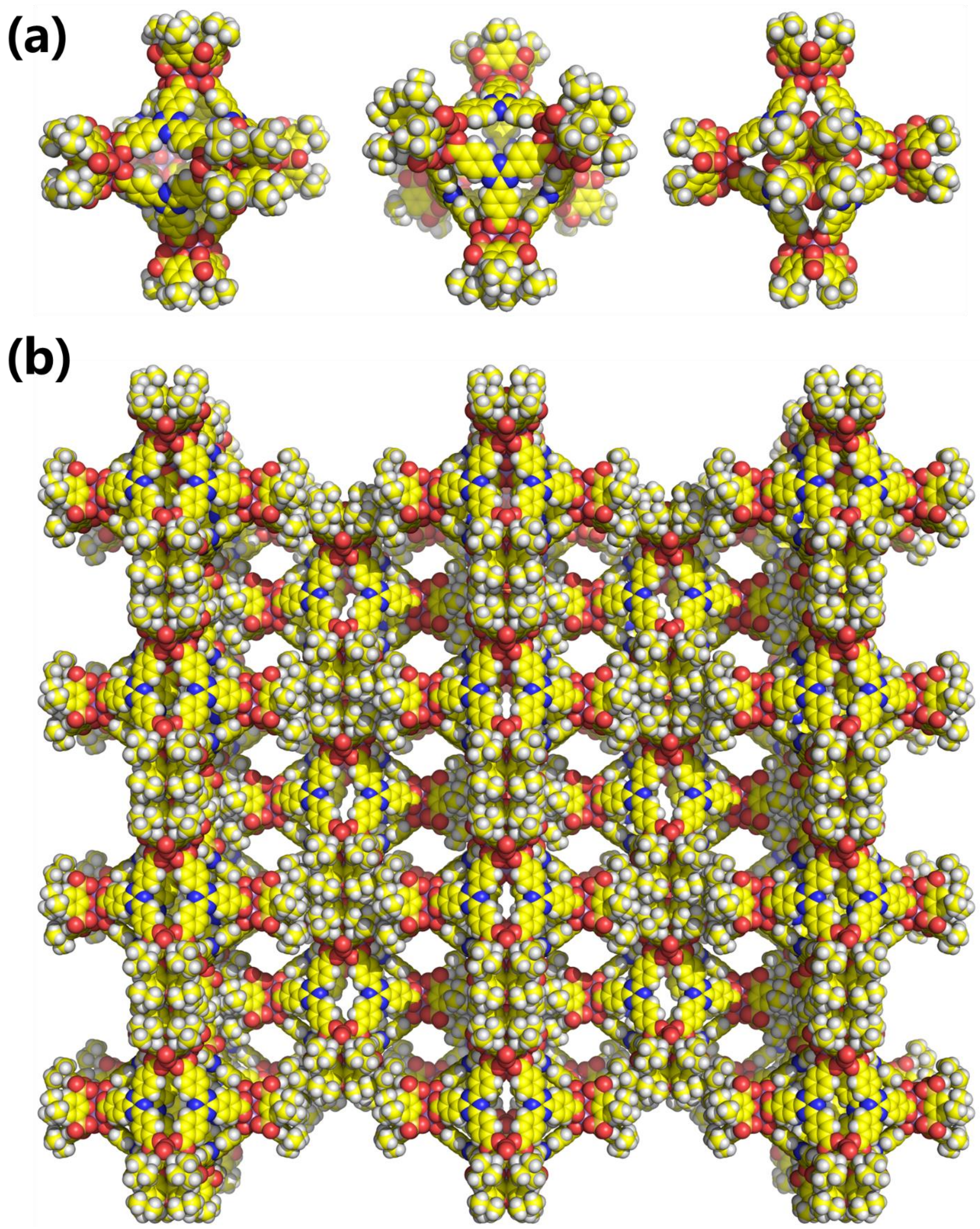


**Figure S2.** Different single view (a) and crystal packing view (b) of **PCC-11** as determined by SC-XRD.

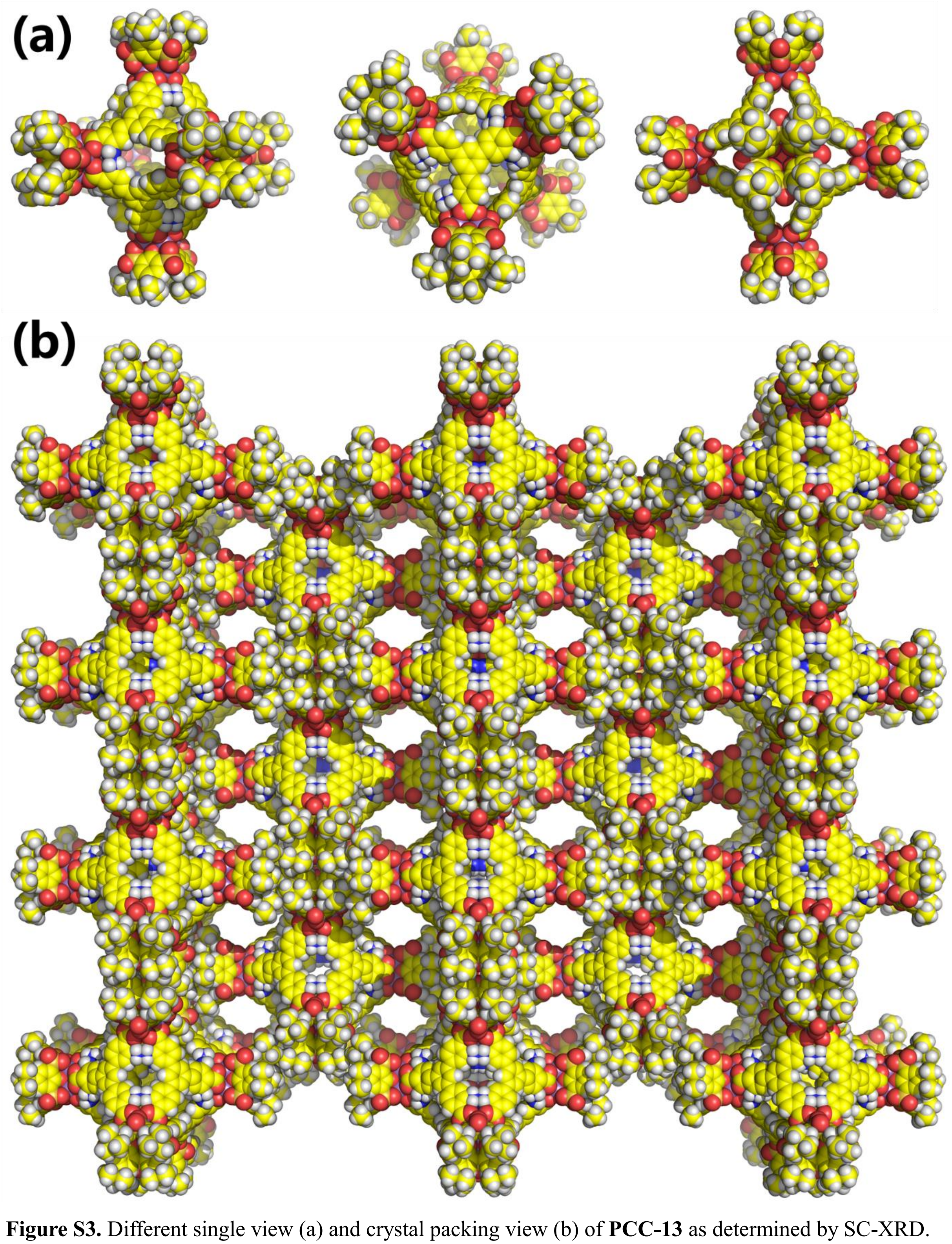


**Figure S3.** Different single view (a) and crystal packing view (b) of **PCC-13** as determined by SC-XRD.

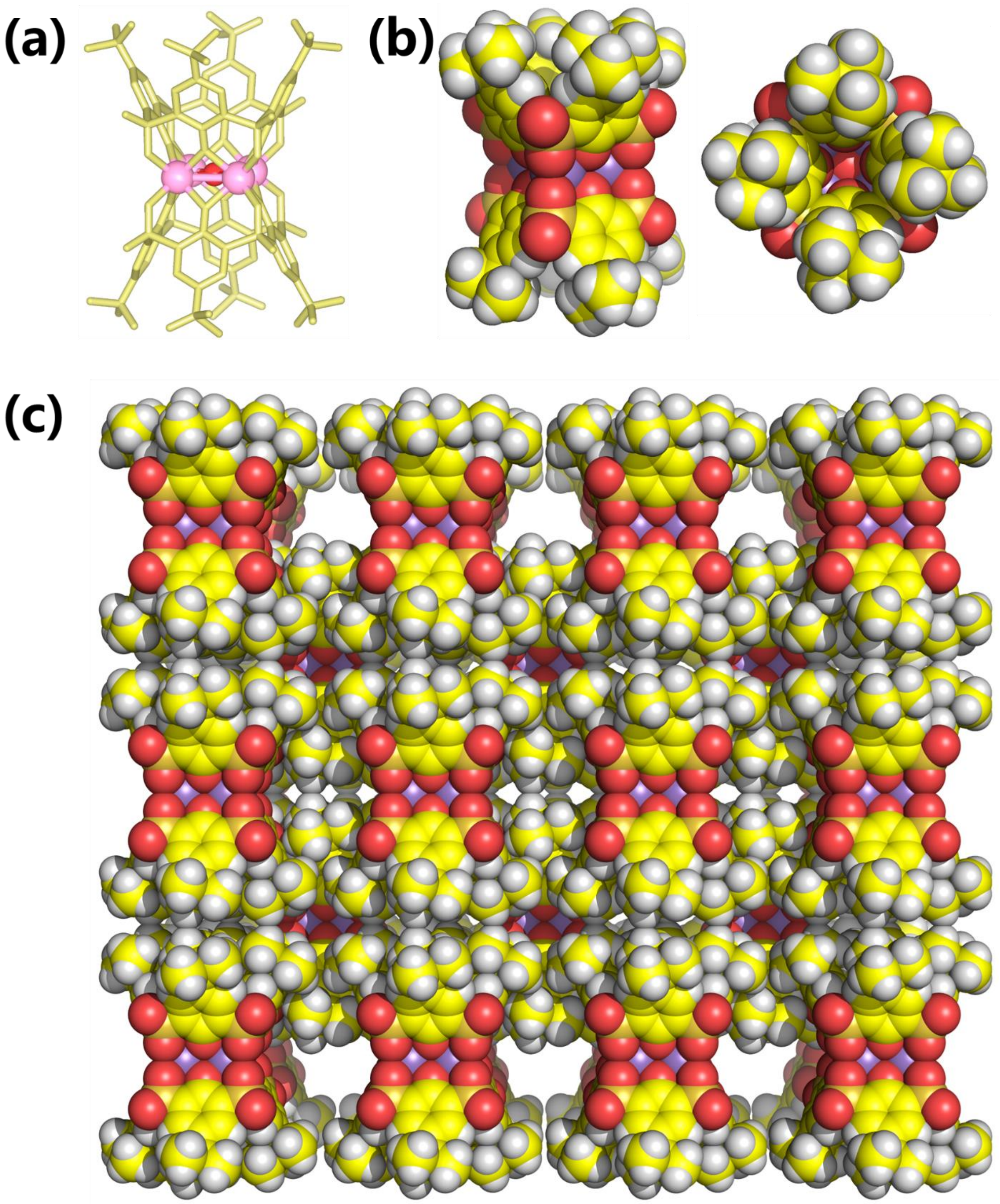


**Figure S4.** Structure (a) and different single view (b) and crystal packing view (c) of **Cluster-Mn** as determined by SC-XRD.

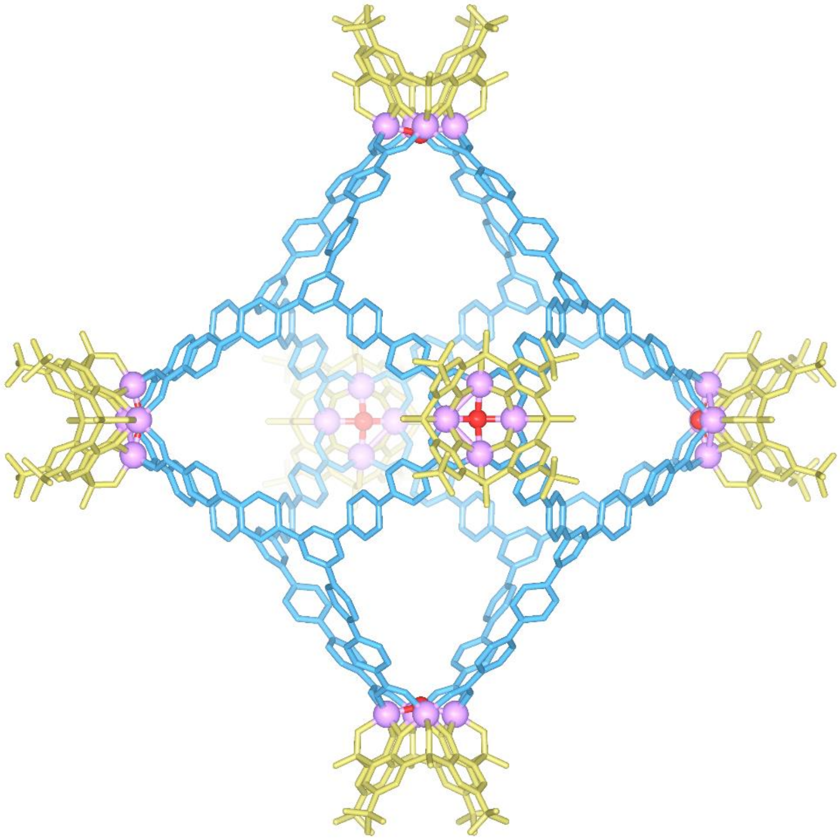

**Figure S5.** Structure of **PCC-12-Co** by SC-XRD.

**Table S1.** Crystal data and structure refinement for **PCC-10-Mn**.

| **Name** | **PCC-10-Mn** |
|---|---|
| Empirical formula | $C_{312}H_{288}Mn_{24}O_{12}6S_{24}$ |
| Formula weight | 8141.41 |
| Temperature/K | 273.15 |
| Crystal system | Tetragonal |
| Space group | *I*4/*m* |
| *a*/Å | 26.662 (7) |
| *b*/Å | 26.662 (7) |
| *c*/Å | 44.644 (16) |
| *α*/° | 90 |
| *β*/° | 90 |
| *γ*/° | 90 |
| Volume/Å$^3$ | 31736 (16) |
| Z | 2 |
| $\rho_{calc}$ (g cm$^{-3}$) | 0.852 |
| $\mu$/mm$^{-1}$ | 0.587 |
| F(000) | 8304.0 |
| Crystal size/mm$^3$ | 0.47 × 0.41 × 0.35 |
| Radiation | MoKα ($\lambda$ = 0.71073) |

| | |
|---|---|
| $2\Theta$ range for data collection/° | 4.24 to 50 |
| Index ranges | $-28 \le h \le 31$, $-31 \le k \le 29$, $-53 \le l \le 53$ |
| Reflections collected | 233501 |
| Independent reflections | 14109 [$R_{int}$ = 0.2074, $R_{sigma}$ = 0.0535] |
| Data/restraints/parameters | 14109/141/601 |
| Goodness-of-fit on $F^2$ | 1.137 |
| Final *R* indexes [I>=2σ (I)] | $R_1$ = 0.1073, w$R_2$ = 0.3726 |
| Final *R* indexes [all data] | $R_1$ = 0.1152, w$R_2$ = 0.4069 |
| Largest diff. peak/hole / e Å$^{-3}$ | 1.57/-1.26 |

Note: Values in the parentheses are the refinement factors before SQUEEZE.

**Table S2.** Crystal data and structure refinement for **PCC-11-Mn**.

| **Name** | **PCC-11-Mn** |
|---|---|
| Empirical formula | $C_{432}H_{360}Mn_{24}N_{24}O_{126}S_{24}$ |
| Formula weight | 9991.42 |
| Temperature/K | 173.00(10) |
| Crystal system | Tetragonal |
| Space group | *I*4/*m* |
| *a*/Å | 33.9461(14) |
| *b*/Å | 33.9461(14) |
| *c*/Å | 55.077(3) |
| *α*/° | 90 |
| *β*/° | 90 |
| *γ*/° | 90 |
| Volume/Å$^3$ | 63467(6) |
| Z | 2 |
| $\rho_{calc}$ (g cm$^{-3}$) | 0.523 |
| $\mu$/mm$^{-1}$ | 2.493 |
| F(000) | 10224.0 |
| Crystal size/mm$^3$ | 0.55 × 0.52 × 0.48 |
| Radiation | CuKα (λ = 1.54184) |

| | |
|---|---|
| $2\Theta$ range for data collection/° | 7.366 to 123.188 |
| Index ranges | $-32 \le h \le 27$, $-17 \le k \le 38$, $-62 \le l \le 60$ |
| Reflections collected | 50950 |
| Independent reflections | 23760 [Rint = 0.1118, Rsigma = 0.1343] |
| Data/restraints/parameters | 23760/303/811 |
| Goodness-of-fit on $F^2$ | 0.943 |
| Final *R* indexes [I>=2σ (I)] | $R_1$ = 0.0840, w$R_2$ = 0.2275 |
| Final *R* indexes [all data] | $R_1$ = 0.1257, w$R_2$ = 0.2543 |
| Largest diff. peak/hole / e Å$^{-3}$ | 0.61/-0.57 |

**Table S3.** Crystal data and structure refinement for **PCC-11-Co**.

| **Name** | **PCC-11- Co** |
|---|---|
| Empirical formula | $C_{432}H_{360}Co_{24}N_{24}O_{126}S_{24}$ |
| Formula weight | 10087.18 |
| Temperature/K | 100.00(10) |
| Crystal system | tetragonal |
| Space group | *I*4/*m* |
| *a*/Å | 33.0843(5) |
| *b*/Å | 33.0843(5) |
| *c*/Å | 54.196(2) |
| *α*/° | 90 |
| *β*/° | 90 |
| *γ*/° | 90 |
| Volume/Å$^3$ | 59321(3) |
| Z | 2 |
| $\rho_{calc}$ (g cm$^{-3}$) | 0.565 |
| $\mu$/mm$^{-1}$ | 0.400 |
| F(000) | 10320.0 |
| Crystal size/mm$^3$ | 0.38 × 0.27 × 0.23 |
| Radiation | Mo Kα ($\lambda$ = 0.71073) |

| | |
|---|---|
| 2$\Theta$ range for data collection/° | 6.886 to 49.998 |
| Index ranges | $-39 \leq h \leq 39$, $-39 \leq k \leq 39$, $-64 \leq l \leq 56$ |
| Reflections collected | 85422 |
| Independent reflections | 25225 [Rint = 0.1151, Rsigma = 0.1639] |
| Data/restraints/parameters | 25225/299/787 |
| Goodness-of-fit on $F^2$ | 1.033 |
| Final $R$ indexes [I>=2σ (I)] | $R_1$ = 0.1212, w$R_2$ = 0.3311 |
| Final $R$ indexes [all data] | $R_1$ = 0.1732, w$R_2$ = 0.3765 |
| Largest diff. peak/hole / e Å$^{-3}$ | 0.71/-0.65 |

**Table S4.** Crystal data and structure refinement for **PCC-11-Mg**.

| **Name** | **PCC-11-Mg** |
|---|---|
| Empirical formula | $C_{432}H_{360}Mg_{24}N_{24}O_{126}S_{24}$ |
| Formula weight | 9232.49 |
| Temperature/K | 100.00(10) |
| Crystal system | Tetragonal |
| Space group | *I*4/*m* |
| *a*/Å | 33.0174(7) |
| *b*/Å | 33.0174(7) |
| *c*/Å | 54.1314(13) |
| *α*/° | 90.0 |
| *β*/° | 90.0 |
| *γ*/° | 90.0 |
| Volume/Å$^3$ | 59011(2) |
| Z | 2 |
| $\rho_{calc}$ (g cm$^{-3}$) | 0.519 |
| $\mu$/mm$^{-1}$ | 0.804 |
| F(000) | 9600.0 |
| Crystal size/mm$^3$ | 0.35 × 0.32 × 0.21 |
| Radiation | Cu Kα ($\lambda$ = 1.54184) |

| | |
|---|---|
| 2$\Theta$ range for data collection/° | 7.5508 to 129.84 |
| Index ranges | $-31 \leq h \leq 22$, $-38 \leq k \leq 38$, $-63 \leq l \leq 62$ |
| Reflections collected | 101177 |
| Independent reflections | 22948 [$R_{int}$ = 0.0691, $R_{sigma}$ = 0.0515] |
| Data/restraints/parameters | 22948/169/823 |
| Goodness-of-fit on $F^2$ | 1.142 |
| Final $R$ indexes [I>=2σ (I)] | $R_1$ = 0.0966, w$R_2$ = 0.3024 |
| Final $R$ indexes [all data] | $R_1$ = 0.1208, w$R_2$ = 0.3241 |
| Largest diff. peak/hole / e Å$^{-3}$ | 0.46/-0.42 |

**Table S5.** Crystal data and structure refinement for **PCC-13-Mn**.

| Name | PCC-13-Mn |
|---|---|
| Empirical formula | $C_{456}H_{408}Mn_{24}N_{24}O_{126}S_{24}$ |
| Formula weight | 10328.04 |
| Temperature/K | 173.00(10) |
| Crystal system | Tetragonal |
| Space group | *I*4/*m* |
| *a*/Å | 33.8607(19) |
| *b*/Å | 33.8607(19) |
| *c*/Å | 55.247(3) |
| *α*/° | 90 |
| *β*/° | 90 |
| *γ*/° | 90 |
| Volume/Å$^3$ | 63344(8) |
| Z | 2 |
| $\rho_{calc}$ (g cm$^{-3}$) | 0.541 |
| $\mu$/mm$^{-1}$ | 2.505 |
| F(000) | 10608.0 |
| Crystal size/mm$^3$ | 0.21 × 0.18 × 0.15 |
| Radiation | Cu Kα ($\lambda$ = 1.54184) |

| | |
|---|---|
| $2\Theta$ range for data collection/° | 7.384 to 122.614 |
| Index ranges | $-38 \leq h \leq 33$, $-38 \leq k \leq 36$, $-46 \leq l \leq 62$ |
| Reflections collected | 57204 |
| Independent reflections | 23299 [$R_{int}$ = 0.1387, $R_{sigma}$ = 0.2086] |
| Data/restraints/parameters | 23299/791/973 |
| Goodness-of-fit on $F^2$ | 0.874 |
| Final $R$ indexes [I>=2σ (I)] | $R_1$ = 0.0844, w$R_2$ = 0.2152 |
| Final $R$ indexes [all data] | $R_1$ = 0.1691, w$R_2$ = 0.2600 |
| Largest diff. peak/hole / e Å$^{-3}$ | 0.44/-0.31 |

**Table S6.** Crystal data and structure refinement for **PCC-13-Co**.

| Name | PCC-13-Co |
|---|---|
| Empirical formula | $C_{456}H_{408}Co_{24}N_{24}O_{126}S_{24}$ |
| Formula weight | 10423.80 |
| Temperature/K | 100.00(10) |
| Crystal system | Tetragonal |
| Space group | $I4/m$ |
| $a$/Å | 32.9556(7) |
| $b$/Å | 32.9556(7) |
| $c$/Å | 54.5250(11) |
| $\alpha$/° | 90 |
| $\beta$/° | 90 |
| $\gamma$/° | 90 |
| Volume/Å$^3$ | 59218(3) |
| Z | 2 |
| $\rho_{calc}$ (g cm$^{-3}$) | 0.585 |
| $\mu$/mm$^{-1}$ | 0.402 |
| F(000) | 10704.0 |
| Crystal size/mm$^3$ | 0.67 × 0.57 × 0.31 |
| Radiation | Mo Kα ($\lambda$ = 0.71073) |

| | |
|---|---|
| $2\Theta$ range for data collection/° | 6.676 to 50.052 |
| Index ranges | $-39 \le h \le 31$, $-38 \le k \le 39$, $-64 \le l \le 64$ |
| Reflections collected | 124926 |
| Independent reflections | 26404 [$R_{int}$ = 0.0914, $R_{sigma}$ = 0.0787] |
| Data/restraints/parameters | 26404/608/936 |
| Goodness-of-fit on $F^2$ | 1.084 |
| Final $R$ indexes [I>=2σ (I)] | $R_1$ = 0.0803, w$R_2$ = 0.2462 |
| Final $R$ indexes [all data] | $R_1$ = 0.1197, w$R_2$ = 0.2762 |
| Largest diff. peak/hole / e Å$^{-3}$ | 0.85/-0.45 |

**Table S7.** Crystal data and structure refinement for **PCC-13-Mg**.

| **Name** | **PCC-13-Mg** |
|---|---|
| Empirical formula | $C_{432}H_{360}Mg_{24}N_{24}O_{126}S_{24}$ |
| Formula weight | 9592.92 |
| Temperature/K | 173.00(10) |
| Crystal system | tetragonal |
| Space group | *I*4/*m* |
| *a*/Å | 33.5540(12) |
| *b*/Å | 33.5540(12) |
| *c*/Å | 54.7983(17) |
| *α*/° | 90 |
| *β*/° | 90 |
| *γ*/° | 90 |
| Volume/Å$^3$ | 61696(5) |
| Z | 2 |
| $\rho_{calc}$ (g cm$^{-3}$) | 0.516 |
| $\mu$/mm$^{-1}$ | 0.783 |
| F(000) | 9984.0 |
| Crystal size/mm$^3$ | 0.45 × 0.38 × 0.32 |
| Radiation | Cu Kα ($\lambda$ = 1.54184) |

| | |
|---|---|
| $2\Theta$ range for data collection/° | 7.452 to 122.796 |
| Index ranges | $-32 \le h \le 36$, $-38 \le k \le 29$, $-62 \le l \le 62$ |
| Reflections collected | 73461 |
| Independent reflections | 22741 [$R_{int}$ = 0.0793, $R_{sigma}$ = 0.0831] |
| Data/restraints/parameters | 22741/776/962 |
| Goodness-of-fit on $F^2$ | 0.990 |
| Final *R* indexes [I>=2σ (I)] | $R_1$ = 0.0904, w$R_2$ = 0.2670 |
| Final *R* indexes [all data] | $R_1$ = 0.1270, w$R_2$ = 0.2971 |
| Largest diff. peak/hole / e Å$^{-3}$ | 0.50/-0.37 |

## Section 3. Characterization of properties for material.

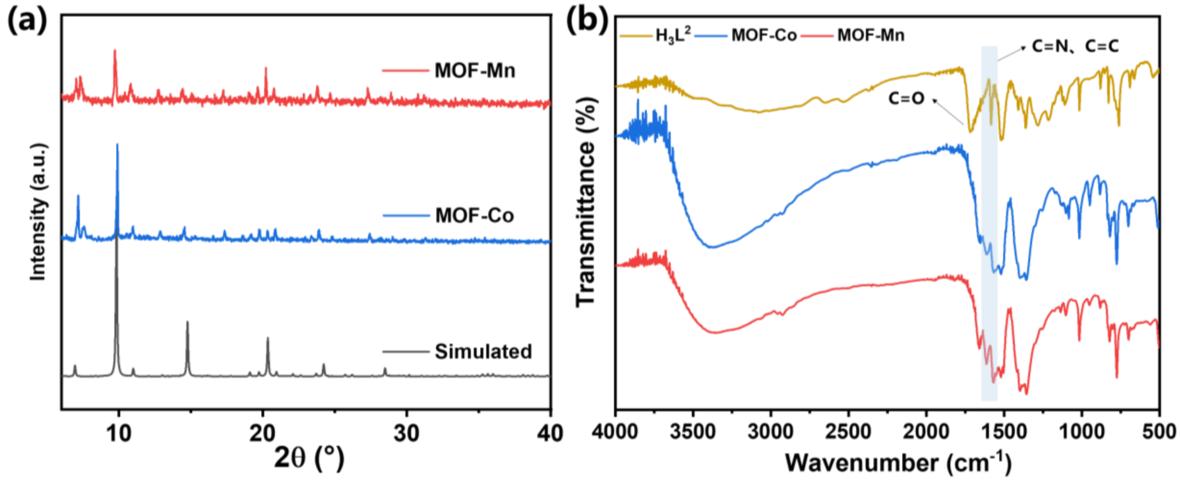


**Figure S6.** (a) PXRD patterns of simulated PCN-9-Co, MOF-Co, and MOF-Mn. (b) FT-IR spectra of $H_3L^2$, MOF-Co, and MOF-Mn.

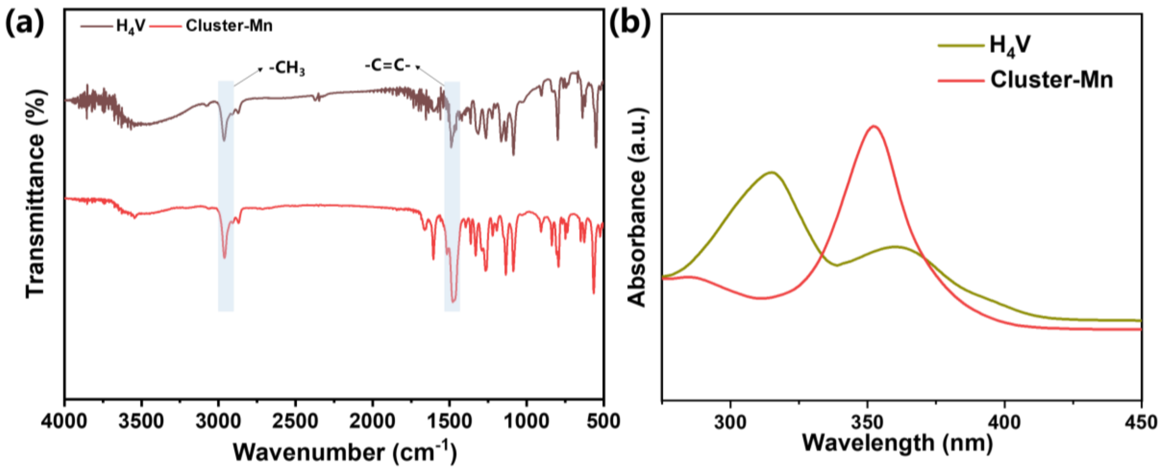


**Figure S7.** FT-IR spectra (a) and UV spectra (b) of $H_4V$, and Cluster-Mn.

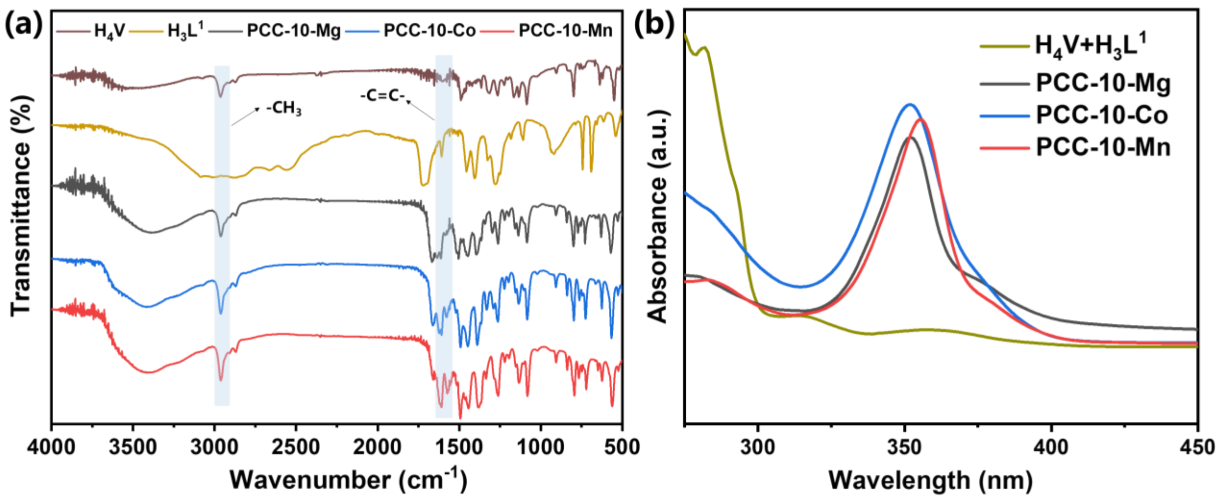


**Figure S8.** FT-IR spectra (a) and UV spectra (b) of $H_4V$, $H_3L^1$, PCC-10-Mg, PCC-10-Co, and PCC-10-Mn.

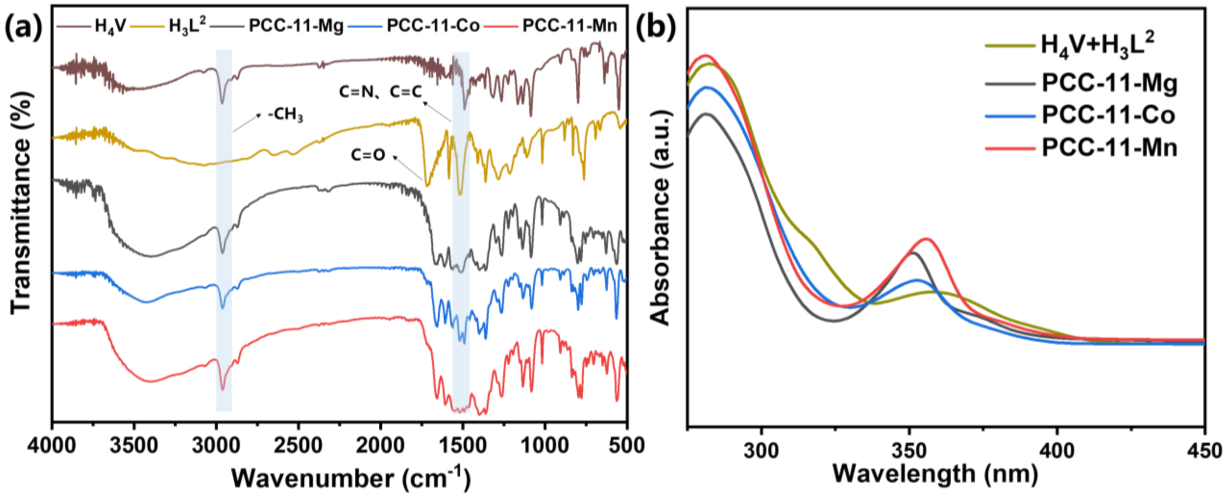


**Figure S9.** FT-IR spectra (a) and UV spectra (b) of $H_4V$, $H_3L^2$, PCC-11-Mg, PCC-11-Co, and PCC-11-Mn.

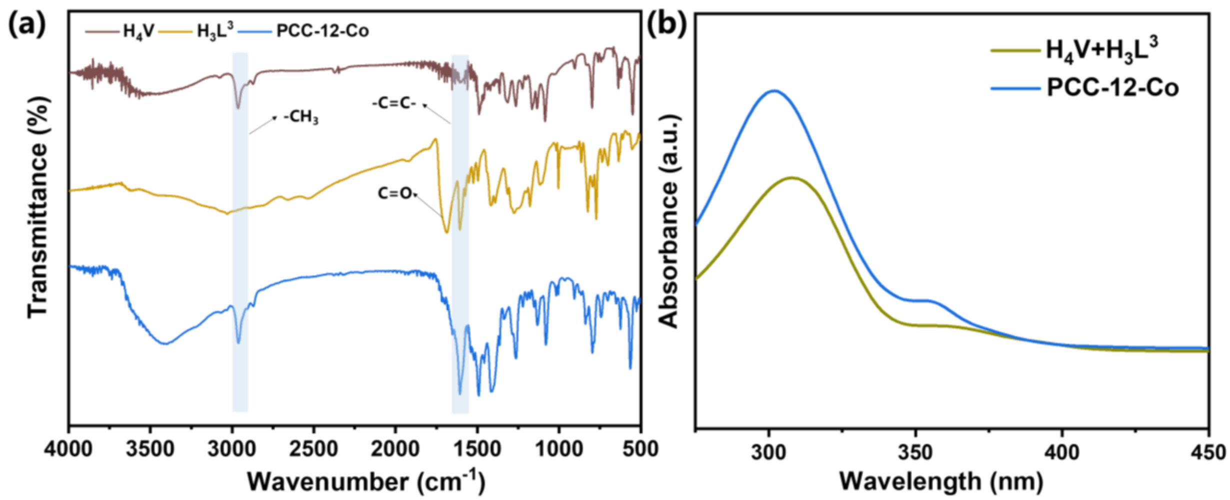


**Figure S10.** FT-IR spectra (a) and UV spectra (b) of $H_4V$, $H_3L^3$, and PCC-12-Co.

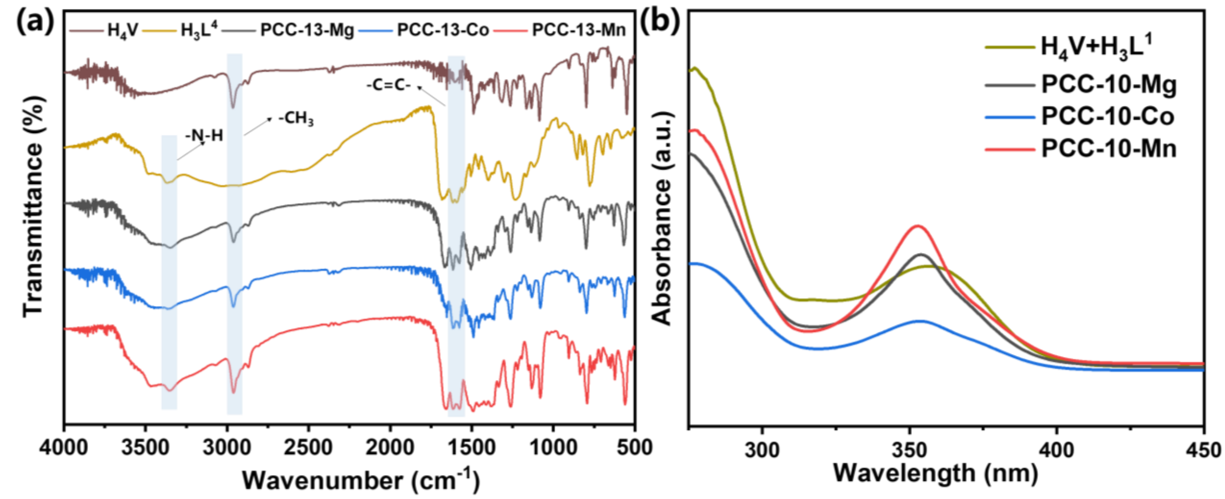


**Figure S11.** FT-IR spectra (a) and UV spectra (b) of $H_4V$, $H_3L^4$, PCC-13-Mg, PCC-13-Co, and PCC-13-Mn.

**PCC**, **MOF,** and **Cluster** have been verified to be successfully synthesized by Figure S6~S11. The PXRD patterns clearly show the characteristic peaks of **MOF-Mn** and **MOF-Co** very similar to simulated **PCN-9-Co**, which also corresponds to -C=N, -C=C- in the FT-IR spectra. The FT-IR spectra of PCC and Cluster compared to the relative ligand likewise verified the accurate synthesis. Moreover, as soluble discrete structured molecules, PCC and Cluster can be corroborated by UV tests with characteristic peaks on the same wavelength.

## Section 4. $N_2$ adsorption-desorption measurements of PCCs

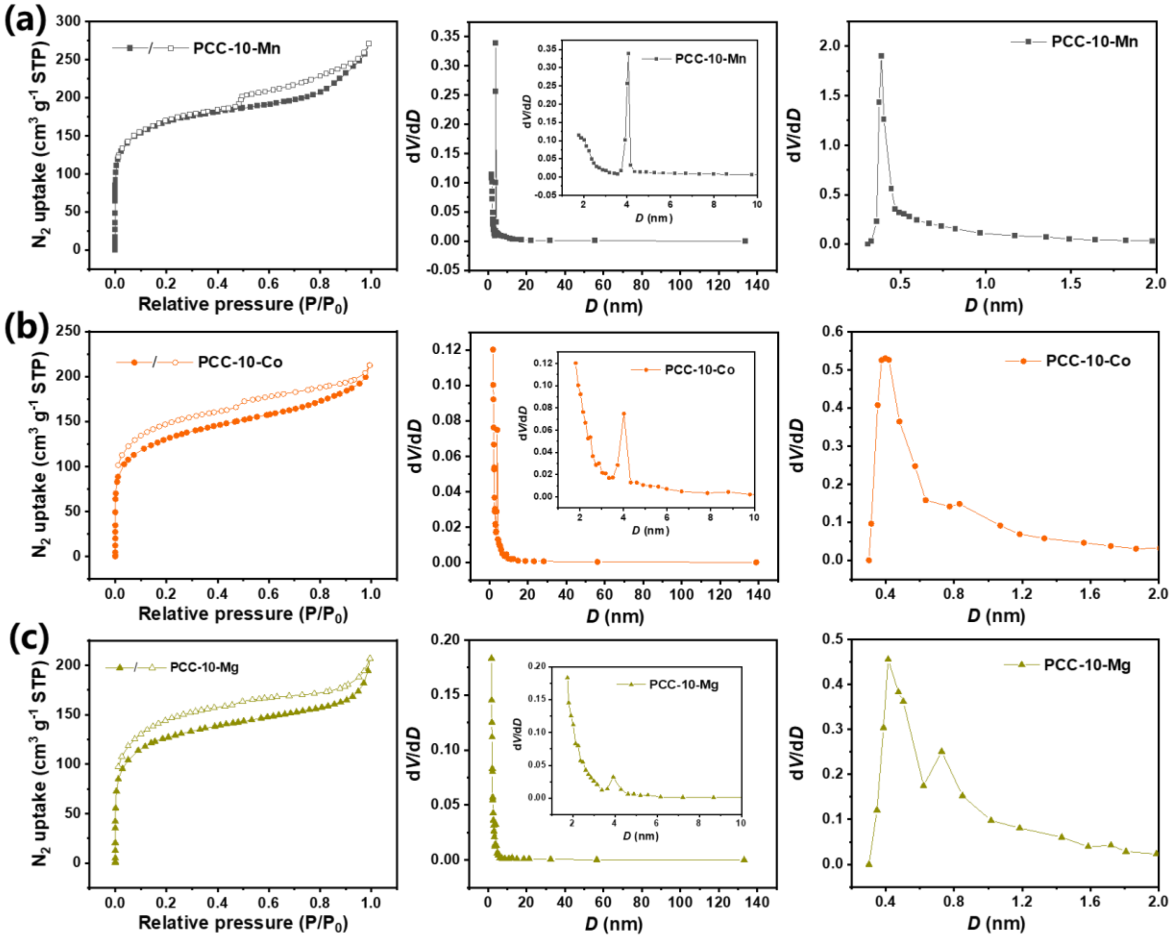


**Figure S12.** $N_2$ adsorption-desorption isotherms (left), BJH pore-size distribution (middle) and NLDFT pore-size distributions (right) of **PCC-10-Mn** (a), **PCC-10-Co** (b), and **PCC-10-Mg** (c).

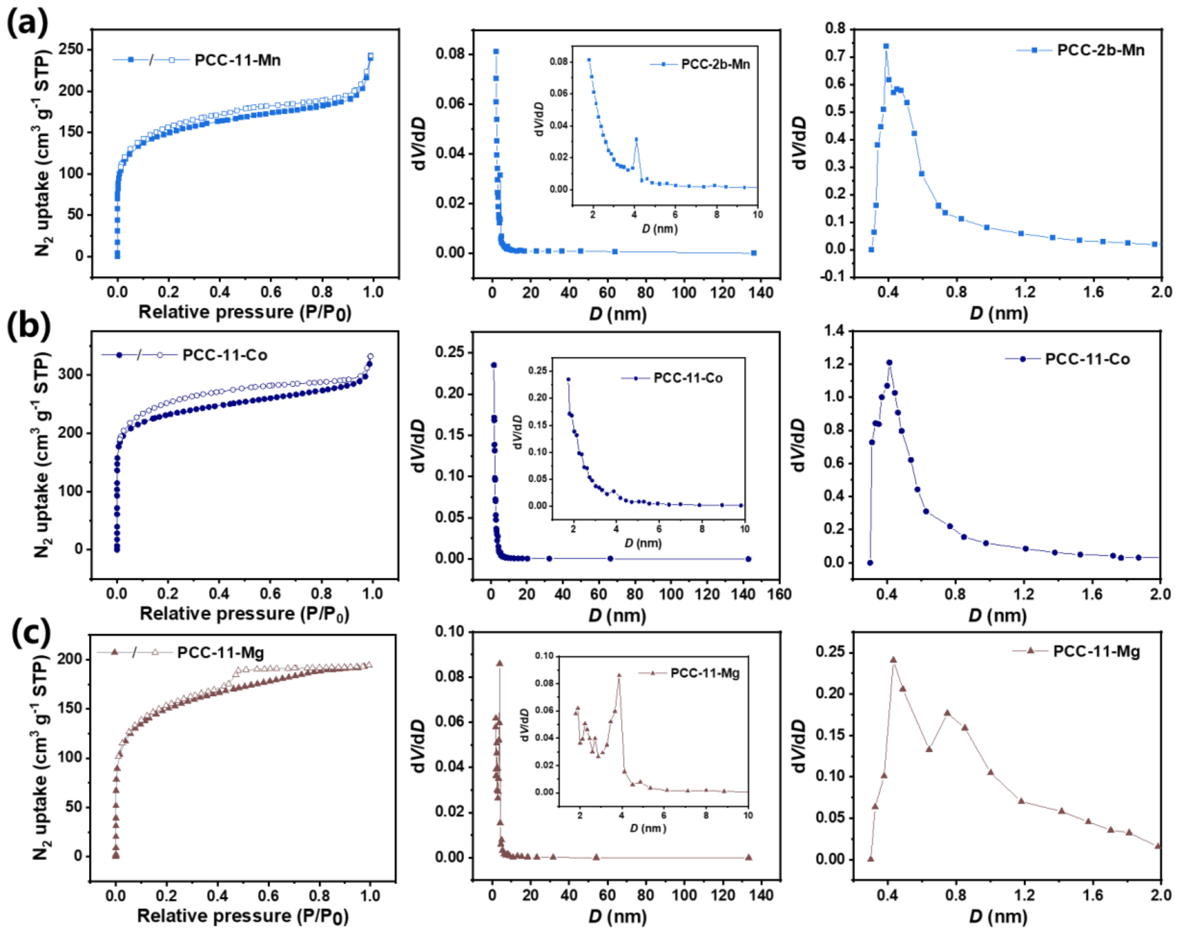


**Figure S13.** $N_2$ adsorption-desorption isotherms (left), BJH pore-size distribution (middle), and NLDFT pore-size distributions (right) of **PCC-11-Mn** (a), **PCC-11-Co** (b), and **PCC-11-Mg** (c).

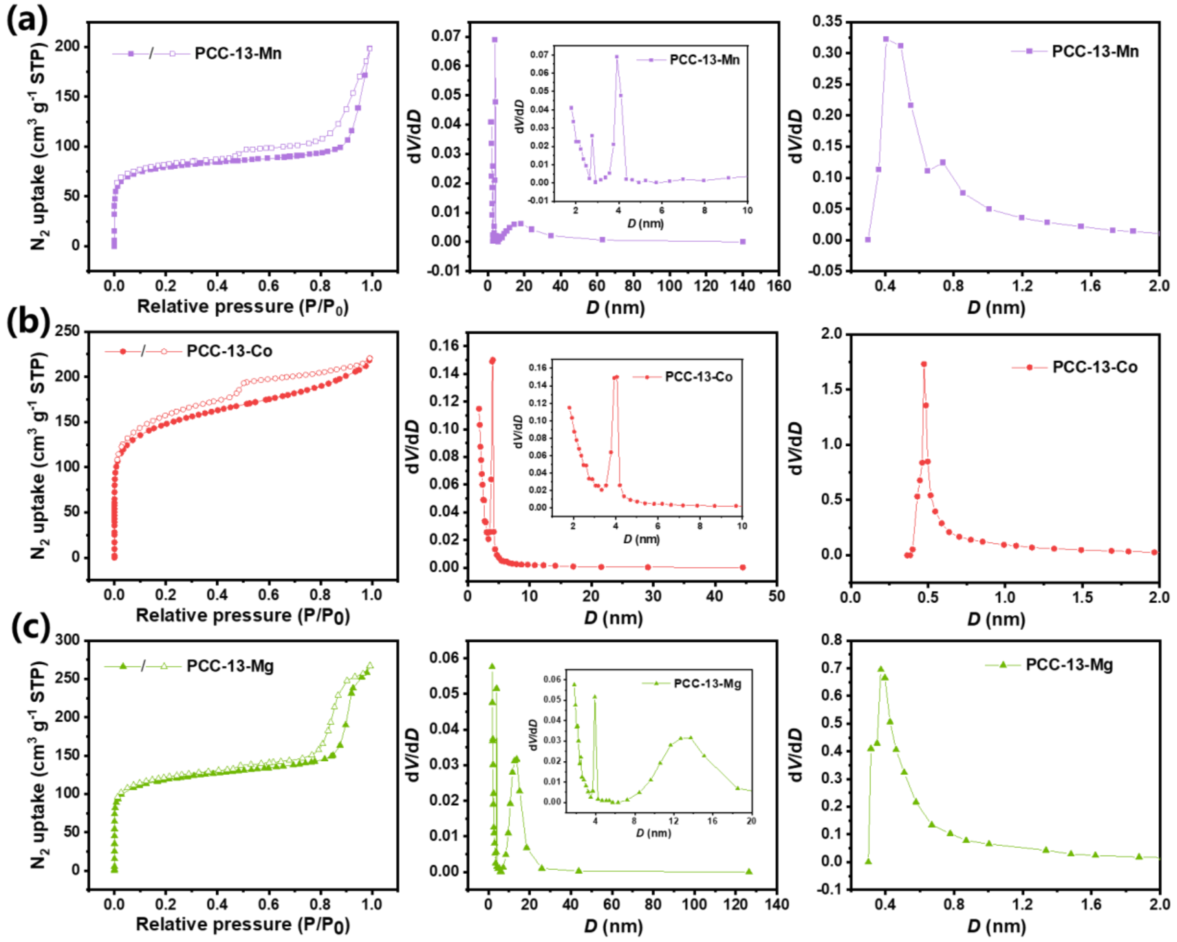


**Figure S14.** $N_2$ adsorption-desorption isotherms (left), BJH pore-size distribution (middle), and NLDFT pore-size distributions (right) of **PCC-13-Mn** (a), **PCC-13-Co** (b), and **PCC-13-Mg** (c).

**Table S8.** Pore structure parameters of PCCs.

| Sample | $S_{BET}$[a] ($m^2$ $g^{-1}$) | $V_{Total}$[b] ($cm^3$ $g^{-1}$) | $V_{Micro}$[c] ($cm^3$ $g^{-1}$) | $V_{Meso}$[d] ($cm^3$ $g^{-1}$) | $D_{BET}$[e] (nm) |
|---|---|---|---|---|---|
| **PCC-10-Mg** | 463 | 0.31 | 0.12 | 0.19 | 2.67 |
| **PCC-10-Co** | 471 | 0.32 | 0.13 | 0.19 | 2.76 |
| **PCC-10-Mn** | 609 | 0.42 | 0.16 | 0.26 | 2.74 |
| **PCC-11-Mg** | 358 | 0.22 | 0.09 | 0.13 | 2.47 |
| **PCC-11-Co** | 632 | 0.33 | 0.20 | 0.13 | 2.06 |
| **PCC-11-Mn** | 740 | 0.38 | 0.25 | 0.13 | 2.05 |
| **PCC-13-Mg** | 439 | 0.41 | 0.12 | 0.29 | 3.74 |
| **PCC-13-Co** | 448 | 0.27 | 0.15 | 0.12 | 2.41 |
| **PCC-13-Mn** | 294 | 0.30 | 0.08 | 0.22 | 4.11 |

[a] Specific surface area calculated by the BET method.

[b] Total pore volume at a relative pressure of $P/P_0$ =0.99.

[c] Micropore volume calculated from $N_2$ sorption isotherms by T-Plot method.

[d] Mesopore volume calculated from $N_2$ sorption isotherms by BJH method.

[e] Average pore size distribution by BET method.

The specific surface areas of PCC-10, PCC-11, and PCC-13 molecular cages were mostly 300~500 $m^2$ $g^{-1}$, and the average pore diameter was 2~3 nm. It is well known that compared with micropores, mesopores are more conducive to ion diffusion during charge and discharge. The mesopore volume of these PCCs is about 0.2 $cm^3$ $g^{-1}$, which provides an effective transmission pathway for the EDLC process.

## Section 5. Electrochemical analysis of PCCs, MOFs, and Clusters.

The electrochemical performances of PCCs were evaluated through a three-electrode system in a neutral aqueous electrolyte (3 M KCl). The PCC microcrystals were coated on graphite paper (with a loading of 1 mg $cm^{-2}$) as the working electrode. A saturated calomel electrode and a platinum foil were applied as the reference electrode and counter electrode, respectively. All built test systems were kept at ambient temperature (25 $^{o}$C) and pressure (101.325 KPa) for 2 h before the measurement.

Cyclic voltammetry (CV) measurements are performed to get a preliminary understanding of the charge storage behavior of these electrodes in the potential range of 0–1.0 V at a scan rate of 100 mV $s^{-1}$. The specific capacities were obtained by galvanostatic charge-discharge (GCD) measurements at 0.5 A $g^{-1}$.

The peak current $i$ in a CV curve can be related to the scan rate $v$ by the equation $i = av^b$ (or $\log i = b \log v + \log a$), where a is a coefficient, and b value is a constant constrained to fall in the range of 0.5~1. [19, 30] When the b value approaches 0.5, the charge storage mechanism of the materials is dominated by the diffusion-controlled process; while the b value approaches 1, the charge storage mechanism is dominated by the pseudocapacitive process of fast redox reaction. The current contributions of the capacitive process and the diffusion-controlled process can be separated using Dunn's method. Capacitive and diffusion contributions to the total capacity at different scan rates are calculated based on CV curves.

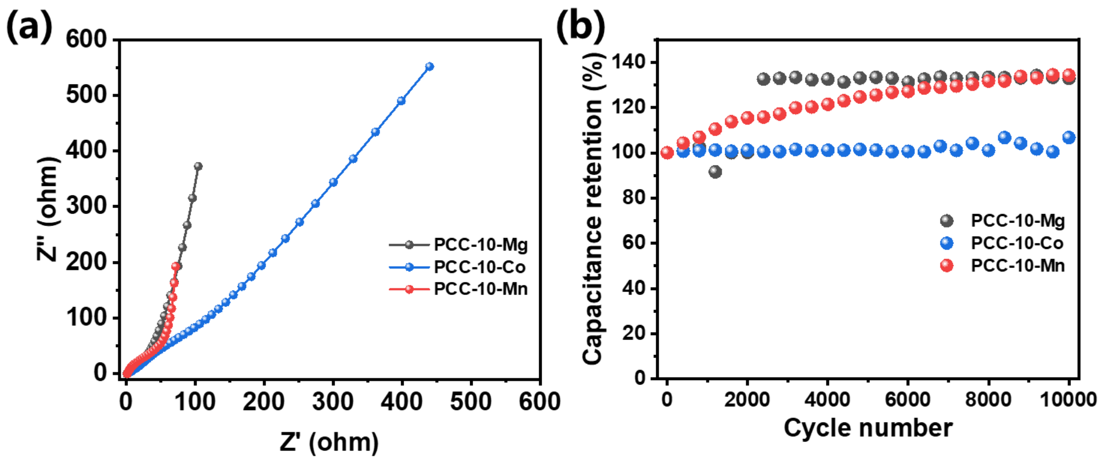


**Figure S15.** (a) Nyquist plots of **PCC-10**. (b) The cycling performance of **PCC-10** was measured at 10 A $g^{-1}$ for 10000 cycles.

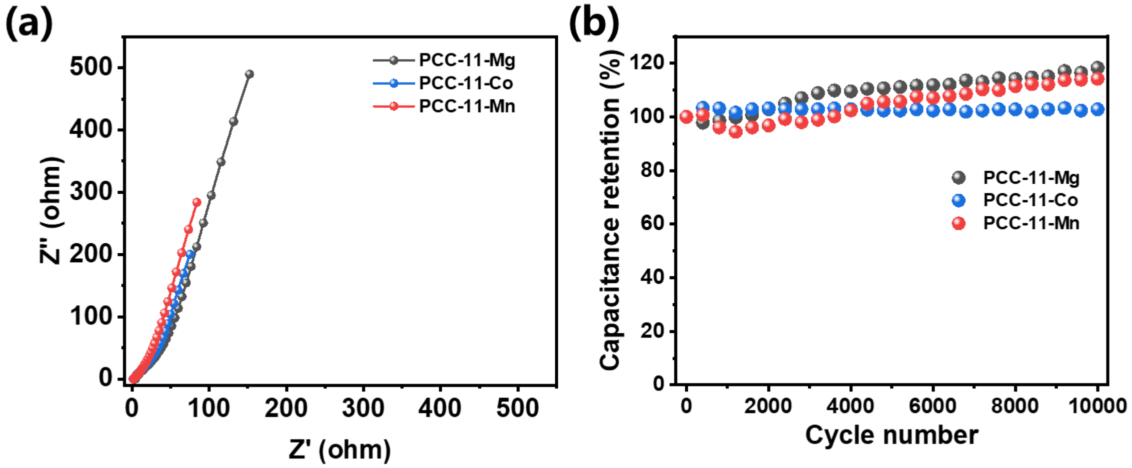


**Figure S16.** (a) Nyquist plots of **PCC-11**. (b) The cycling performance of **PCC-11** was measured at 10 A $g^{-1}$ for 10000 cycles.

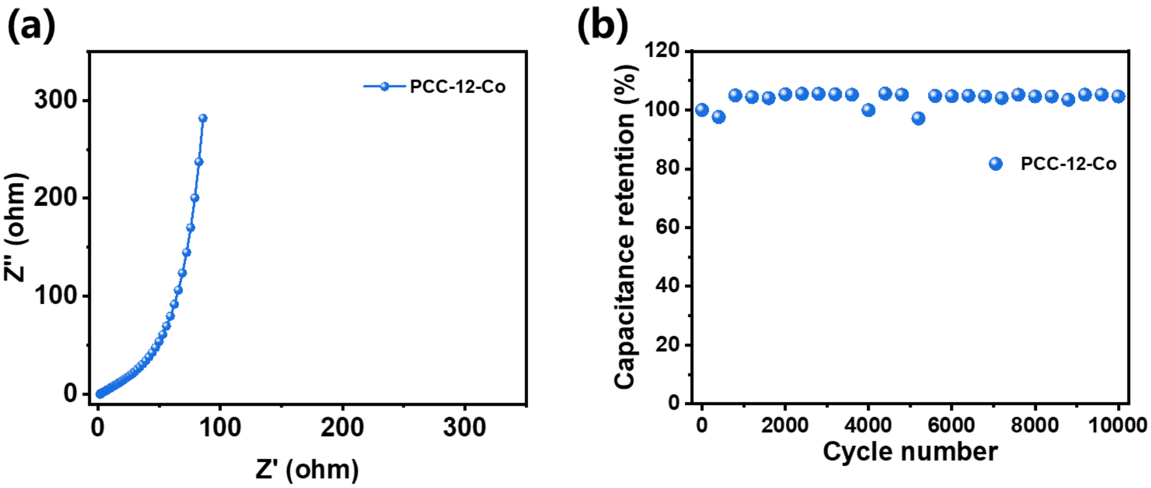


**Figure S17.** (a) Nyquist plots of **PCC-12-Co**. (b) The cycling performance of **PCC-12-Co** was measured at 10 A $g^{-1}$ for 10000 cycles.

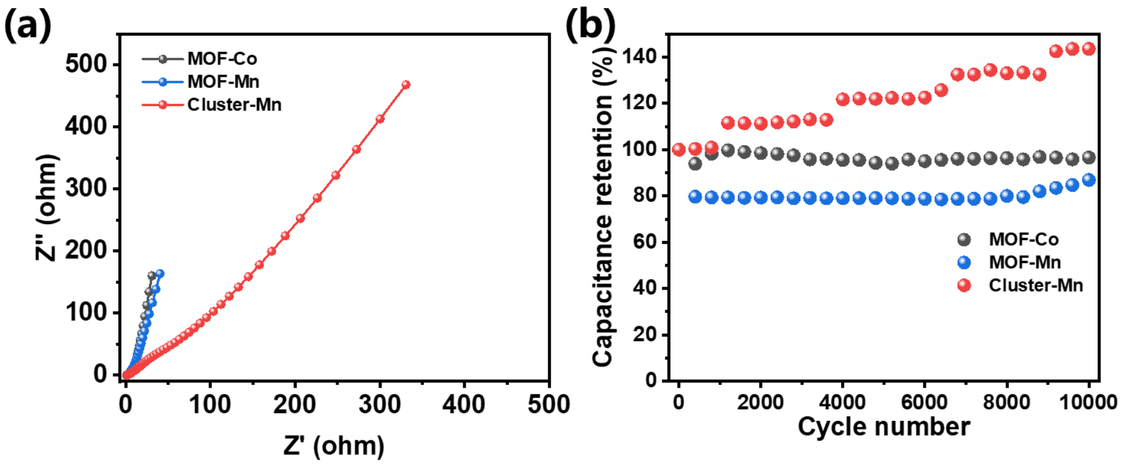


**Figure S18.** (a) Nyquist plots of **MOF-M** and **Cluster-Mn**. (b) The cycling performance of **MOF-M** and **Cluster-Mn** was measured at 10 A $g^{-1}$ for 10000 cycles.

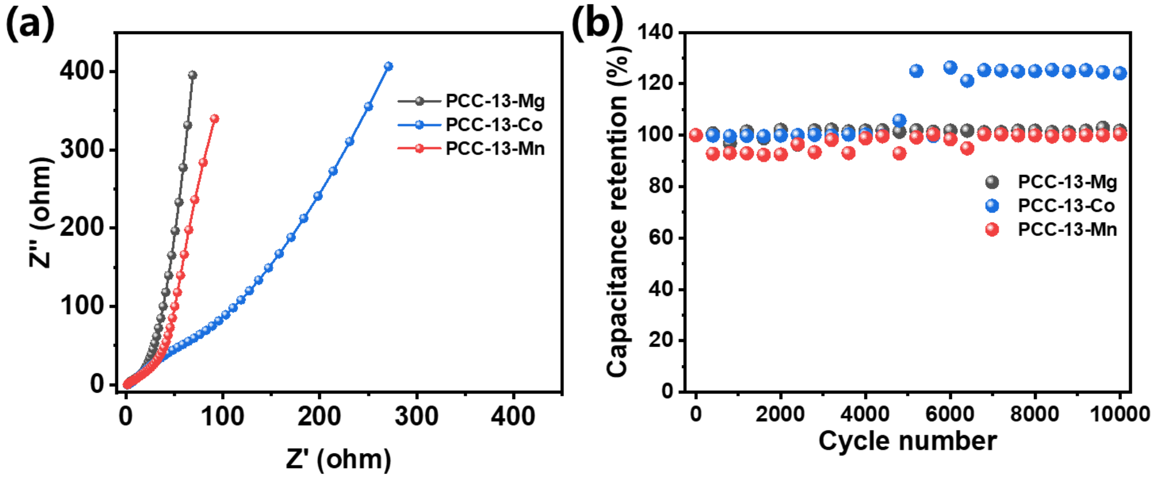


**Figure S19.** (a) Nyquist plots of **PCC-13**. (b) The cycling performance of **PCC-13** was measured at 10 A $g^{-1}$ for 10000 cycles.

## Section 6. Summary and comparison of performance.

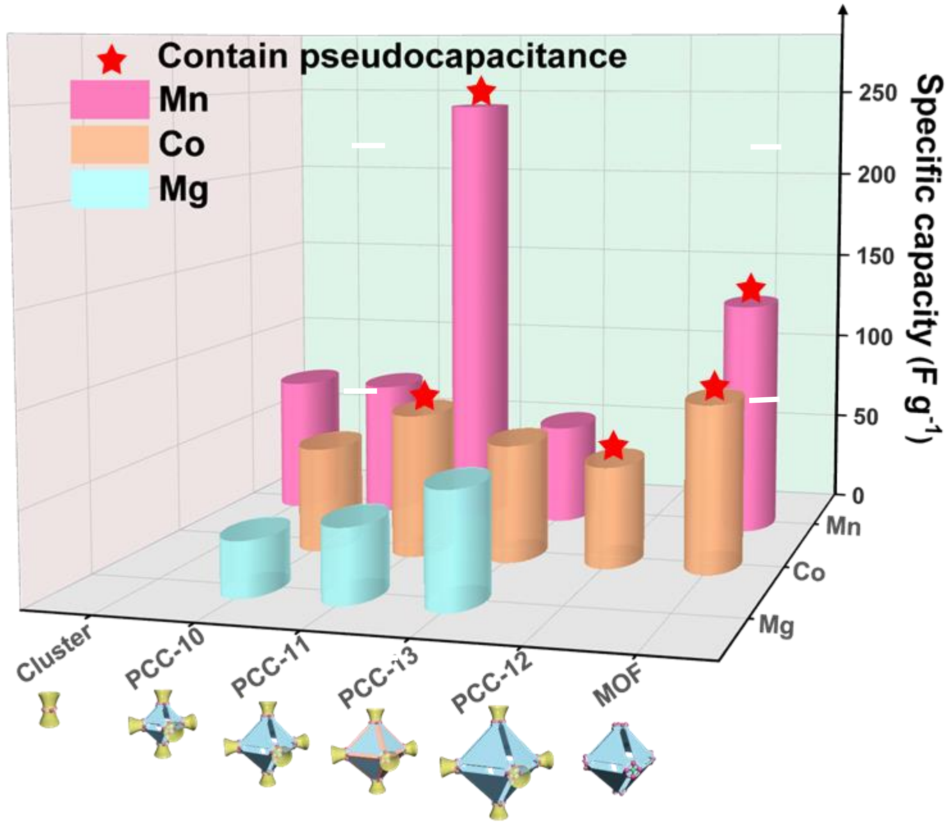


**Figure S20.** Summary of specific capacity for PCCs, MOFs, and Clusters.

It is observed that **MOF-M** (M = Mn, Co) with only the inner cavity and Cluster-M (M = Mn) with only the external cavity hardly catch up with the PCCs with both inner and external cavities in terms of molar-specific capacity. As an infinite network, **MOF-M** exhibited a gradual efficiency loss after cycles. However, **Cluster-M** can maintain the performance even after 10,000 cycles, which could be attributed to the intrinsic merit of the discrete structure. Indeed, the performance enhancement after pre-activation by a small current can be also observed for all the PCC electrodes. As the smallest cousin in the PCC family, **PCC-10** showed the lowest molar capacity, probably because the small aperture prohibited the entrance of electrolyte ions ($K^+$) (Figure 3H). On the other hand, **PCC-12-Co** has the largest inner cavity, but the molar-specific capacitance is only 80% of that of **PCC-11-Co**

(Figure 3H). The reason should be the decreased metal density in the enlarged cavity, followed by reducing the pseudocapacitance contribution per gram. Although the **PCC-13** series share the same inner and external cavity as the **PCC-11** series, the molar capacity of the former is still much lower than that of the latter (Figure 3H, Table S9). This could be explained that the amino groups at the aperture of the cavity sealed the inner cavity which in turn prohibited the entrance of the charge carrier. In the same category of **PCC-11**, the redox activity of the metal center is crucial for achieving high capacity (Figure 3H). For example, **PCC-11-Mg** exhibits the lowest specific capacitance among the **PCC-11-M** series, which is due to the Mg center hardly undergoing valence change at the specific current. **PCC-11-Mn** and **PCC-11-Co** ranked top two (Figure S20) among all the analyzed materials because they possess all necessary criteria such as discrete structure, available inner cavity, external cavity, and redox center. Given that the molecular weight of **PCC-11-Mn** and **PCC-11-Co** is almost identical, the capacitance of **PCC-11-Mn** is 3-fold more than that of Co one. The difference in capacitance for the same cage structure with different metal centers indicated that the Mn center may store more electrons than the Co center. Since the metal sources for constructing PCCs have the same valence ($Mn^{2+}$ and $Co^{2+}$), it strongly suggested that the Mn center underwent a higher valence change during the charge-discharge process than that of Co (Figure 3H).

**Table S9.** Comparison of gravimetric capacity and molecular capacity.

| Sample | Empirical formula | Formula weight | Gravimetric capacity (F $g^{-1}$) | Molecular capacity (F $mmol^{-1}$) |
|---|---|---|---|---|
| Cluster-Mn | $C_{80}H_{88}Mn_4O_{25}S_8$ | 1925.79 | 75 | 144.43 |
| PCC-10-Co | $C_{312}H_{300}Co_{24}O_{126}S_{24}$ | 8249.28 | 57 | 470.21 |
| PCC-11-Mg | $C_{456}H_{408}Mg_{24}N_{24}O_{126}S_{24}$ | 9593.08 | 40 | 383.72 |
| MOF-Co | $C_{192}Co_{12}H_{96}N_{24}O_{51}$ | 4262.10 | 91 | 387.85 |
| PCC-10-Mg | $C_{312}H_{300}Mg_{24}O_{126}S_{24}$ | 7418.40 | 30 | 222.55 |
| MOF-Mn | $C_{192}Mn_{12}H_{96}N_{24}O_{51}$ | 4214.16 | 130 | 547.84 |
| PCC-13-Mn | $C_{480}H_{456}Mn_{24}N_{24}O_{126}S_{24}$ | 10664.92 | 55 | 586.57 |
| PCC-10-Mn | $C_{312}H_{300}Mn_{24}O_{126}S_{24}$ | 8153.66 | 75 | 611.52 |
| PCC-13-Mg | $C_{480}H_{456}Mg_{24}N_{24}O_{126}S_{24}$ | 9929.73 | 62 | 615.64 |
| PCC-12-Co | $C_{600}H_{504}Co_{24}O_{126}S_{24}$ | 11914.34 | 55 | 655.29 |
| PCC-13-Co | $C_{480}H_{456}Co_{24}N_{24}O_{126}S_{24}$ | 10759.84 | 64 | 688.63 |
| PCC-11-Co | $C_{456}H_{408}Co_{24}N_{24}O_{126}S_{24}$ | 10424.15 | 78 | 813.08 |
| **PCC-11-Mn** | $\mathbf{C_{456}H_{408}Mn_{24}N_{24}O_{126}S_{24}}$ | **10328.27** | **243** | **2509.77** |

## Section 7. XPS spectra of PCCs, MOFs, and Clusters.

The pseudocapacitive energy storage process of **PCC-11-Mn** is well illustrated by tracking the change of valence and coordination mode of Mn and O: it is the "$Mn^{4+}$-$Mn^{3+}$ reversible interchange" accompanied by the coordination of $K^+$ with the $\mu_4$-O center in the electrolyte, which realizes the efficient energy storage of the electrode material, and proves its potential to develop high-performance supercapacitors. Excellent electrochemical stability is also verified by the reversible valence transformation within the molecule throughout the charging and discharging process.

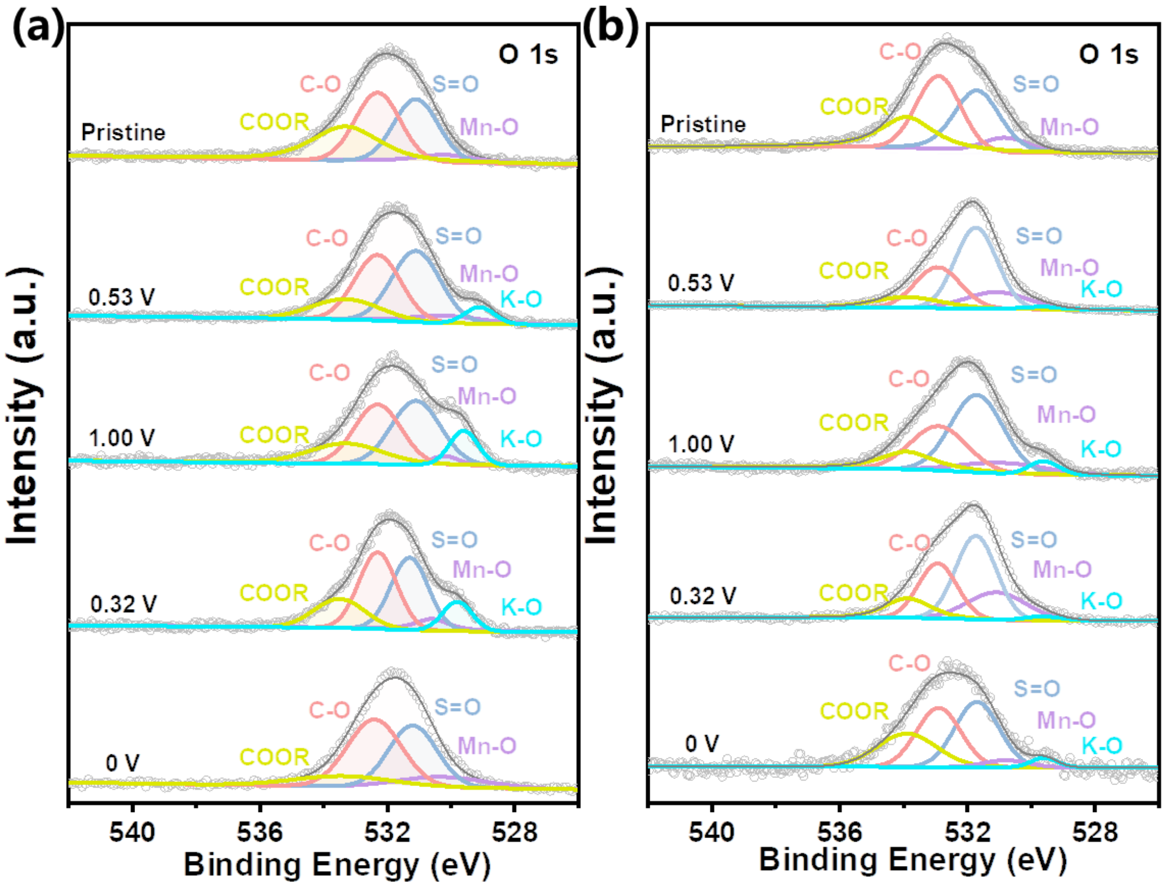


**Figure S21.** Ex situ XPS O 1s spectra of PCC-11-Mn (a) and **MOF-Mn** (b) at different charged and discharged states.

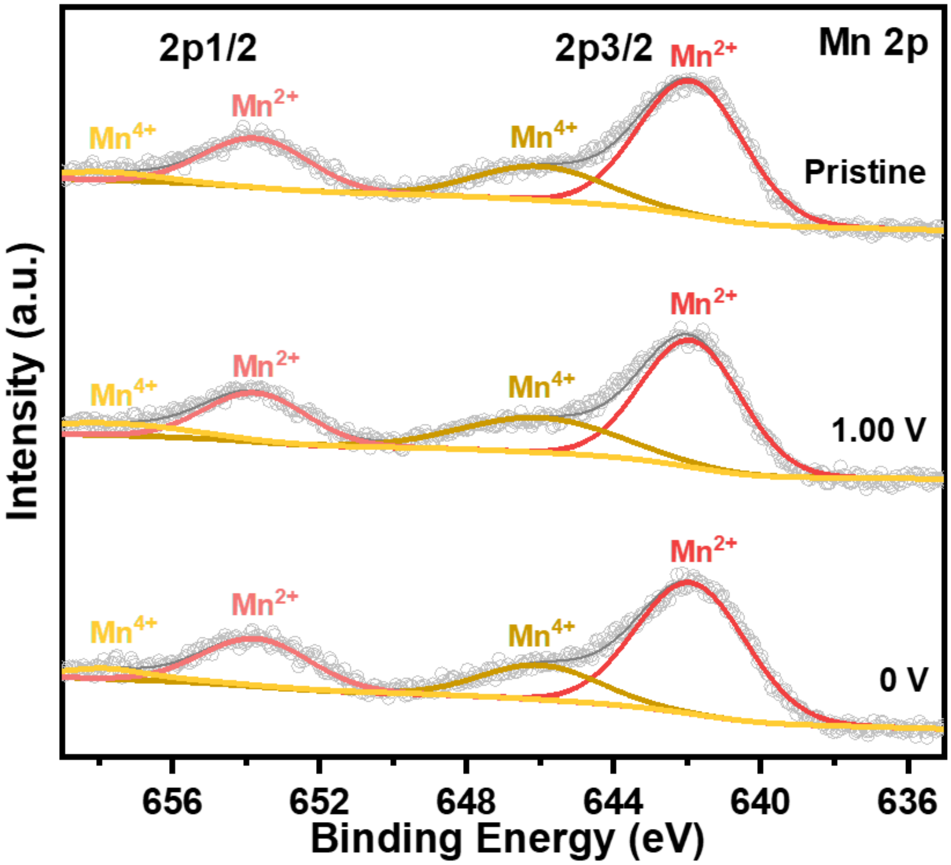


**Figure S22.** Ex situ XPS Mn 2p spectra of **Cluster-Mn** at different charged and discharged states.

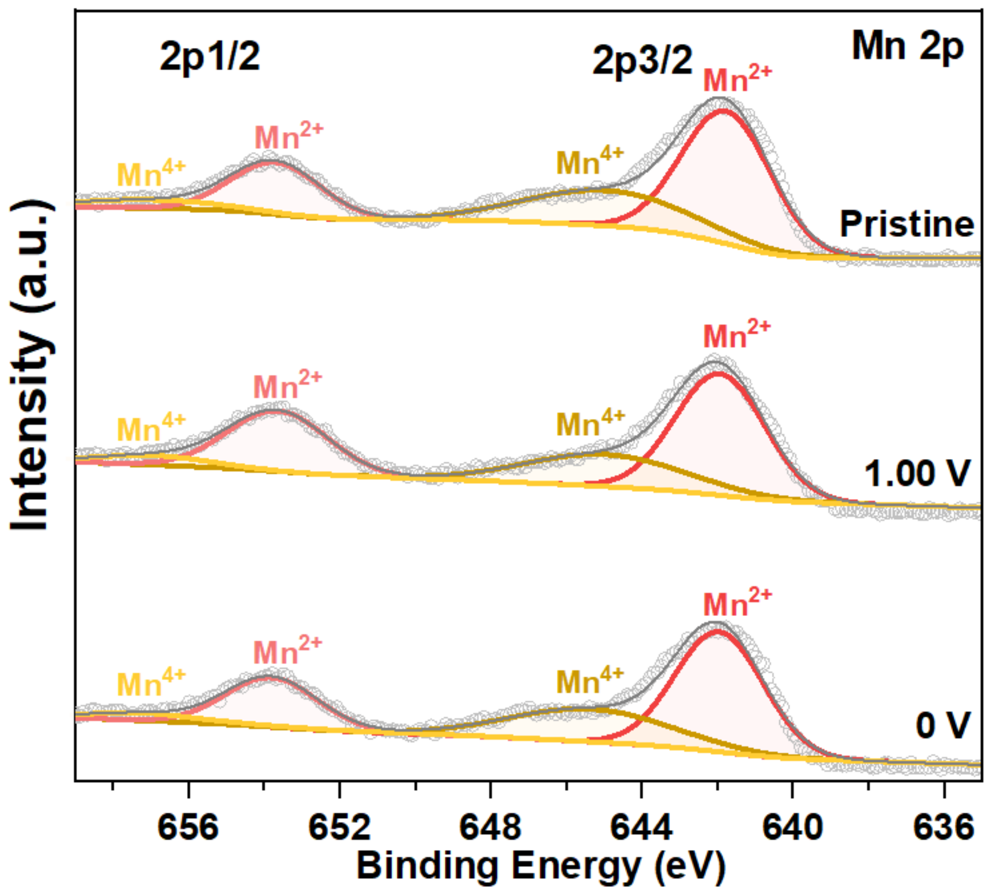


**Figure S23.** Ex situ XPS Mn 2p spectra of **PCC-10-Mn** at different charged and discharged states.

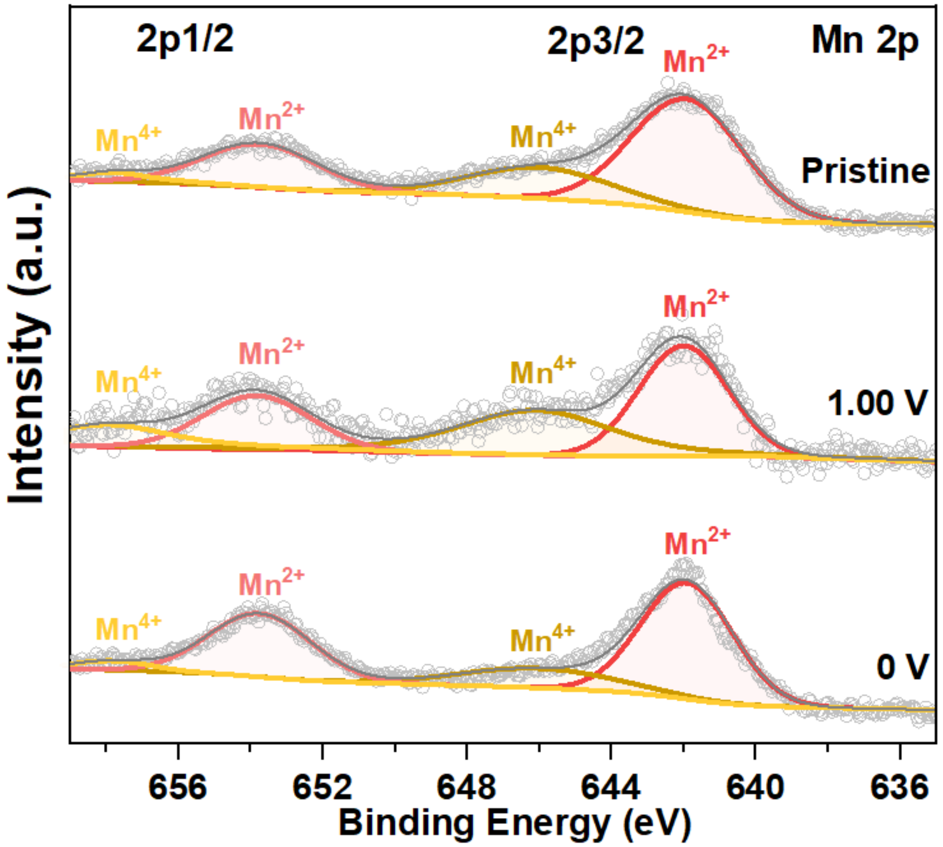


**Figure S24.** Ex situ XPS Mn 2p spectra of **PCC-13-Mn** at different charged and discharged states.

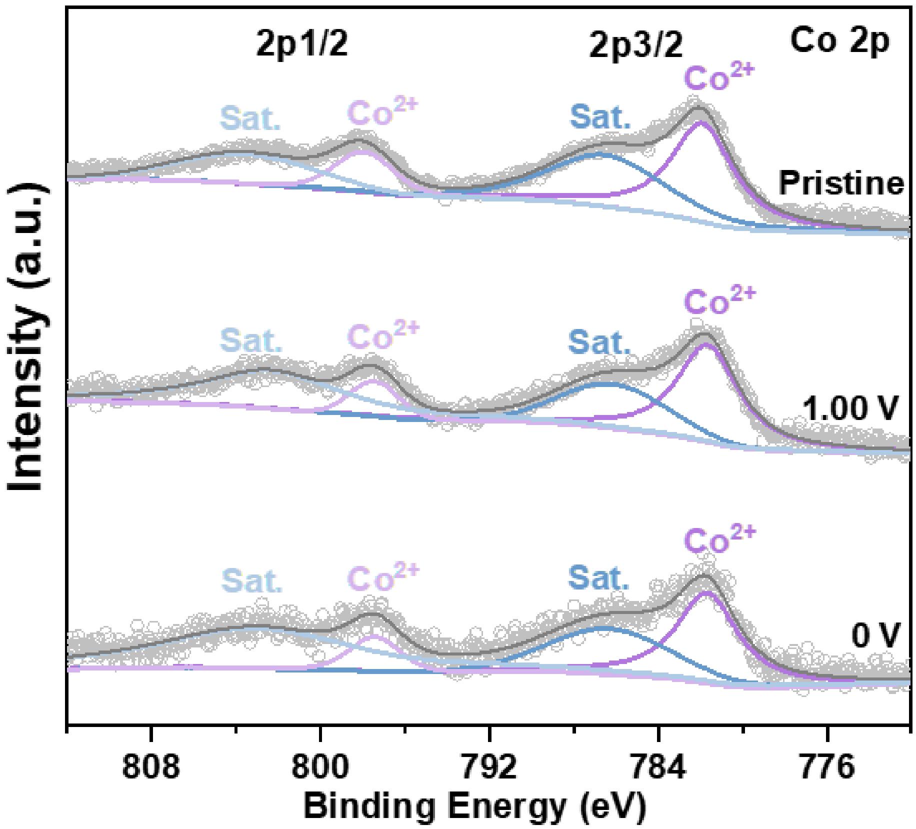


**Figure S25.** Ex situ XPS Co 2p spectra of **PCC-11-Co** at different charged and discharged states.

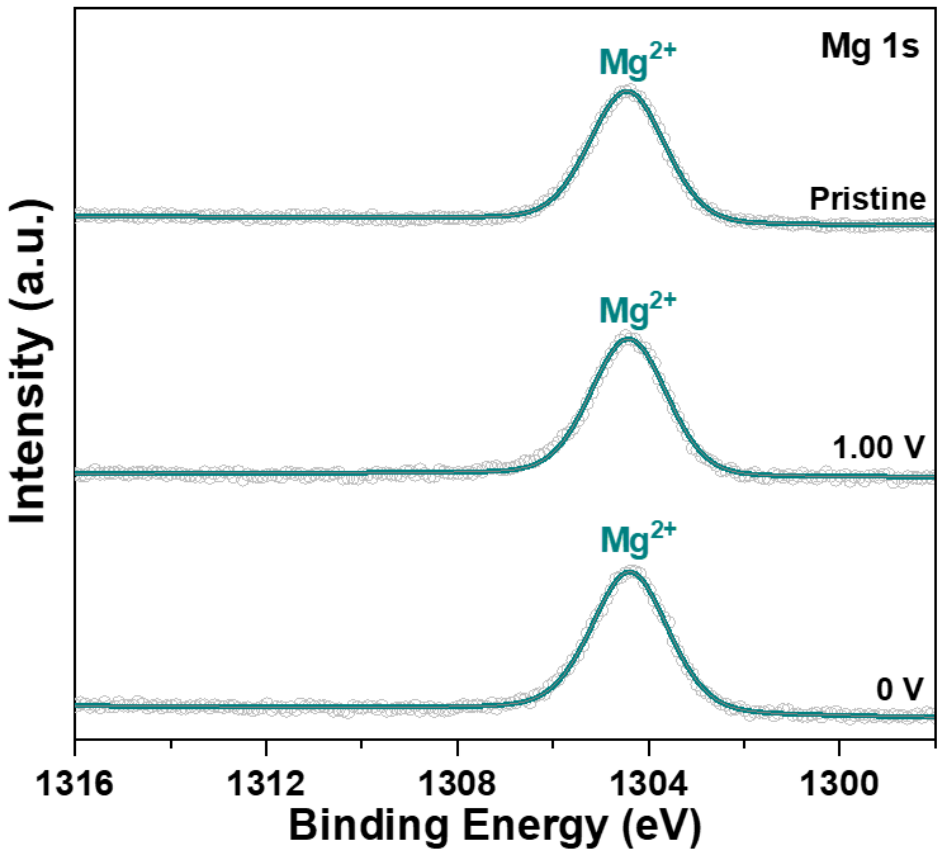


**Figure S26.** Ex situ XPS Mg 1s spectra of **PCC-11-Mg** at different charged and discharged states.

## Section 8. Mechanism of charging and discharging process.

The structure of **PCC-11-Mn** has been optimized, which shows that black, white, red, and yellow spheres represent C, H, O, and S atoms, respectively. The cyan color areas were susceptible to occur the nucleophilic reaction, the electrophilic reaction, and the free radical attack on the corresponding atom in the isosurface of $f^+$, $f^-$, and $f^0$, respectively (Figure 4C). The dual descriptor Δf is more efficient than Fukui functions alone for predicting possible reaction sites. Cyan and purple area can clearly and simultaneously reveal the nucleophilic and electrophilic sites in a molecule.

The Fukui function analysis confirmed that the cavity constructed by the benzoic acids is prone to be attacked by $K^+$, while the vertex ligand is easy to react with the group bearing higher electron cloud density (Figure 4C). Δf behaves in the same way at this point, which indicates that the PCC inner cavity matches the electrophilic ion $K^+$ with a higher binding capacity compared to the semi-open cavity of the vertex ligand.

Based on the experimental results and DFT calculations, we depicted some hypotheses for designing better supercapacitors shortly. Firstly, because the charge carrier should enter the cavity of a porous material to facilitate electron storage, the pore or aperture of the electron binding site should not be small. According to our findings, no smaller than a 2.5 nm aperture is required for KCl aqueous electrolyte system. If expanded to other electrolyte systems, the aperture should be customized to be larger than the size of the cation of electrolyte for allowing its entrance. Secondly, in a hierarchical-porous-material-based electrode, the different pores may contribute to the capacitance in distinct ways. Here, the inner cavity and external cavity contributed to the capacitance in the pseudocapacitive and EDL manner, respectively. Thus, if a material can quantify the contribution of each pore or cavity, the overall capacitance can be finely tuned to achieve the highest value. Finally, the

stabilization of the redox center, including both the metal and the ligand, is the key to maintaining the rate performance in long cycles. The performance loss was frequently observed in pseudocapacitive MOF-based electrodes because the redox active centers hardly maintain high reversibility, thus causing structural decomposition. In the PCC case, the redox active metal center was well protected by the two cavities, endowing its high performance. In a newly designed metal complex, if the redox center is either protected by the surrounding linker or located at the pendant site, rather than the junction site, high recycle stability can be expected.

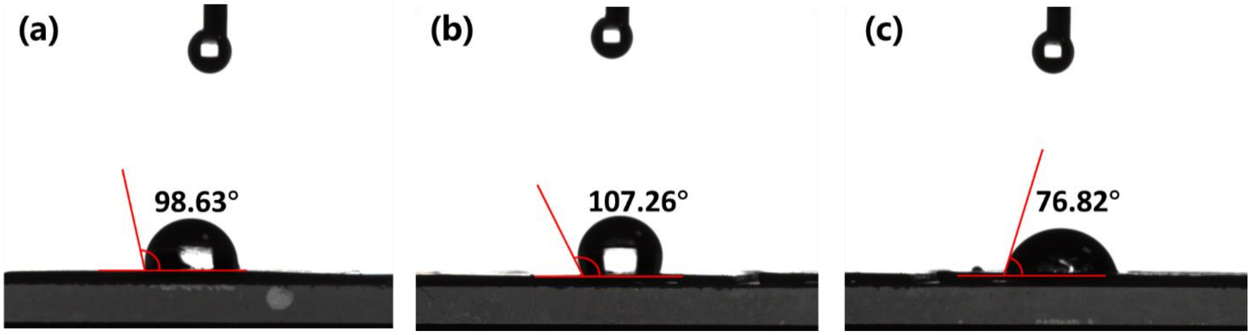


**Figure S27.** Contact angle measurement of pristine **MOF-Mn** electrode (a), pristine (b), and post-activation (c) **PCC-11-Mn** electrode.

The pristine MOF-Mn electrode has a small contact angle (98.63˚) than PCC-11-Mn (107.26˚), which proves the better hydrophilia in the electrolyte. It is surprising that the contact angle of PCC-11-Mn after activation has reduced to 76.82, nowhere near the pristine PCC-11-Mn electrode even less than the pristine **MOF-Mn** electrode.

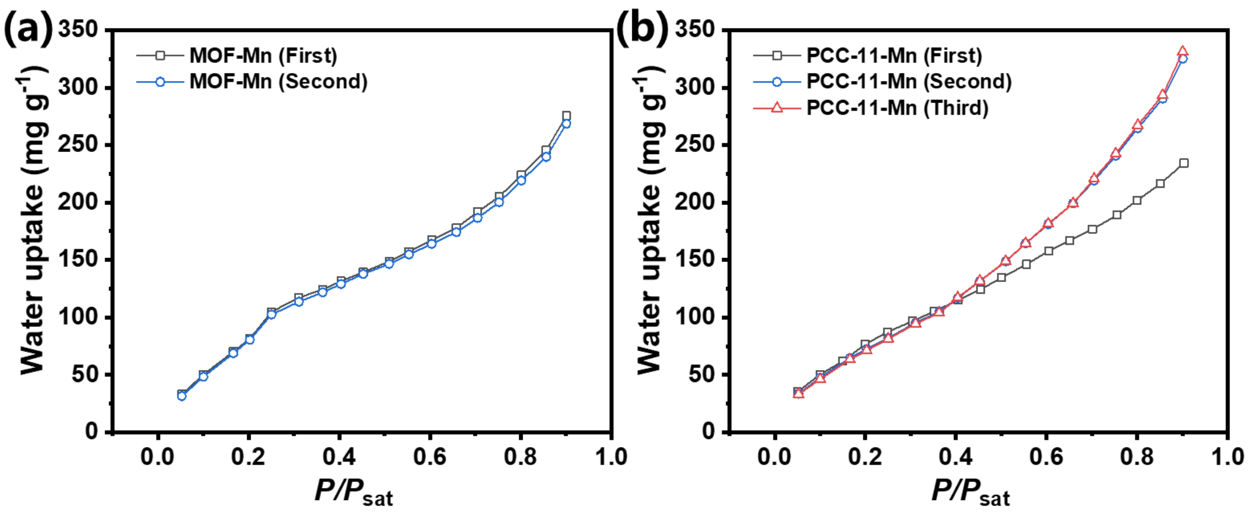


**Figure S28.** Water vapor adsorption isotherms of **MOF-Mn** powder in two cycles (a), and **PCC-11-Mn** powder in three cycles (b).

To further verify the change of hydrophobicity of the two materials, water vapor adsorption tests were performed. The results showed that the adsorption capacity of MOF-Mn did not change significantly in two cycles (276 and 269 mg $g^{-1}$ in the first and second adsorption, respectively), representing a similar adsorption behavior of type III isotherm. The adsorption capacity of PCC-11-Mn (234 mg $g^{-1}$) is slightly lower than MOF-Mn, but it increased obviously in the second (325 mg $g^{-1}$) and third cycle (331 mg $g^{-1}$). Water vapor adsorption cycle tests and contact angle demonstrate the potential for progressive activation of homotopically structured PCC in the aqueous phase.

## Section 9. Flexible electrode of PCC-11-Mn.

The SEM image suggests that the micro-crystals of **PCC-11-Mn** are directly grown on the surface of CC without the assistance of any binder (Figure 5A). This kind of ordered close packing can significantly reduce the intrinsic charge transfer resistance at the electrode/electrolyte interface, enabling effective charge/electron transport on the interface of PCC nanostructures and electrolytes. The energy storage property of the **PCC-11-Mn@CC** electrode was investigated by using 3 M KCl as an aqueous electrolyte in a three-electrode system. CV curves were obtained at scan rates of 5~100 mV $s^{-1}$ (Figure S29a).

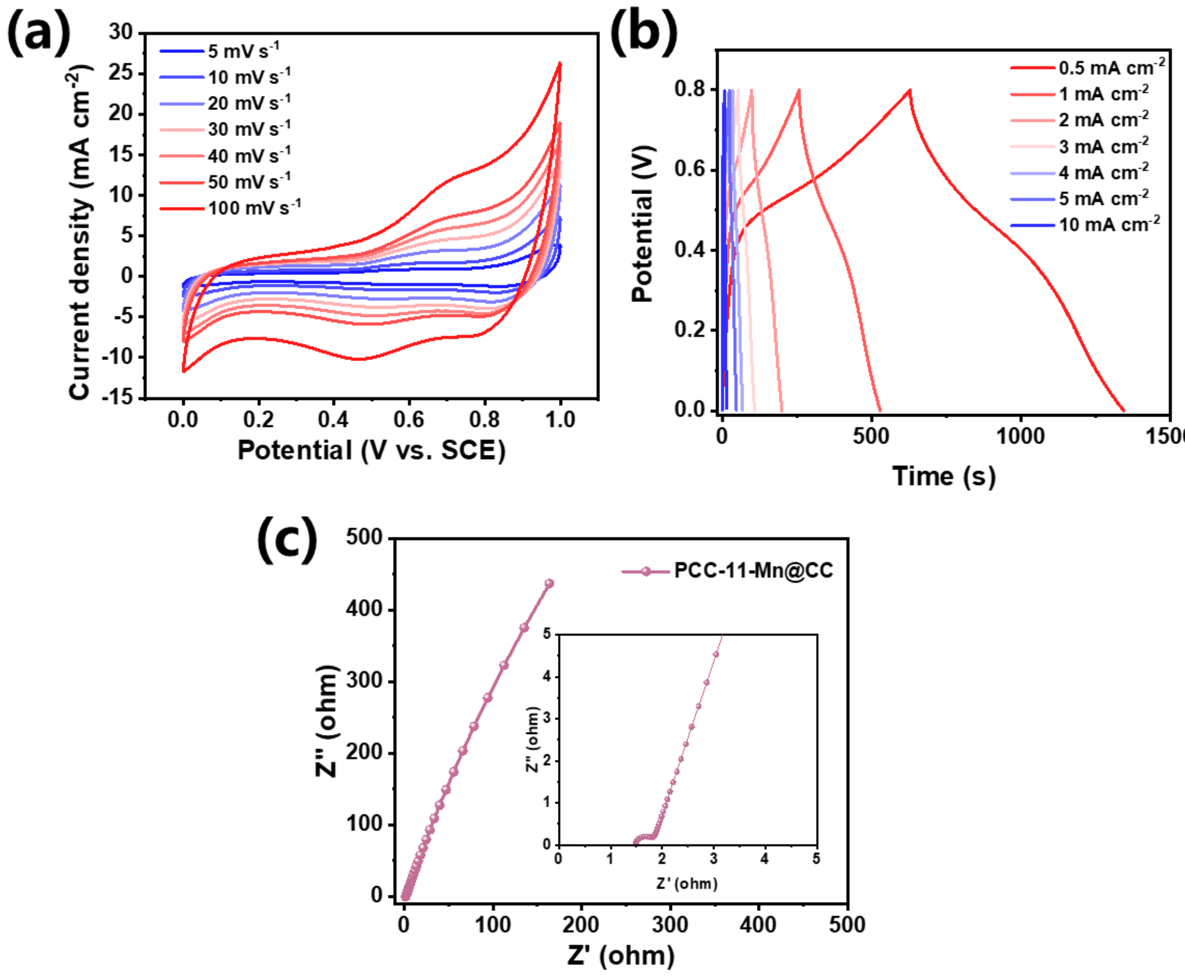


**Figure S29.** (a) CV curves of PCC-11-Mn@CC at different scan rates. (b) GCD curves of PCC-11-Mn@CC at different current densities. (c) Nyquist plot of PCC-11-Mn@CC.

Moreover, it can be observed that the gap between the reduction and oxidation potential peak was significantly reduced by -0.5 V compared to that of the **PCC-11-Mn** electrode, revealing the enhancement of the redox reaction involving fast reaction kinetics and excellent electrochemical reversibility conferred by CC. The specific capacitance of **PCC-11-Mn@CC** was obtained by performing GCD measurements at different current densities from 0.5 mA $cm^{-2}$ to 10 mA $cm^{-2}$ (Figure S29b).

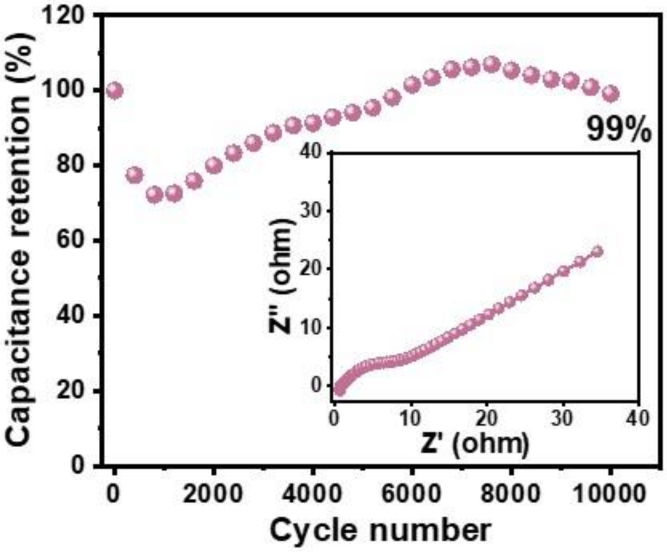


**Figure S30.** Nyquist plots and cycling performance of **PCC-FSC**.

A PCC-based flexible symmetric all-solid-state supercapacitor (**PCC-FSC**) device was fabricated (Figure 5F) and the electronic property was measured. Two identical pieces of **PCC-11-Mn@CC** electrode with an area of 1.5 × 2 $cm^2$ were placed in parallel and KCl/PVA (PVA, polyvinyl alcohol) was used as the gel electrolyte (Figure 5F). The CV curves (Figure 5C) of **PCC-FSC** exhibited approximate rectangular traces (Figure 6D). In addition, the Nyquist plot showed a lower charge transfer resistance (6.18 Ω) in the high-frequency region, which confirms excellent electrical conductivity.

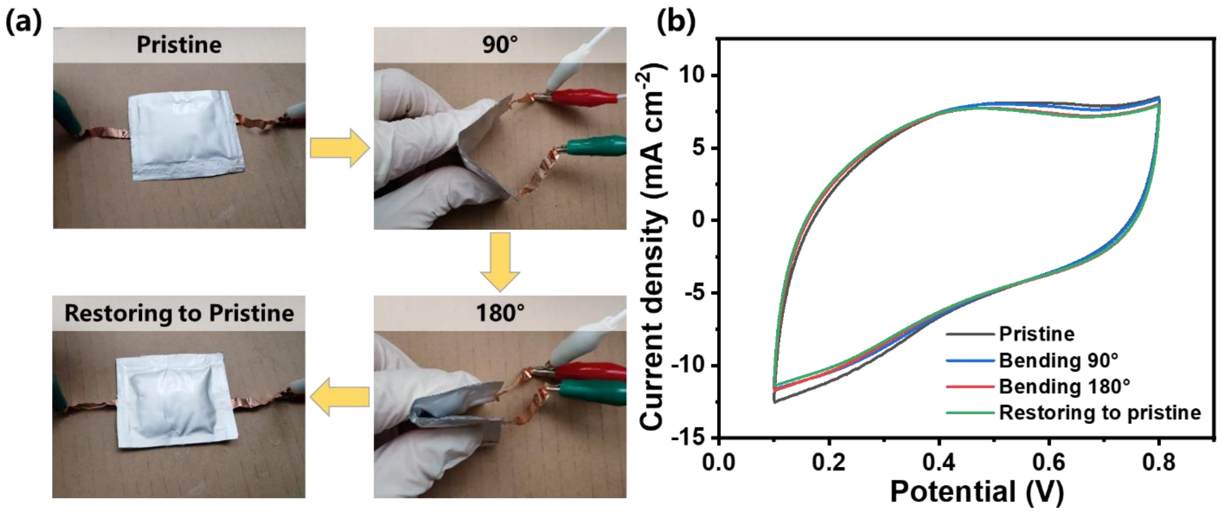


**Figure S31.** (a) Schematic of bending test (a) and CV curves of **PCC-FSC** (a) at 100 mV $s^{-1}$ under different folding angles.

Bending (90 °) or even folding (180 °) the device has no influence on the CV curves, which demonstrates the good flexibility of PCC-FSC. Comparing pristine and restoring to pristine, no significant change in CV curves characterizes good electrochemical cycling stability of PCC-FSC.

## Section 10. Comparison of electrochemical performance.

**Table S10.** Comparison of performance of metal-organic coordinated materials.

| Electrode materials | Electrolyte | Specific capacity | Current density | Retention | Reference |
|---|---|---|---|---|---|
| **PCC-11-Mn** | **3 M KCl** | **243 F $g^{-1}$** | **0.5 A $g^{-1}$** | **115% (10000)** | **This work** |
| THPP-PA-Mn | 1.0 M $Na_2SO_4$ | 90.9 F $g^{-1}$ | 2.5 A $g^{-1}$ | 86 % (3000) | Ref. [12] |
| ZIF-67 | 1.0 M $Na_2SO_4$ | 99.2 F $g^{-1}$ | 0.5 A $g^{-1}$ | 71% (10000) | Ref. [13] |
| Mn-BDC | 1.0 M $Na_2SO_4$ | 177.9 F $g^{-1}$ | 0.5 A $g^{-1}$ | 98% (2000) | Ref. [14] |
| Cu-CAT NWAs | 3 M KCl | 202 F $g^{-1}$ | 0.5 A $g^{-1}$ | 80% (5000) | Ref. [15] |
| CNTs@Mn-MOF | 1.0 M $Na_2SO_4$ | 203.1 F $g^{-1}$ | 1 A $g^{-1}$ | 88% (3000) | Ref. [16] |
| $[Na_2Co(SDCA)(\mu_2\text{-}OH)_2(\mu_2\text{-}H_2O)_2(Azopy)]_n$ | 0.5 M $Na_2SO_4$ | 321.8 F $g^{-1}$ | 4 A $g^{-1}$ | 97% (5000) | Ref. [17] |
| $Ni_2[CuPc(NH)_8]$ | 3 M KCl | 400 F $g^{-1}$ | 0.5 A $g^{-1}$ | 90% (5000) | Ref. [18] |
| $NaZn_2(m_2\text{-}BTC)_2(m_2\text{-}O)_2(Azopy)(H_2O)_2$ | 0.5 M $Na_2SO_4$ | 435.2 F $g^{-1}$ | 1.6 A $g^{-1}$ | 100% (4000) | Ref. [19] |
| Cu-DBC | 1.0 M NaCl | 479 F $g^{-1}$ | 0.2 A $g^{-1}$ | 72% (2000) | Ref. [20] |

| | | | | | |
|---|---|---|---|---|---|
| **PCC-FSC** | **3 M KCl** | **250 mF cm$^{-2}$** | **1 mA cm$^{-2}$** | **99% (10000)** | **This work** |
| $Ni_3(HITP)_2$ | 0.5 M $Na_2SO_4$ | 15.69 mF cm$^{-2}$ | 0.1 mA cm$^{-2}$ | 84% (10000) | Ref. [21] |
| TCNQ@$Cu_3(BTC)_2$ | 0.1 M $Bu_4NPF_6$ | 95.1 mF cm$^{-2}$ | 5 mV s$^{-1}$ | 94% (5000) | Ref. [22] |
| UiO-66/PPY | 3 M KCl | 206 mF cm$^{-2}$ | 5 mV s$^{-1}$ | 89% (10000) | Ref. [23] |
| Cu-CAT-NWAs/PPy | 3 M KCl | 252.1 mF cm$^{-2}$ | 1.25 mA cm$^{-2}$ | 90% (8000) | Ref. [24] |

## Reference

[1] Stephens, P. J.; Devlin, F. J.; Chabalowski, C. F.; Frisch, M. J. Ab Initio Calculation of Vibrational Absorption and Circular Dichroism Spectra Using Density Functional Force Fields. *J. Phys. Chem.* **1994**, *98*, 45.

[2] Jiang, X.; Jefferson, W. A.; Song, D.; Cheng, H.; Li, F.; Qiang, Z.; Zhang, A.; Liu, H.; Qu, J. Regioselective oxidation of tetracycline by permanganate through alternating susceptible moiety and increasing electron donating ability. *J. Environ. Sci.-China* **2020**, *87*, 281–288.

[3] Lu, T.; Chen, F. Multiwfn: a multifunctional wavefunction analyzer. *J. Comput. Chem.* **2012**, *33* (5), 580–592.

[4] Humphrey, W.; Dalke, A.; Schulten, K. VMD: Visual Molecular Dynamics. *J. Mol. Graphics* **1996**, *14*, 33–38.

[5] Marri, S. R.; Chauhan, N.; Tiwari, R. K.; Kumar, J.; Behera, J. N. Two novel 3D-MOFs (Ca-TATB and Co-HKUST): Synthesis, structure and characterization. *Inorg. Chim. Acta* **2018**, *478*, 8–14.

[6] Katagiri, H.; Iki, N.; Hattori, T.; Kabuto, C.; Miyano, S. Calix[4]arenes Comprised of Aniline Units. *J. Am. Chem. Soc.* **2001**, *123*, 779–780.

[7] Du, S.; Yu, T.-Q.; Liao, W.; Hu, C. Structure modeling, synthesis and X-ray diffraction determination of an extra-large calixarene-based coordination cage and its application in drug delivery. *Dalton Trans.* **2015**, *44*, 14394–14402.

[8] Dai, F. R.; Wang, Z. Modular assembly of metal-organic supercontainers incorporating sulfonylcalixarenes. *J. Am. Chem. Soc.* **2012**, *134* (19), 80024–8005.

[9] Zhang, Y.-B.; Furukawa, H.; Ko, N.; Nie, W.; Park, H. J.; Okajima, S.; Cordova, K. E.; Deng, H.; Kim, J.; Yaghi, O. M. Introduction of functionality, selection of topology, and enhancement of gas adsorption in multivariate metal-organic framework-177. *J. Am. Chem. Soc.* **2015**, *137* (7), 2641–2650.

[10] Lamouchi, M.; Jeanneau, E.; Pillonnet, A.; Brioude, A.; Martini, M.; Stéphan, O.; Meganem, F.; Novitchi, G.; Luneaua, D.; Desroches, C. Tetranuclear manganese(II) complexes of sulfonylcalix[4]arene macrocycles: synthesis, structure, spectroscopic and magnetic properties. *Dalton Trans.* **2012**, *41*, 2707–2713.

[11] Ma, S.; Zhou, H.-C. A Metal-Organic Framework with Entatic Metal Centers Exhibiting High Gas Adsorption Affinity. *J. Am. Chem. Soc.* **2006**, *128*, 11734–11735.

[12] Cheng, Z.; Qiu, Y.; Tan, G.; Chang, X.; Luo, Q.; Cui, L. Synthesis of a Novel Mn(II)-porphyrins polycondensation polymer and its application as pseudo-capacitor electrode material. *J. Organomet. Chem.* **2019**, *900*, 120940.

[13] Xu, X.; Tang, J.; Qian, H.; Hou, S.; Bando, Y.; Hossain, M. S. A.; Pan, L.; Yamauchi, Y. Three-Dimensional Networked Metal-Organic Frameworks with Conductive Polypyrrole Tubes for Flexible Supercapacitors. *ACS Appl. Mater. Inter.* **2017**, *9* (44), 38737–38744.

[14] Shashank Sundriyal, S. M. Akash Deep, Study of Manganese-1,4-Benzenedicarboxylate Metal Organic Framework Electrodes Based Solid State Symmetrical Supercapacitor. *Energy Procedia* **2019**, *158*, 5817–5824.

[15] Zhang, P.; Wang, M.; Liu, Y.; Yang, S.; Wang, F.; Li, Y.; Chen, G.; Li, Z.; Wang, G.; Zhu, M.; Dong, R.; Yu, M.; Schmidt, O. G.; Feng, X. Dual-Redox-Sites Enable Two-Dimensional Conjugated Metal-Organic Frameworks with Large Pseudocapacitance and Wide Potential Window. *J. Am. Chem. Soc.* **2021**, *143* (27), 10168–10176.

[16] Zhang, Y.; Lin, B.; Sun, Y.; Zhang, X.; Yang, H.; Wang, J. Carbon nanotubes@metal–organic frameworks as Mn-based symmetrical supercapacitor electrodes for enhanced charge storage. *RSC Adv.* **2015**, *5* (72), 58100–58106.

[17] Rajak, R.; Saraf, M.; Mobin, S. M. Mixed-Ligand Architected Unique Topological Heterometallic Sodium/Cobalt-Based Metal-Organic Framework for High-Performance Supercapacitors. *Inorg. Chem.* **2020**, *59* (3), 1642–1652.

[18] Li, W.-H.; Ding, K.; Tian, H.-R.; Yao, M.-S.; Nath, B.; Deng, W.-H.; Wang, Y.; Xu, G. Conductive Metal-Organic Framework Nanowire Array Electrodes for High-Performance Solid-State Supercapacitors. *Adv. Funct. Mater.* **2017**, *27* (27), 1702067.

[19] Rajak, R.; Saraf, M.; Mobin, S. M. Robust heterostructures of a bimetallic sodium–zinc metal–organic framework and reduced graphene oxide for high-performance supercapacitors. *J. Mater. Chem. A* **2019**, *7* (4), 1725–1736.

[20] Liu, J.; Zhou, Y.; Xie, Z.; Li, Y.; Liu, Y.; Sun, J.; Ma, Y.; Terasaki, O.; Chen, L. Conjugated Copper-Catecholate Framework Electrodes for Efficient Energy Storage. *Angew. Chem. Int. Ed.* **2020**, *59* (3), 1081–1086.

[21] Nguyen, D. K.; Schepisi, I. M.; Amir, F. Z. Extraordinary cycling stability of $Ni_3(HITP)_2$ supercapacitors fabricated by electrophoretic deposition: Cycling at 100,000 cycles. *Chem. Eng. J.* **2019**, *378*, 122150.

[22] He, Y.; Yang, S.; Fu, Y.; Wang, F.; Ma, J.; Wang, G.; Chen, G.; Wang, M.; Dong, R.; Zhang, P.; Feng, X. Electronic Doping of Metal-Organic Frameworks for High-Performance Flexible Micro-Supercapacitors. *Small Struct.* **2020**, *2,* 2000095.

[23] Hou, R.; Miao, M.; Wang, Q.; Yue, T.; Liu, H.; Park, H. S.; Qi, K.; Xia, B. Y. Integrated Conductive Hybrid Architecture of Metal–Organic Framework Nanowire Array on Polypyrrole Membrane for All-Solid-State Flexible Supercapacitors. *Adv. Energy Mater.* **2019**, *10* (1), 1901892.

[24] Qi, K.; Hou, R.; Zaman, S.; Qiu, Y.; Xia, B. Y.; Duan, H. Construction of Metal-Organic Framework/Conductive Polymer Hybrid for All-Solid-State Fabric Supercapacitor. *ACS Appl. Mater. Inter.* **2018**, *10* (21), 18021–18028.
