# Supplementary material for: Bridging the Gap between Charge Storage Site and Transportation Pathway in Molecular-Cage-Based Flexible Electrodes
Source: ACS Cent Sci. 2023 Apr 5;9(4):805–15. doi: 10.1021/acscentsci.3c00027 (PMC10141610; doi:10.1021/acscentsci.3c00027)

checkCIF/PLATON report

You have not supplied any structure factors. As a result the full set of tests cannot be run.

THIS REPORT IS FOR GUIDANCE ONLY. IF USED AS PART OF A REVIEW PROCEDURE FOR PUBLICATION, IT SHOULD NOT REPLACE THE EXPERTISE OF AN EXPERIENCED CRYSTALLOGRAPHIC REFEREE.

No syntax errors found. CIF dictionary Interpreting this report

Datablock: lkkmn24

Bond precision: C-C = 0.0107 A Wavelength=0.71073

Cell:	a=26.662(7)	b=26.662(7)	c=44.644(16)
	alpha=90	beta=90	gamma=90
Temperature:	273 K		

	Calculated	Reported
Volume	31736(20)	31736(20)
Space group	I 4/m	I 4/m
Hall group	-I 4	-I 4
Moiety formula	C312 H288 Mn24 O126 S24 [+ solvent]	C312 H288 Mn24 O126 S24
Sum formula	C312 H288 Mn24 O126 S24 [+ solvent]	C312 H288 Mn24 O126 S24
Mr	8141.43	8141.41
Dx,g cm-3	0.852	0.852
Z	2	2
Mu (mm-1)	0.587	0.587
F000	8304.0	8304.0
F000'	8329.93	
h,k,lmax	31,31,53	31,31,53
Nref	14194	14109
Tmin,Tmax	0.759,0.814	
Tmin'	0.759	

Correction method= Not given

Data completeness= 0.994 Theta(max)= 25.000

R(reflections)= 0.1073(12215) wR2(reflections)= 0.4069(14109)

S = 1.137 Npar= 601

The following ALERTS were generated. Each ALERT has the format
test-name_ALERT_alert-type_alert-level.
Click on the hyperlinks for more details of the test.

Alert level B

```
RINTA01_ALERT_3_B  The value of Rint is greater than 0.18
            Rint given   0.207
PLAT020_ALERT_3_B The Value of Rint is Greater Than 0.12 .........      0.207 Report
PLAT084_ALERT_3_B High wR2 Value (i.e. > 0.25) ...................       0.41 Report
PLAT213_ALERT_2_B Atom C15              has ADP max/min Ratio .....       4.2 prolat
PLAT220_ALERT_2_B NonSolvent   Resd 1  C   Ueq(max)/Ueq(min) Range        8.4 Ratio
PLAT242_ALERT_2_B Low    'MainMol' Ueq as Compared to Neighbors of       C01T Check
PLAT412_ALERT_2_B Short Intra XH3 .. XHn     H15C     ..H41A     .       1.75 Ang.
                                                      x,y,z  =      1_555 Check
```

Alert level C

```
PLAT082_ALERT_2_C High R1 Value ..................................       0.11 Report
PLAT213_ALERT_2_C Atom O009             has ADP max/min Ratio .....       3.3 prolat
PLAT213_ALERT_2_C Atom C01T             has ADP max/min Ratio .....       3.1 prolat
PLAT215_ALERT_3_C Disordered C19A       has ADP max/min Ratio .....       3.1 Note
PLAT215_ALERT_3_C Disordered C61A       has ADP max/min Ratio .....       3.7 Note
PLAT222_ALERT_3_C NonSolvent Resd 1  H   Uiso(max)/Uiso(min) Range        9.2 Ratio
PLAT241_ALERT_2_C High   'MainMol' Ueq as Compared to Neighbors of       C01E Check
PLAT241_ALERT_2_C High   'MainMol' Ueq as Compared to Neighbors of       C01M Check
PLAT250_ALERT_2_C Large U3/U1 Ratio for Average U(i,j) Tensor ....        2.3 Note
PLAT341_ALERT_3_C Low Bond Precision on  C-C Bonds ...............    0.01067 Ang.
PLAT360_ALERT_2_C Short  C(sp3)-C(sp3) Bond  C15      - C01T     .       1.41 Ang.
PLAT412_ALERT_2_C Short Intra XH3 .. XHn     H018     ..H01M     .       1.89 Ang.
                                                      x,y,z  =      1_555 Check
PLAT412_ALERT_2_C Short Intra XH3 .. XHn     H01E     ..H01I     .       1.89 Ang.
                                                      x,y,z  =      1_555 Check
```

Alert level G

```
PLAT002_ALERT_2_G Number of Distance or Angle Restraints on AtSite         15 Note
PLAT003_ALERT_2_G Number of Uiso or Uij Restrained non-H Atoms ...         15 Report
PLAT072_ALERT_2_G SHELXL First  Parameter in WGHT  Unusually Large       0.35 Report
PLAT172_ALERT_4_G The CIF-Embedded .res File Contains DFIX Records         20 Report
PLAT187_ALERT_4_G The CIF-Embedded .res File Contains RIGU Records          1 Report
PLAT199_ALERT_1_G Reported _cell_measurement_temperature ..... (K)        273 Check
PLAT200_ALERT_1_G Reported   _diffrn_ambient_temperature ..... (K)        273 Check
PLAT300_ALERT_4_G Atom Site Occupancy of C19A       Constrained at        0.5 Check
PLAT300_ALERT_4_G Atom Site Occupancy of C19B       Constrained at        0.5 Check
PLAT300_ALERT_4_G Atom Site Occupancy of C36A       Constrained at        0.5 Check
PLAT300_ALERT_4_G Atom Site Occupancy of C36B       Constrained at        0.5 Check
PLAT300_ALERT_4_G Atom Site Occupancy of C61A       Constrained at        0.5 Check
PLAT300_ALERT_4_G Atom Site Occupancy of C61B       Constrained at        0.5 Check
PLAT300_ALERT_4_G Atom Site Occupancy of H19A       Constrained at        0.5 Check
PLAT300_ALERT_4_G Atom Site Occupancy of H19B       Constrained at        0.5 Check
PLAT300_ALERT_4_G Atom Site Occupancy of H19C       Constrained at        0.5 Check
PLAT300_ALERT_4_G Atom Site Occupancy of H19D       Constrained at        0.5 Check
PLAT300_ALERT_4_G Atom Site Occupancy of H19E       Constrained at        0.5 Check
PLAT300_ALERT_4_G Atom Site Occupancy of H19F       Constrained at        0.5 Check
```

```
PLAT300_ALERT_4_G Atom Site Occupancy of H36A       Constrained at        0.5 Check
PLAT300_ALERT_4_G Atom Site Occupancy of H36B       Constrained at        0.5 Check
PLAT300_ALERT_4_G Atom Site Occupancy of H36C       Constrained at        0.5 Check
PLAT300_ALERT_4_G Atom Site Occupancy of H36D       Constrained at        0.5 Check
PLAT300_ALERT_4_G Atom Site Occupancy of H36E       Constrained at        0.5 Check
PLAT300_ALERT_4_G Atom Site Occupancy of H36F       Constrained at        0.5 Check
PLAT300_ALERT_4_G Atom Site Occupancy of H61A       Constrained at        0.5 Check
PLAT300_ALERT_4_G Atom Site Occupancy of H61B       Constrained at        0.5 Check
PLAT300_ALERT_4_G Atom Site Occupancy of H61C       Constrained at        0.5 Check
PLAT300_ALERT_4_G Atom Site Occupancy of H61D       Constrained at        0.5 Check
PLAT300_ALERT_4_G Atom Site Occupancy of H61E       Constrained at        0.5 Check
PLAT300_ALERT_4_G Atom Site Occupancy of H61F       Constrained at        0.5 Check
PLAT301_ALERT_3_G Main Residue  Disorder ..............(Resd  1  )         5% Note
PLAT412_ALERT_2_G Short Intra XH3 .. XHn     H01G     ..H61C     .       1.98 Ang.
                                                      x,y,z  =      1_555 Check
PLAT412_ALERT_2_G Short Intra XH3 .. XHn     H01G     ..H61D     .       1.81 Ang.
                                                      x,y,z  =      1_555 Check
PLAT412_ALERT_2_G Short Intra XH3 .. XHn     H01H     ..H36C     .       2.09 Ang.
                                                      x,y,z  =      1_555 Check
PLAT432_ALERT_2_G Short Inter X...Y Contact  C19B     ..C01V     .       3.20 Ang.
                                          3/2-y,1/2+x,1/2-z  =     16_655 Check
PLAT606_ALERT_4_G Solvent Accessible VOID(S) in Structure ........          ! Info
PLAT720_ALERT_4_G Number of Unusual/Non-Standard Labels ..........         78 Note
PLAT764_ALERT_4_G Overcomplete CIF Bond List Detected (Rep/Expd) .       1.20 Ratio
PLAT793_ALERT_4_G Model has Chirality at S07        (Centro SPGR)           R Verify
PLAT794_ALERT_5_G Tentative Bond Valency for Mn01      (II)      .       2.12 Info
PLAT794_ALERT_5_G Tentative Bond Valency for Mn02      (II)      .       2.12 Info
PLAT794_ALERT_5_G Tentative Bond Valency for Mn03      (II)      .       2.14 Info
PLAT794_ALERT_5_G Tentative Bond Valency for Mn04      (II)      .       2.13 Info
PLAT860_ALERT_3_G Number of Least-Squares Restraints ............        141 Note
PLAT967_ALERT_5_G Note: Two-Theta Cutoff Value in Embedded .res ..       50.0 Degree
```

---

0 **ALERT level A** = Most likely a serious problem - resolve or explain
7 **ALERT level B** = A potentially serious problem, consider carefully
13 **ALERT level C** = Check. Ensure it is not caused by an omission or oversight
46 **ALERT level G** = General information/check it is not something unexpected

2 ALERT type 1 CIF construction/syntax error, inconsistent or missing data
20 ALERT type 2 Indicator that the structure model may be wrong or deficient
9 ALERT type 3 Indicator that the structure quality may be low
30 ALERT type 4 Improvement, methodology, query or suggestion
5 ALERT type 5 Informative message, check

It is advisable to attempt to resolve as many as possible of the alerts in all categories. Often the minor alerts point to easily fixed oversights, errors and omissions in your CIF or refinement strategy, so attention to these fine details can be worthwhile. In order to resolve some of the more serious problems it may be necessary to carry out additional measurements or structure refinements. However, the purpose of your study may justify the reported deviations and the more serious of these should normally be commented upon in the discussion or experimental section of a paper or in the "special_details" fields of the CIF. checkCIF was carefully designed to identify outliers and unusual parameters, but every test has its limitations and alerts that are not important in a particular case may appear. Conversely, the absence of alerts does not guarantee there are no aspects of the results needing attention. It is up to the individual to critically assess their own results and, if necessary, seek expert advice.

Publication of your CIF in IUCr journals

A basic structural check has been run on your CIF. These basic checks will be run on all CIFs submitted for publication in IUCr journals (*Acta Crystallographica*, *Journal of Applied Crystallography*, *Journal of Synchrotron Radiation*); however, if you intend to submit to *Acta Crystallographica Section C* or *E* or *IUCrData*, you should make sure that full publication checks are run on the final version of your CIF prior to submission.

Publication of your CIF in other journals

Please refer to the *Notes for Authors* of the relevant journal for any special instructions relating to CIF submission.

PLATON version of 12/09/2022; check.def file version of 09/08/2022

Datablock lkkmn24 - ellipsoid plot

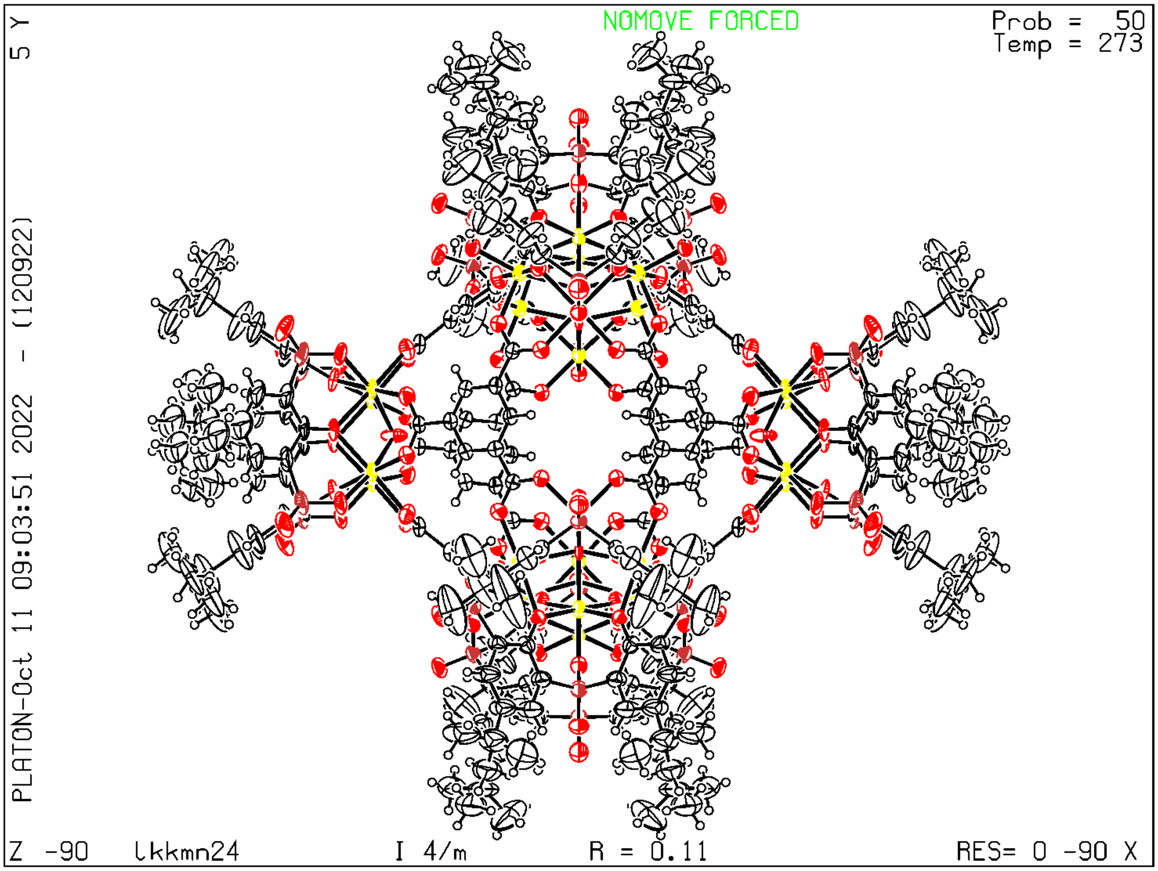

Supplement: Supplementary file 3 — oc3c00027_si_003.pdf [file oc3c00027_si_003.pdf]
