# Supplementary material for: Bridging the Gap between Charge Storage Site and Transportation Pathway in Molecular-Cage-Based Flexible Electrodes
Source: ACS Cent Sci. 2023 Apr 5;9(4):805–15. doi: 10.1021/acscentsci.3c00027 (PMC10141610; doi:10.1021/acscentsci.3c00027)

# checkCIF/PLATON report

You have not supplied any structure factors. As a result the full set of tests cannot be run.

THIS REPORT IS FOR GUIDANCE ONLY. IF USED AS PART OF A REVIEW PROCEDURE FOR PUBLICATION, IT SHOULD NOT REPLACE THE EXPERTISE OF AN EXPERIENCED CRYSTALLOGRAPHIC REFEREE.

No syntax errors found. CIF dictionary Interpreting this report

# Datablock: co-8

---

Bond precision: C-C = 0.0126 A Wavelength=0.71073

| | | | |
|---|---|---|---|
| Cell: | a=33.0843(5) | b=33.0843(5) | c=54.196(2) |
| | alpha=90 | beta=90 | gamma=90 |
| Temperature: | 100 K | | |

| | Calculated | Reported |
|---|---|---|
| Volume | 59321(3) | 59321(3) |
| Space group | I 4/m | I 4/m |
| Hall group | -I 4 | -I 4 |
| Moiety formula | C432 H360 Co24 N24 O126 S24 [+ solvent] | C432 H360 Co24 N24 O126 S24 |
| Sum formula | C432 H360 Co24 N24 O126 S24 [+ solvent] | C432 H360 Co24 N24 O126 S24 |
| Mr | 10087.22 | 10087.18 |
| Dx,g cm-3 | 0.565 | 0.565 |
| Z | 2 | 2 |
| Mu (mm-1) | 0.400 | 0.400 |
| F000 | 10320.0 | 10320.0 |
| F000' | 10347.04 | |
| h,k,lmax | 39,39,64 | 39,39,64 |
| Nref | 26453 | 25225 |
| Tmin,Tmax | 0.878,0.912 | 0.801,1.000 |
| Tmin' | 0.859 | |

Correction method= # Reported T Limits: Tmin=0.801 Tmax=1.000
AbsCorr = MULTI-SCAN

Data completeness= 0.954 Theta(max)= 24.999

R(reflections)= 0.1212( 12963)    wR2(reflections)= 0.3765( 25225)

S = 1.033    Npar= 787

---

The following ALERTS were generated. Each ALERT has the format
**test-name_ALERT_alert-type_alert-level.**
Click on the hyperlinks for more details of the test.

---

### Alert level B

```
PLAT029_ALERT_3_B _diffrn_measured_fraction_theta_full value Low .      0.954 Why?
PLAT084_ALERT_3_B High wR2 Value (i.e. > 0.25) ...................       0.38 Report
PLAT196_ALERT_1_B No TEMP record and _measurement_temperature .NE.        293 Degree
```

---

### Alert level C

```
PLAT082_ALERT_2_C High R1 Value ..................................       0.12 Report
PLAT213_ALERT_2_C Atom O4              has ADP max/min Ratio .....        3.1 oblate
PLAT213_ALERT_2_C Atom C49             has ADP max/min Ratio .....        3.2 prolat
PLAT213_ALERT_2_C Atom C60             has ADP max/min Ratio .....        3.4 prolat
PLAT213_ALERT_2_C Atom C71             has ADP max/min Ratio .....        3.1 prolat
PLAT213_ALERT_2_C Atom C79             has ADP max/min Ratio .....        3.5 prolat
PLAT213_ALERT_2_C Atom C88             has ADP max/min Ratio .....        3.6 prolat
PLAT220_ALERT_2_C NonSolvent   Resd 1  C   Ueq(max)/Ueq(min) Range        3.6 Ratio
PLAT241_ALERT_2_C High   'MainMol' Ueq as Compared to Neighbors of         O1 Check
PLAT241_ALERT_2_C High   'MainMol' Ueq as Compared to Neighbors of        N46 Check
PLAT241_ALERT_2_C High   'MainMol' Ueq as Compared to Neighbors of         C1 Check
PLAT241_ALERT_2_C High   'MainMol' Ueq as Compared to Neighbors of        C10 Check
PLAT241_ALERT_2_C High   'MainMol' Ueq as Compared to Neighbors of        C49 Check
PLAT241_ALERT_2_C High   'MainMol' Ueq as Compared to Neighbors of        C56 Check
PLAT241_ALERT_2_C High   'MainMol' Ueq as Compared to Neighbors of        C60 Check
PLAT241_ALERT_2_C High   'MainMol' Ueq as Compared to Neighbors of        C71 Check
PLAT241_ALERT_2_C High   'MainMol' Ueq as Compared to Neighbors of        C75 Check
PLAT241_ALERT_2_C High   'MainMol' Ueq as Compared to Neighbors of        C88 Check
PLAT242_ALERT_2_C Low    'MainMol' Ueq as Compared to Neighbors of       C3AA Check
PLAT242_ALERT_2_C Low    'MainMol' Ueq as Compared to Neighbors of       C2AA Check
PLAT242_ALERT_2_C Low    'MainMol' Ueq as Compared to Neighbors of       C5AA Check
PLAT242_ALERT_2_C Low    'MainMol' Ueq as Compared to Neighbors of        C14 Check
PLAT242_ALERT_2_C Low    'MainMol' Ueq as Compared to Neighbors of        C42 Check
PLAT242_ALERT_2_C Low    'MainMol' Ueq as Compared to Neighbors of        C50 Check
PLAT242_ALERT_2_C Low    'MainMol' Ueq as Compared to Neighbors of        C78 Check
PLAT242_ALERT_2_C Low    'MainMol' Ueq as Compared to Neighbors of       C194 Check
PLAT341_ALERT_3_C Low Bond Precision on  C-C Bonds ...............    0.01258 Ang.
PLAT731_ALERT_1_C Bond    Calc   1.365(14), Rep     1.37(6) ......          4 su-Rat
              C79      -C78             1_555  12_655 ........     #  137 Check
```

---

### Alert level G

```
PLAT002_ALERT_2_G Number of Distance or Angle Restraints on AtSite         32 Note
PLAT003_ALERT_2_G Number of Uiso or Uij Restrained non-H Atoms ...         21 Report
PLAT012_ALERT_1_G No       _shelx_res_checksum Found in CIF ......     Please Check
PLAT014_ALERT_1_G No       _shelx_fab_checksum Found in CIF ......     Please Check
PLAT072_ALERT_2_G SHELXL First  Parameter in WGHT  Unusually Large       0.19 Report
PLAT083_ALERT_2_G SHELXL Second Parameter in WGHT  Unusually Large     203.15 Why ?
PLAT172_ALERT_4_G The CIF-Embedded .res File Contains DFIX Records         44 Report
```

```
PLAT186_ALERT_4_G The CIF-Embedded .res File Contains ISOR Records          3 Report
PLAT187_ALERT_4_G The CIF-Embedded .res File Contains RIGU Records          3 Report
PLAT300_ALERT_4_G Atom Site Occupancy of C0AA       Constrained at        0.5 Check
PLAT300_ALERT_4_G Atom Site Occupancy of C6AA       Constrained at        0.5 Check
PLAT300_ALERT_4_G Atom Site Occupancy of C28        Constrained at        0.5 Check
PLAT300_ALERT_4_G Atom Site Occupancy of C31        Constrained at        0.5 Check
PLAT300_ALERT_4_G Atom Site Occupancy of C35        Constrained at        0.5 Check
PLAT300_ALERT_4_G Atom Site Occupancy of C36        Constrained at        0.5 Check
PLAT300_ALERT_4_G Atom Site Occupancy of C39        Constrained at        0.5 Check
PLAT300_ALERT_4_G Atom Site Occupancy of C44        Constrained at        0.5 Check
PLAT300_ALERT_4_G Atom Site Occupancy of C70        Constrained at        0.5 Check
PLAT300_ALERT_4_G Atom Site Occupancy of C73        Constrained at        0.5 Check
PLAT300_ALERT_4_G Atom Site Occupancy of C81        Constrained at        0.5 Check
PLAT300_ALERT_4_G Atom Site Occupancy of C86        Constrained at        0.5 Check
PLAT300_ALERT_4_G Atom Site Occupancy of C94        Constrained at        0.5 Check
PLAT300_ALERT_4_G Atom Site Occupancy of C98        Constrained at        0.5 Check
PLAT300_ALERT_4_G Atom Site Occupancy of C135       Constrained at        0.5 Check
PLAT300_ALERT_4_G Atom Site Occupancy of C148       Constrained at        0.5 Check
PLAT300_ALERT_4_G Atom Site Occupancy of C192       Constrained at        0.5 Check
PLAT300_ALERT_4_G Atom Site Occupancy of C241       Constrained at        0.5 Check
PLAT300_ALERT_4_G Atom Site Occupancy of H0AA       Constrained at        0.5 Check
PLAT300_ALERT_4_G Atom Site Occupancy of H0AB       Constrained at        0.5 Check
PLAT300_ALERT_4_G Atom Site Occupancy of H0AC       Constrained at        0.5 Check
PLAT300_ALERT_4_G Atom Site Occupancy of H6AA       Constrained at        0.5 Check
PLAT300_ALERT_4_G Atom Site Occupancy of H6AB       Constrained at        0.5 Check
PLAT300_ALERT_4_G Atom Site Occupancy of H6AC       Constrained at        0.5 Check
PLAT300_ALERT_4_G Atom Site Occupancy of H13A       Constrained at        0.5 Check
PLAT300_ALERT_4_G Atom Site Occupancy of H13B       Constrained at        0.5 Check
PLAT300_ALERT_4_G Atom Site Occupancy of H13C       Constrained at        0.5 Check
PLAT300_ALERT_4_G Atom Site Occupancy of H14A       Constrained at        0.5 Check
PLAT300_ALERT_4_G Atom Site Occupancy of H14B       Constrained at        0.5 Check
PLAT300_ALERT_4_G Atom Site Occupancy of H14C       Constrained at        0.5 Check
PLAT300_ALERT_4_G Atom Site Occupancy of H19A       Constrained at        0.5 Check
PLAT300_ALERT_4_G Atom Site Occupancy of H19B       Constrained at        0.5 Check
PLAT300_ALERT_4_G Atom Site Occupancy of H19C       Constrained at        0.5 Check
PLAT300_ALERT_4_G Atom Site Occupancy of H24A       Constrained at        0.5 Check
PLAT300_ALERT_4_G Atom Site Occupancy of H24B       Constrained at        0.5 Check
PLAT300_ALERT_4_G Atom Site Occupancy of H24C       Constrained at        0.5 Check
PLAT300_ALERT_4_G Atom Site Occupancy of H28A       Constrained at        0.5 Check
PLAT300_ALERT_4_G Atom Site Occupancy of H28B       Constrained at        0.5 Check
PLAT300_ALERT_4_G Atom Site Occupancy of H28C       Constrained at        0.5 Check
PLAT300_ALERT_4_G Atom Site Occupancy of H31A       Constrained at        0.5 Check
PLAT300_ALERT_4_G Atom Site Occupancy of H31B       Constrained at        0.5 Check
PLAT300_ALERT_4_G Atom Site Occupancy of H31C       Constrained at        0.5 Check
PLAT300_ALERT_4_G Atom Site Occupancy of H35A       Constrained at        0.5 Check
PLAT300_ALERT_4_G Atom Site Occupancy of H35B       Constrained at        0.5 Check
PLAT300_ALERT_4_G Atom Site Occupancy of H35C       Constrained at        0.5 Check
PLAT300_ALERT_4_G Atom Site Occupancy of H36A       Constrained at        0.5 Check
PLAT300_ALERT_4_G Atom Site Occupancy of H36B       Constrained at        0.5 Check
PLAT300_ALERT_4_G Atom Site Occupancy of H36C       Constrained at        0.5 Check
PLAT300_ALERT_4_G Atom Site Occupancy of H39A       Constrained at        0.5 Check
PLAT300_ALERT_4_G Atom Site Occupancy of H39B       Constrained at        0.5 Check
PLAT300_ALERT_4_G Atom Site Occupancy of H39C       Constrained at        0.5 Check
PLAT300_ALERT_4_G Atom Site Occupancy of H44A       Constrained at        0.5 Check
PLAT300_ALERT_4_G Atom Site Occupancy of H44B       Constrained at        0.5 Check
PLAT300_ALERT_4_G Atom Site Occupancy of H44C       Constrained at        0.5 Check
PLAT300_ALERT_4_G Atom Site Occupancy of H70A       Constrained at        0.5 Check
```

```
PLAT300_ALERT_4_G Atom Site Occupancy of H70B       Constrained at        0.5 Check
PLAT300_ALERT_4_G Atom Site Occupancy of H70C       Constrained at        0.5 Check
PLAT300_ALERT_4_G Atom Site Occupancy of H73A       Constrained at        0.5 Check
PLAT300_ALERT_4_G Atom Site Occupancy of H73B       Constrained at        0.5 Check
PLAT300_ALERT_4_G Atom Site Occupancy of H73C       Constrained at        0.5 Check
PLAT300_ALERT_4_G Atom Site Occupancy of H81A       Constrained at        0.5 Check
PLAT300_ALERT_4_G Atom Site Occupancy of H81B       Constrained at        0.5 Check
PLAT300_ALERT_4_G Atom Site Occupancy of H81C       Constrained at        0.5 Check
PLAT300_ALERT_4_G Atom Site Occupancy of H86A       Constrained at        0.5 Check
PLAT300_ALERT_4_G Atom Site Occupancy of H86B       Constrained at        0.5 Check
PLAT300_ALERT_4_G Atom Site Occupancy of H86C       Constrained at        0.5 Check
PLAT300_ALERT_4_G Atom Site Occupancy of H94A       Constrained at        0.5 Check
PLAT300_ALERT_4_G Atom Site Occupancy of H94B       Constrained at        0.5 Check
PLAT300_ALERT_4_G Atom Site Occupancy of H94C       Constrained at        0.5 Check
PLAT300_ALERT_4_G Atom Site Occupancy of H98A       Constrained at        0.5 Check
PLAT300_ALERT_4_G Atom Site Occupancy of H98B       Constrained at        0.5 Check
PLAT300_ALERT_4_G Atom Site Occupancy of H98C       Constrained at        0.5 Check
PLAT301_ALERT_3_G Main Residue  Disorder ..............(Resd  1  )        11% Note
PLAT412_ALERT_2_G Short Intra XH3 .. XHn     H6AB     ..H58      .       2.11 Ang.
                                                      x,y,z  =      1_555 Check
PLAT412_ALERT_2_G Short Intra XH3 .. XHn     H24B     ..H47      .       1.61 Ang.
                                                      x,y,z  =      1_555 Check
PLAT412_ALERT_2_G Short Intra XH3 .. XHn     H36A     ..H75      .       1.67 Ang.
                                                      x,y,z  =      1_555 Check
PLAT412_ALERT_2_G Short Intra XH3 .. XHn     H37      ..H39C     .       2.12 Ang.
                                                      x,y,z  =      1_555 Check
PLAT606_ALERT_4_G Solvent Accessible VOID(S) in Structure ........          ! Info
PLAT720_ALERT_4_G Number of Unusual/Non-Standard Labels ..........         16 Note
PLAT793_ALERT_4_G Model has Chirality at S7          (Centro SPGR)          S Verify
PLAT794_ALERT_5_G Tentative Bond Valency for Co1       (II)      .       2.16 Info
PLAT794_ALERT_5_G Tentative Bond Valency for Co2       (II)      .       2.08 Info
PLAT794_ALERT_5_G Tentative Bond Valency for Co3       (II)      .       1.98 Info
PLAT794_ALERT_5_G Tentative Bond Valency for Co4       (II)      .       2.03 Info
PLAT860_ALERT_3_G Number of Least-Squares Restraints ............         299 Note
PLAT967_ALERT_5_G Note: Two-Theta Cutoff Value in Embedded .res ..       50.0 Degree
```

---

 0 **ALERT level A** = Most likely a serious problem - resolve or explain
 3 **ALERT level B** = A potentially serious problem, consider carefully
28 **ALERT level C** = Check. Ensure it is not caused by an omission or oversight
95 **ALERT level G** = General information/check it is not something unexpected

 4 ALERT type 1 CIF construction/syntax error, inconsistent or missing data
34 ALERT type 2 Indicator that the structure model may be wrong or deficient
 5 ALERT type 3 Indicator that the structure quality may be low
78 ALERT type 4 Improvement, methodology, query or suggestion
 5 ALERT type 5 Informative message, check

---

It is advisable to attempt to resolve as many as possible of the alerts in all categories. Often the minor alerts point to easily fixed oversights, errors and omissions in your CIF or refinement strategy, so attention to these fine details can be worthwhile. In order to resolve some of the more serious problems it may be necessary to carry out additional measurements or structure refinements. However, the purpose of your study may justify the reported deviations and the more serious of these should normally be commented upon in the discussion or experimental section of a paper or in the "special_details" fields of the CIF. checkCIF was carefully designed to identify outliers and unusual parameters, but every test has its limitations and alerts that are not important in a particular case may appear. Conversely, the absence of alerts does not guarantee there are no aspects of the results needing attention. It is up to the individual to critically assess their own results and, if necessary, seek expert advice.

**Publication of your CIF in IUCr journals**

A basic structural check has been run on your CIF. These basic checks will be run on all CIFs submitted for publication in IUCr journals (*Acta Crystallographica*, *Journal of Applied Crystallography*, *Journal of Synchrotron Radiation*); however, if you intend to submit to *Acta Crystallographica Section C* or *E* or *IUCrData*, you should make sure that full publication checks are run on the final version of your CIF prior to submission.

**Publication of your CIF in other journals**

Please refer to the *Notes for Authors* of the relevant journal for any special instructions relating to CIF submission.

---

**PLATON version of 12/09/2022; check.def file version of 09/08/2022**

**Datablock co-8** - ellipsoid plot

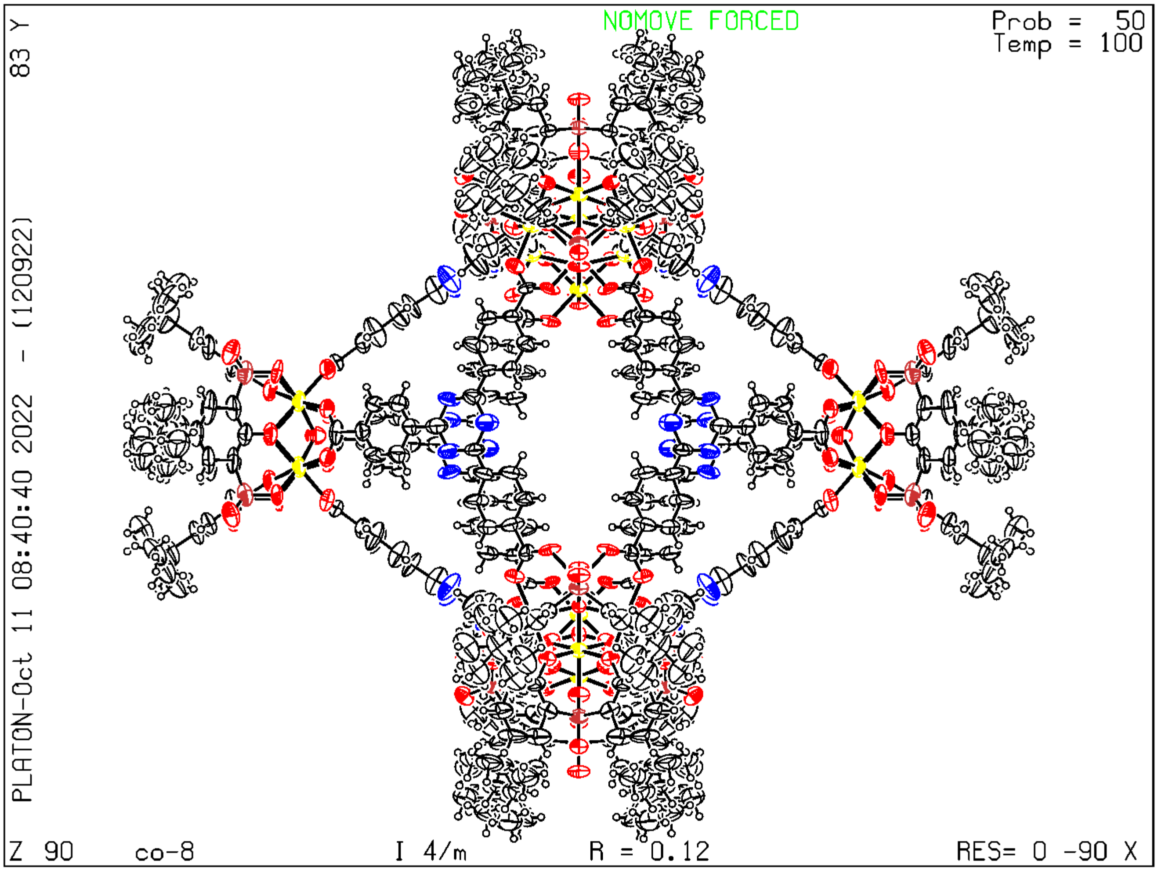

Supplement: Supplementary file 5 — oc3c00027_si_005.pdf [file oc3c00027_si_005.pdf]
