# Supplementary material for: Bridging the Gap between Charge Storage Site and Transportation Pathway in Molecular-Cage-Based Flexible Electrodes
Source: ACS Cent Sci. 2023 Apr 5;9(4):805–15. doi: 10.1021/acscentsci.3c00027 (PMC10141610; doi:10.1021/acscentsci.3c00027)

# checkCIF/PLATON report

You have not supplied any structure factors. As a result the full set of tests cannot be run.

THIS REPORT IS FOR GUIDANCE ONLY. IF USED AS PART OF A REVIEW PROCEDURE FOR PUBLICATION, IT SHOULD NOT REPLACE THE EXPERTISE OF AN EXPERIENCED CRYSTALLOGRAPHIC REFEREE.

No syntax errors found. CIF dictionary Interpreting this report

## Datablock: mg-cage

---

```
Bond precision:    C-C = 0.0071 A          Wavelength=1.54184

Cell:              a=33.0254(9)      b=33.0254(9)      c=54.1285(13)
                   alpha=90          beta=90           gamma=90
Temperature:       100 K
```

| | Calculated | Reported |
|---|---|---|
| Volume | 59037(4) | 59037(4) |
| Space group | I 4/m | I 4/m |
| Hall group | -I 4 | -I 4 |
| Moiety formula | C432 H360 Mg24 N24 O126 S24 [+ solvent] | C432 H360 Mg24 N24 O126 S24 |
| Sum formula | C432 H360 Mg24 N24 O126 S24 [+ solvent] | C432 H360 Mg24 N24 O126 S24 |
| Mr | 9256.34 | 9256.30 |
| Dx,g cm-3 | 0.521 | 0.521 |
| Z | 2 | 2 |
| Mu (mm-1) | 0.811 | 0.811 |
| F000 | 9600.0 | 9600.0 |
| F000' | 9652.12 | |
| h,k,lmax | 37,37,61 | 31,37,61 |
| Nref | 22983 | 22948 |
| Tmin,Tmax | 0.791,0.843 | 0.891,1.000 |
| Tmin' | 0.717 | |

```
Correction method= # Reported T Limits: Tmin=0.891 Tmax=1.000
AbsCorr = MULTI-SCAN

Data completeness= 0.998            Theta(max)= 61.167
```

R(reflections)= 0.0948( 14660)

wR2(reflections)= 0.3192( 22948)

S = 1.123

Npar= 823

---

The following ALERTS were generated. Each ALERT has the format
**test-name_ALERT_alert-type_alert-level.**
Click on the hyperlinks for more details of the test.

---

### Alert level B

THETM01_ALERT_3_B The value of sine(theta_max)/wavelength is less than 0.575
Calculated sin(theta_max)/wavelength = 0.5682
PLAT196_ALERT_1_B No TEMP record and _measurement_temperature .NE. 293 Degree

---

### Alert level C

PLAT084_ALERT_3_C High wR2 Value (i.e. > 0.25) ................... 0.32 Report
PLAT220_ALERT_2_C NonSolvent Resd 1 C Ueq(max)/Ueq(min) Range 3.7 Ratio
PLAT241_ALERT_2_C High 'MainMol' Ueq as Compared to Neighbors of C022 Check
PLAT241_ALERT_2_C High 'MainMol' Ueq as Compared to Neighbors of C023 Check
PLAT242_ALERT_2_C Low 'MainMol' Ueq as Compared to Neighbors of N017 Check
PLAT242_ALERT_2_C Low 'MainMol' Ueq as Compared to Neighbors of C015 Check
PLAT260_ALERT_2_C Large Average Ueq of Residue Including S001 0.103 Check
PLAT334_ALERT_2_C Small <C-C> Benzene Dist. C015 -C01O . 1.37 Ang.
PLAT334_ALERT_2_C Small <C-C> Benzene Dist. C018 -C01R . 1.37 Ang.
PLAT340_ALERT_3_C Low Bond Precision on C-C Bonds ............... 0.00709 Ang.

---

### Alert level G

PLAT002_ALERT_2_G Number of Distance or Angle Restraints on AtSite 28 Note
PLAT003_ALERT_2_G Number of Uiso or Uij Restrained non-H Atoms ... 21 Report
PLAT072_ALERT_2_G SHELXL First Parameter in WGHT Unusually Large 0.20 Report
PLAT172_ALERT_4_G The CIF-Embedded .res File Contains DFIX Records 43 Report
PLAT187_ALERT_4_G The CIF-Embedded .res File Contains RIGU Records 3 Report
PLAT300_ALERT_4_G Atom Site Occupancy of C1 Constrained at 0.5 Check
PLAT300_ALERT_4_G Atom Site Occupancy of C2 Constrained at 0.5 Check
PLAT300_ALERT_4_G Atom Site Occupancy of C3 Constrained at 0.5 Check
PLAT300_ALERT_4_G Atom Site Occupancy of C4 Constrained at 0.5 Check
PLAT300_ALERT_4_G Atom Site Occupancy of C5 Constrained at 0.5 Check
PLAT300_ALERT_4_G Atom Site Occupancy of C6 Constrained at 0.5 Check
PLAT300_ALERT_4_G Atom Site Occupancy of C7 Constrained at 0.5 Check
PLAT300_ALERT_4_G Atom Site Occupancy of C8 Constrained at 0.5 Check
PLAT300_ALERT_4_G Atom Site Occupancy of C9 Constrained at 0.5 Check
PLAT300_ALERT_4_G Atom Site Occupancy of C10 Constrained at 0.5 Check
PLAT300_ALERT_4_G Atom Site Occupancy of C11 Constrained at 0.5 Check
PLAT300_ALERT_4_G Atom Site Occupancy of C12 Constrained at 0.5 Check
PLAT300_ALERT_4_G Atom Site Occupancy of C13 Constrained at 0.5 Check
PLAT300_ALERT_4_G Atom Site Occupancy of C14 Constrained at 0.5 Check
PLAT300_ALERT_4_G Atom Site Occupancy of C15 Constrained at 0.5 Check
PLAT300_ALERT_4_G Atom Site Occupancy of C16 Constrained at 0.5 Check
PLAT300_ALERT_4_G Atom Site Occupancy of C17 Constrained at 0.5 Check
PLAT300_ALERT_4_G Atom Site Occupancy of C0 Constrained at 0.5 Check
PLAT300_ALERT_4_G Atom Site Occupancy of H1A Constrained at 0.5 Check
PLAT300_ALERT_4_G Atom Site Occupancy of H1B Constrained at 0.5 Check
PLAT300_ALERT_4_G Atom Site Occupancy of H1C Constrained at 0.5 Check

```
PLAT300_ALERT_4_G Atom Site Occupancy of H2A        Constrained at        0.5 Check
PLAT300_ALERT_4_G Atom Site Occupancy of H2B        Constrained at        0.5 Check
PLAT300_ALERT_4_G Atom Site Occupancy of H2C        Constrained at        0.5 Check
PLAT300_ALERT_4_G Atom Site Occupancy of H3A        Constrained at        0.5 Check
PLAT300_ALERT_4_G Atom Site Occupancy of H3B        Constrained at        0.5 Check
PLAT300_ALERT_4_G Atom Site Occupancy of H3C        Constrained at        0.5 Check
PLAT300_ALERT_4_G Atom Site Occupancy of H4A        Constrained at        0.5 Check
PLAT300_ALERT_4_G Atom Site Occupancy of H4B        Constrained at        0.5 Check
PLAT300_ALERT_4_G Atom Site Occupancy of H4C        Constrained at        0.5 Check
PLAT300_ALERT_4_G Atom Site Occupancy of H5A        Constrained at        0.5 Check
PLAT300_ALERT_4_G Atom Site Occupancy of H5B        Constrained at        0.5 Check
PLAT300_ALERT_4_G Atom Site Occupancy of H5C        Constrained at        0.5 Check
PLAT300_ALERT_4_G Atom Site Occupancy of H6A        Constrained at        0.5 Check
PLAT300_ALERT_4_G Atom Site Occupancy of H6B        Constrained at        0.5 Check
PLAT300_ALERT_4_G Atom Site Occupancy of H6C        Constrained at        0.5 Check
PLAT300_ALERT_4_G Atom Site Occupancy of H7A        Constrained at        0.5 Check
PLAT300_ALERT_4_G Atom Site Occupancy of H7B        Constrained at        0.5 Check
PLAT300_ALERT_4_G Atom Site Occupancy of H7C        Constrained at        0.5 Check
PLAT300_ALERT_4_G Atom Site Occupancy of H8A        Constrained at        0.5 Check
PLAT300_ALERT_4_G Atom Site Occupancy of H8B        Constrained at        0.5 Check
PLAT300_ALERT_4_G Atom Site Occupancy of H8C        Constrained at        0.5 Check
PLAT300_ALERT_4_G Atom Site Occupancy of H9A        Constrained at        0.5 Check
PLAT300_ALERT_4_G Atom Site Occupancy of H9B        Constrained at        0.5 Check
PLAT300_ALERT_4_G Atom Site Occupancy of H9C        Constrained at        0.5 Check
PLAT300_ALERT_4_G Atom Site Occupancy of H10A       Constrained at        0.5 Check
PLAT300_ALERT_4_G Atom Site Occupancy of H10B       Constrained at        0.5 Check
PLAT300_ALERT_4_G Atom Site Occupancy of H10C       Constrained at        0.5 Check
PLAT300_ALERT_4_G Atom Site Occupancy of H11A       Constrained at        0.5 Check
PLAT300_ALERT_4_G Atom Site Occupancy of H11B       Constrained at        0.5 Check
PLAT300_ALERT_4_G Atom Site Occupancy of H11C       Constrained at        0.5 Check
PLAT300_ALERT_4_G Atom Site Occupancy of H12A       Constrained at        0.5 Check
PLAT300_ALERT_4_G Atom Site Occupancy of H12B       Constrained at        0.5 Check
PLAT300_ALERT_4_G Atom Site Occupancy of H12C       Constrained at        0.5 Check
PLAT300_ALERT_4_G Atom Site Occupancy of H13A       Constrained at        0.5 Check
PLAT300_ALERT_4_G Atom Site Occupancy of H13B       Constrained at        0.5 Check
PLAT300_ALERT_4_G Atom Site Occupancy of H13C       Constrained at        0.5 Check
PLAT300_ALERT_4_G Atom Site Occupancy of H14A       Constrained at        0.5 Check
PLAT300_ALERT_4_G Atom Site Occupancy of H14B       Constrained at        0.5 Check
PLAT300_ALERT_4_G Atom Site Occupancy of H14C       Constrained at        0.5 Check
PLAT300_ALERT_4_G Atom Site Occupancy of H15A       Constrained at        0.5 Check
PLAT300_ALERT_4_G Atom Site Occupancy of H15B       Constrained at        0.5 Check
PLAT300_ALERT_4_G Atom Site Occupancy of H15C       Constrained at        0.5 Check
PLAT300_ALERT_4_G Atom Site Occupancy of H16A       Constrained at        0.5 Check
PLAT300_ALERT_4_G Atom Site Occupancy of H16B       Constrained at        0.5 Check
PLAT300_ALERT_4_G Atom Site Occupancy of H16C       Constrained at        0.5 Check
PLAT300_ALERT_4_G Atom Site Occupancy of H17A       Constrained at        0.5 Check
PLAT300_ALERT_4_G Atom Site Occupancy of H17B       Constrained at        0.5 Check
PLAT300_ALERT_4_G Atom Site Occupancy of H17C       Constrained at        0.5 Check
PLAT300_ALERT_4_G Atom Site Occupancy of H0A        Constrained at        0.5 Check
PLAT300_ALERT_4_G Atom Site Occupancy of H0B        Constrained at        0.5 Check
PLAT300_ALERT_4_G Atom Site Occupancy of H0C        Constrained at        0.5 Check
PLAT301_ALERT_3_G Main Residue  Disorder .............(Resd  1  )        11% Note
PLAT412_ALERT_2_G Short Intra XH3 .. XHn     H01B      ..H2A      .       2.04 Ang.
                                                       x,y,z  =      1_555 Check
PLAT412_ALERT_2_G Short Intra XH3 .. XHn     H01D      ..H0C      .       1.91 Ang.
                                                       x,y,z  =      1_555 Check
PLAT412_ALERT_2_G Short Intra XH3 .. XHn     H01J      ..H10A     .       2.07 Ang.
```

```
                                                      x,y,z  =     1_555 Check
PLAT412_ALERT_2_G Short Intra XH3 .. XHn     H8C      ..H020     .       2.05 Ang.
                                                      x,y,z  =     1_555 Check
PLAT412_ALERT_2_G Short Intra XH3 .. XHn     H01T     ..H13C     .       2.00 Ang.
                                                      x,y,z  =     1_555 Check
PLAT412_ALERT_2_G Short Intra XH3 .. XHn     H01T     ..H16C     .       1.76 Ang.
                                                      x,y,z  =     1_555 Check
PLAT412_ALERT_2_G Short Intra XH3 .. XHn     H11A     ..H020     .       2.10 Ang.
                                                      x,y,z  =     1_555 Check
PLAT412_ALERT_2_G Short Intra XH3 .. XHn     H14C     ..H021     .       2.00 Ang.
                                                      x,y,z  =     1_555 Check
PLAT606_ALERT_4_G Solvent Accessible VOID(S) in Structure .......          ! Info
PLAT720_ALERT_4_G Number of Unusual/Non-Standard Labels .........         95 Note
PLAT764_ALERT_4_G Overcomplete CIF Bond List Detected (Rep/Expd) .       1.16 Ratio
PLAT779_ALERT_4_G Suspect or Irrelevant (Bond) Angle(s) in CIF ...      33.47 Deg.
              O0A  -S001 -MG00    1_555   1_555  11_566 ......     #    1 Check
PLAT779_ALERT_4_G Suspect or Irrelevant (Bond) Angle(s) in CIF ...      33.30 Deg.
              O0C  -S002 -MG      1_555   1_555   1_555 ......     #   11 Check
PLAT779_ALERT_4_G Suspect or Irrelevant (Bond) Angle(s) in CIF ...      34.25 Deg.
              O0E  -S003 -MG1     1_555   1_555   1_555 ......     #   21 Check
PLAT779_ALERT_4_G Suspect or Irrelevant (Bond) Angle(s) in CIF ...      34.23 Deg.
              O00P -S004 -MG08    1_555   1_555   1_555 ......     #   31 Check
PLAT860_ALERT_3_G Number of Least-Squares Restraints ............        169 Note
PLAT941_ALERT_3_G Average HKL Measurement Multiplicity ..........        4.2 Low
PLAT950_ALERT_5_G Calculated (ThMax) and CIF-Reported Hmax Differ          6 Units
```

---

   0 **ALERT level A** = Most likely a serious problem - resolve or explain
   2 **ALERT level B** = A potentially serious problem, consider carefully
  10 **ALERT level C** = Check. Ensure it is not caused by an omission or oversight
  96 **ALERT level G** = General information/check it is not something unexpected

```
   1 ALERT type 1 CIF construction/syntax error, inconsistent or missing data
  19 ALERT type 2 Indicator that the structure model may be wrong or deficient
   6 ALERT type 3 Indicator that the structure quality may be low
  81 ALERT type 4 Improvement, methodology, query or suggestion
   1 ALERT type 5 Informative message, check
```

---

---

It is advisable to attempt to resolve as many as possible of the alerts in all categories. Often the minor alerts point to easily fixed oversights, errors and omissions in your CIF or refinement strategy, so attention to these fine details can be worthwhile. In order to resolve some of the more serious problems it may be necessary to carry out additional measurements or structure refinements. However, the purpose of your study may justify the reported deviations and the more serious of these should normally be commented upon in the discussion or experimental section of a paper or in the "special_details" fields of the CIF. checkCIF was carefully designed to identify outliers and unusual parameters, but every test has its limitations and alerts that are not important in a particular case may appear. Conversely, the absence of alerts does not guarantee there are no aspects of the results needing attention. It is up to the individual to critically assess their own results and, if necessary, seek expert advice.

**Publication of your CIF in IUCr journals**

A basic structural check has been run on your CIF. These basic checks will be run on all CIFs submitted for publication in IUCr journals (*Acta Crystallographica*, *Journal of Applied Crystallography*, *Journal of Synchrotron Radiation*); however, if you intend to submit to *Acta Crystallographica Section C* or *E* or *IUCrData*, you should make sure that full publication checks are run on the final version of your CIF prior to submission.

**Publication of your CIF in other journals**

Please refer to the *Notes for Authors* of the relevant journal for any special instructions relating to CIF submission.

---

**PLATON version of 12/09/2022; check.def file version of 09/08/2022**

**Datablock mg-cage** - ellipsoid plot

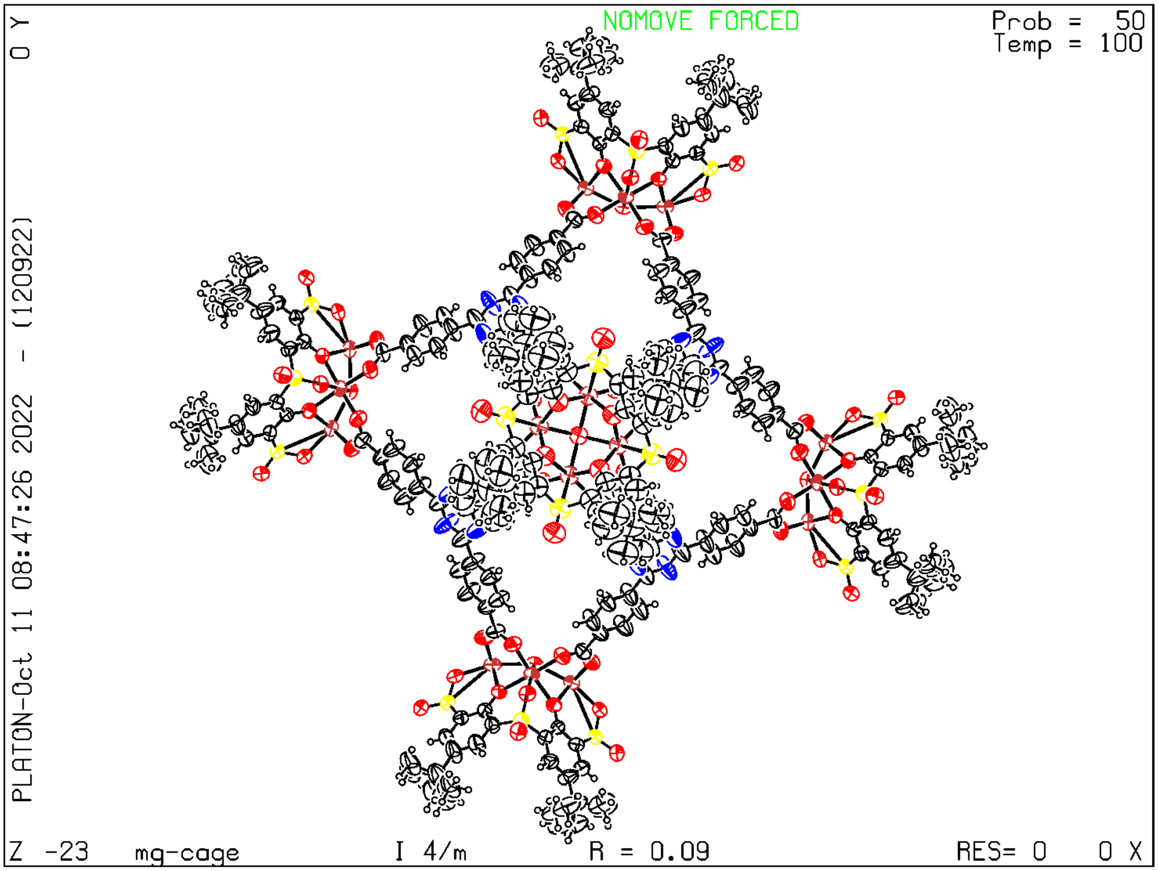

Supplement: Supplementary file 7 — oc3c00027_si_007.pdf [file oc3c00027_si_007.pdf]
