# Supplementary material for: Bridging the Gap between Charge Storage Site and Transportation Pathway in Molecular-Cage-Based Flexible Electrodes
Source: ACS Cent Sci. 2023 Apr 5;9(4):805–15. doi: 10.1021/acscentsci.3c00027 (PMC10141610; doi:10.1021/acscentsci.3c00027)

checkCIF/PLATON report

You have not supplied any structure factors. As a result the full set of tests cannot be run.

THIS REPORT IS FOR GUIDANCE ONLY. IF USED AS PART OF A REVIEW PROCEDURE FOR PUBLICATION, IT SHOULD NOT REPLACE THE EXPERTISE OF AN EXPERIENCED CRYSTALLOGRAPHIC REFEREE.

No syntax errors found.   CIF dictionary   Interpreting this report

Datablock: mn24

```
Bond precision:   C-C = 0.0092 A          Wavelength=1.54184

Cell:              a=33.9461(14)      b=33.9461(14)      c=55.077(3)
                   alpha=90           beta=90            gamma=90
Temperature:       173 K
```

	Calculated	Reported
Volume	63467(6)	63467(6)
Space group	I 4/m	I 4/m
Hall group	-I 4	-I 4
Moiety formula	C432 H360 Mn24 N24 O126 S24 [+ solvent]	C432 H360 Mn24 N24 O126 S24
Sum formula	C432 H360 Mn24 N24 O126 S24 [+ solvent]	C432 H360 Mn24 N24 O126 S24
Mr	9991.46	9991.42
Dx,g cm-3	0.523	0.523
Z	2	2
Mu (mm-1)	2.493	2.493
F000	10224.0	10224.0
F000'	10244.11	
h,k,lmax	38,38,62	32,38,62
Nref	25010	23760
Tmin,Tmax	0.317,0.302	0.690,1.000
Tmin'	0.203	

```
Correction method= # Reported T Limits: Tmin=0.690 Tmax=1.000
AbsCorr = MULTI-SCAN

Data completeness= 0.950            Theta(max)= 61.594
```

R(reflections)= 0.0840(12198) wR2(reflections)= 0.2543(23760)

S = 0.943 Npar= 811

The following ALERTS were generated. Each ALERT has the format
test-name_ALERT_alert-type_alert-level.
Click on the hyperlinks for more details of the test.

Alert level B

```
THETM01_ALERT_3_B  The value of sine(theta_max)/wavelength is less than 0.575
            Calculated sin(theta_max)/wavelength =    0.5705
PLAT029_ALERT_3_B _diffrn_measured_fraction_theta_full value Low .      0.950 Why?
PLAT196_ALERT_1_B No TEMP record and _measurement_temperature .NE.        293 Degree
```

Alert level C

```
PLAT215_ALERT_3_C Disordered C20A      has ADP max/min Ratio .....        3.5 Note
PLAT220_ALERT_2_C NonSolvent   Resd 1  C   Ueq(max)/Ueq(min) Range        4.2 Ratio
PLAT241_ALERT_2_C High   'MainMol' Ueq as Compared to Neighbors of        C33 Check
PLAT241_ALERT_2_C High   'MainMol' Ueq as Compared to Neighbors of        C35 Check
PLAT241_ALERT_2_C High   'MainMol' Ueq as Compared to Neighbors of        C64 Check
PLAT241_ALERT_2_C High   'MainMol' Ueq as Compared to Neighbors of        C65 Check
PLAT241_ALERT_2_C High   'MainMol' Ueq as Compared to Neighbors of       C170 Check
PLAT241_ALERT_2_C High   'MainMol' Ueq as Compared to Neighbors of       C214 Check
PLAT242_ALERT_2_C Low    'MainMol' Ueq as Compared to Neighbors of        C17 Check
PLAT242_ALERT_2_C Low    'MainMol' Ueq as Compared to Neighbors of        C31 Check
PLAT242_ALERT_2_C Low    'MainMol' Ueq as Compared to Neighbors of        C42 Check
PLAT242_ALERT_2_C Low    'MainMol' Ueq as Compared to Neighbors of        C43 Check
PLAT341_ALERT_3_C Low Bond Precision on  C-C Bonds ...............    0.00922 Ang.
```

Alert level G

```
PLAT002_ALERT_2_G Number of Distance or Angle Restraints on AtSite         29 Note
PLAT003_ALERT_2_G Number of Uiso or Uij Restrained non-H Atoms ...         21 Report
PLAT072_ALERT_2_G SHELXL First  Parameter in WGHT  Unusually Large       0.13 Report
PLAT172_ALERT_4_G The CIF-Embedded .res File Contains DFIX Records         52 Report
PLAT186_ALERT_4_G The CIF-Embedded .res File Contains ISOR Records          3 Report
PLAT187_ALERT_4_G The CIF-Embedded .res File Contains RIGU Records          3 Report
PLAT300_ALERT_4_G Atom Site Occupancy of C1         Constrained at        0.5 Check
PLAT300_ALERT_4_G Atom Site Occupancy of C1A        Constrained at        0.5 Check
PLAT300_ALERT_4_G Atom Site Occupancy of C1B        Constrained at        0.5 Check
PLAT300_ALERT_4_G Atom Site Occupancy of C1C        Constrained at        0.5 Check
PLAT300_ALERT_4_G Atom Site Occupancy of C3         Constrained at        0.5 Check
PLAT300_ALERT_4_G Atom Site Occupancy of C6         Constrained at        0.5 Check
PLAT300_ALERT_4_G Atom Site Occupancy of C0         Constrained at        0.5 Check
PLAT300_ALERT_4_G Atom Site Occupancy of C9         Constrained at        0.5 Check
PLAT300_ALERT_4_G Atom Site Occupancy of C10        Constrained at        0.5 Check
PLAT300_ALERT_4_G Atom Site Occupancy of C13        Constrained at        0.5 Check
PLAT300_ALERT_4_G Atom Site Occupancy of C14        Constrained at        0.5 Check
PLAT300_ALERT_4_G Atom Site Occupancy of C14A       Constrained at        0.5 Check
PLAT300_ALERT_4_G Atom Site Occupancy of C14B       Constrained at        0.5 Check
PLAT300_ALERT_4_G Atom Site Occupancy of C19        Constrained at        0.5 Check
PLAT300_ALERT_4_G Atom Site Occupancy of C20A       Constrained at        0.5 Check
PLAT300_ALERT_4_G Atom Site Occupancy of C20B       Constrained at        0.5 Check
```

```
PLAT300_ALERT_4_G Atom Site Occupancy of C24A       Constrained at        0.5 Check
PLAT300_ALERT_4_G Atom Site Occupancy of C24B       Constrained at        0.5 Check
PLAT300_ALERT_4_G Atom Site Occupancy of H1A        Constrained at        0.5 Check
PLAT300_ALERT_4_G Atom Site Occupancy of H1B        Constrained at        0.5 Check
PLAT300_ALERT_4_G Atom Site Occupancy of H1C        Constrained at        0.5 Check
PLAT300_ALERT_4_G Atom Site Occupancy of H0A        Constrained at        0.5 Check
PLAT300_ALERT_4_G Atom Site Occupancy of H3A        Constrained at        0.5 Check
PLAT300_ALERT_4_G Atom Site Occupancy of H3B        Constrained at        0.5 Check
PLAT300_ALERT_4_G Atom Site Occupancy of H3C        Constrained at        0.5 Check
PLAT300_ALERT_4_G Atom Site Occupancy of H0B        Constrained at        0.5 Check
PLAT300_ALERT_4_G Atom Site Occupancy of H0C        Constrained at        0.5 Check
PLAT300_ALERT_4_G Atom Site Occupancy of H6A        Constrained at        0.5 Check
PLAT300_ALERT_4_G Atom Site Occupancy of H6B        Constrained at        0.5 Check
PLAT300_ALERT_4_G Atom Site Occupancy of H6C        Constrained at        0.5 Check
PLAT300_ALERT_4_G Atom Site Occupancy of H1AB       Constrained at        0.5 Check
PLAT300_ALERT_4_G Atom Site Occupancy of H1AC       Constrained at        0.5 Check
PLAT300_ALERT_4_G Atom Site Occupancy of H1AD       Constrained at        0.5 Check
PLAT300_ALERT_4_G Atom Site Occupancy of H9A        Constrained at        0.5 Check
PLAT300_ALERT_4_G Atom Site Occupancy of H9B        Constrained at        0.5 Check
PLAT300_ALERT_4_G Atom Site Occupancy of H9C        Constrained at        0.5 Check
PLAT300_ALERT_4_G Atom Site Occupancy of H1BA       Constrained at        0.5 Check
PLAT300_ALERT_4_G Atom Site Occupancy of H10A       Constrained at        0.5 Check
PLAT300_ALERT_4_G Atom Site Occupancy of H10B       Constrained at        0.5 Check
PLAT300_ALERT_4_G Atom Site Occupancy of H10C       Constrained at        0.5 Check
PLAT300_ALERT_4_G Atom Site Occupancy of H1BB       Constrained at        0.5 Check
PLAT300_ALERT_4_G Atom Site Occupancy of H1BC       Constrained at        0.5 Check
PLAT300_ALERT_4_G Atom Site Occupancy of H1CA       Constrained at        0.5 Check
PLAT300_ALERT_4_G Atom Site Occupancy of H13A       Constrained at        0.5 Check
PLAT300_ALERT_4_G Atom Site Occupancy of H13B       Constrained at        0.5 Check
PLAT300_ALERT_4_G Atom Site Occupancy of H13C       Constrained at        0.5 Check
PLAT300_ALERT_4_G Atom Site Occupancy of H1CB       Constrained at        0.5 Check
PLAT300_ALERT_4_G Atom Site Occupancy of H14A       Constrained at        0.5 Check
PLAT300_ALERT_4_G Atom Site Occupancy of H14B       Constrained at        0.5 Check
PLAT300_ALERT_4_G Atom Site Occupancy of H14C       Constrained at        0.5 Check
PLAT300_ALERT_4_G Atom Site Occupancy of H14D       Constrained at        0.5 Check
PLAT300_ALERT_4_G Atom Site Occupancy of H14E       Constrained at        0.5 Check
PLAT300_ALERT_4_G Atom Site Occupancy of H14F       Constrained at        0.5 Check
PLAT300_ALERT_4_G Atom Site Occupancy of H14G       Constrained at        0.5 Check
PLAT300_ALERT_4_G Atom Site Occupancy of H14H       Constrained at        0.5 Check
PLAT300_ALERT_4_G Atom Site Occupancy of H14I       Constrained at        0.5 Check
PLAT300_ALERT_4_G Atom Site Occupancy of H1CC       Constrained at        0.5 Check
PLAT300_ALERT_4_G Atom Site Occupancy of H19A       Constrained at        0.5 Check
PLAT300_ALERT_4_G Atom Site Occupancy of H19B       Constrained at        0.5 Check
PLAT300_ALERT_4_G Atom Site Occupancy of H19C       Constrained at        0.5 Check
PLAT300_ALERT_4_G Atom Site Occupancy of H20A       Constrained at        0.5 Check
PLAT300_ALERT_4_G Atom Site Occupancy of H20B       Constrained at        0.5 Check
PLAT300_ALERT_4_G Atom Site Occupancy of H20C       Constrained at        0.5 Check
PLAT300_ALERT_4_G Atom Site Occupancy of H20D       Constrained at        0.5 Check
PLAT300_ALERT_4_G Atom Site Occupancy of H20E       Constrained at        0.5 Check
PLAT300_ALERT_4_G Atom Site Occupancy of H20F       Constrained at        0.5 Check
PLAT300_ALERT_4_G Atom Site Occupancy of H24A       Constrained at        0.5 Check
PLAT300_ALERT_4_G Atom Site Occupancy of H24B       Constrained at        0.5 Check
PLAT300_ALERT_4_G Atom Site Occupancy of H24C       Constrained at        0.5 Check
PLAT300_ALERT_4_G Atom Site Occupancy of H24D       Constrained at        0.5 Check
PLAT300_ALERT_4_G Atom Site Occupancy of H24E       Constrained at        0.5 Check
PLAT300_ALERT_4_G Atom Site Occupancy of H24F       Constrained at        0.5 Check
PLAT301_ALERT_3_G Main Residue  Disorder .............(Resd  1  )         11% Note
```

```
PLAT412_ALERT_2_G Short Intra XH3 .. XHn     H24C     ..H71      .       1.99 Ang.
                                                      x,y,z  =      1_555 Check
PLAT412_ALERT_2_G Short Intra XH3 .. XHn     H24D     ..H71      .       2.13 Ang.
                                                      x,y,z  =      1_555 Check
PLAT606_ALERT_4_G Solvent Accessible VOID(S) in Structure ........          ! Info
PLAT720_ALERT_4_G Number of Unusual/Non-Standard Labels ..........         31 Note
PLAT764_ALERT_4_G Overcomplete CIF Bond List Detected (Rep/Expd) .       1.13 Ratio
PLAT793_ALERT_4_G Model has Chirality at S7          (Centro SPGR)          S Verify
PLAT793_ALERT_4_G Model has Chirality at S8          (Centro SPGR)          S Verify
PLAT794_ALERT_5_G Tentative Bond Valency for Mn1       (II)      .       2.16 Info
PLAT794_ALERT_5_G Tentative Bond Valency for Mn2       (II)      .       2.15 Info
PLAT794_ALERT_5_G Tentative Bond Valency for Mn3       (II)      .       2.18 Info
PLAT794_ALERT_5_G Tentative Bond Valency for Mn4       (II)      .       2.22 Info
PLAT860_ALERT_3_G Number of Least-Squares Restraints .............        303 Note
PLAT941_ALERT_3_G Average HKL Measurement Multiplicity ...........        2.1 Low
PLAT950_ALERT_5_G Calculated (ThMax) and CIF-Reported Hmax Differ           6 Units
```

---

0 **ALERT level A** = Most likely a serious problem - resolve or explain
3 **ALERT level B** = A potentially serious problem, consider carefully
13 **ALERT level C** = Check. Ensure it is not caused by an omission or oversight
93 **ALERT level G** = General information/check it is not something unexpected

1 ALERT type 1 CIF construction/syntax error, inconsistent or missing data
16 ALERT type 2 Indicator that the structure model may be wrong or deficient
7 ALERT type 3 Indicator that the structure quality may be low
80 ALERT type 4 Improvement, methodology, query or suggestion
5 ALERT type 5 Informative message, check

It is advisable to attempt to resolve as many as possible of the alerts in all categories. Often the minor alerts point to easily fixed oversights, errors and omissions in your CIF or refinement strategy, so attention to these fine details can be worthwhile. In order to resolve some of the more serious problems it may be necessary to carry out additional measurements or structure refinements. However, the purpose of your study may justify the reported deviations and the more serious of these should normally be commented upon in the discussion or experimental section of a paper or in the "special_details" fields of the CIF. checkCIF was carefully designed to identify outliers and unusual parameters, but every test has its limitations and alerts that are not important in a particular case may appear. Conversely, the absence of alerts does not guarantee there are no aspects of the results needing attention. It is up to the individual to critically assess their own results and, if necessary, seek expert advice.

Publication of your CIF in IUCr journals

A basic structural check has been run on your CIF. These basic checks will be run on all CIFs submitted for publication in IUCr journals (*Acta Crystallographica*, *Journal of Applied Crystallography*, *Journal of Synchrotron Radiation*); however, if you intend to submit to *Acta Crystallographica Section C* or *E* or *IUCrData*, you should make sure that full publication checks are run on the final version of your CIF prior to submission.

Publication of your CIF in other journals

Please refer to the *Notes for Authors* of the relevant journal for any special instructions relating to CIF submission.

PLATON version of 12/09/2022; check.def file version of 09/08/2022

Datablock mn24 - ellipsoid plot

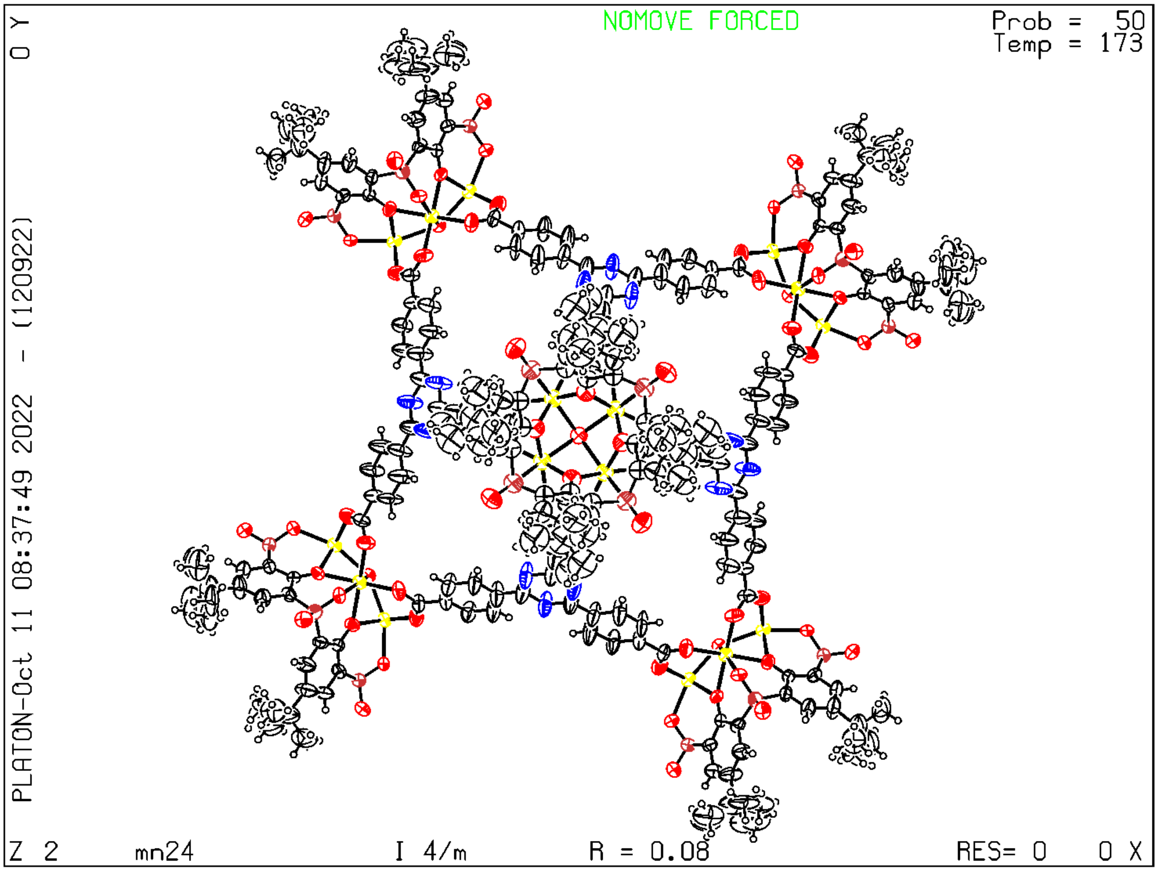

Supplement: Supplementary file 9 — oc3c00027_si_009.pdf [file oc3c00027_si_009.pdf]
