# Supplementary material for: Bridging the Gap between Charge Storage Site and Transportation Pathway in Molecular-Cage-Based Flexible Electrodes
Source: ACS Cent Sci. 2023 Apr 5;9(4):805–15. doi: 10.1021/acscentsci.3c00027 (PMC10141610; doi:10.1021/acscentsci.3c00027)

checkCIF/PLATON report

You have not supplied any structure factors. As a result the full set of tests cannot be run.

THIS REPORT IS FOR GUIDANCE ONLY. IF USED AS PART OF A REVIEW PROCEDURE FOR PUBLICATION, IT SHOULD NOT REPLACE THE EXPERTISE OF AN EXPERIENCED CRYSTALLOGRAPHIC REFEREE.

No syntax errors found.   CIF dictionary   Interpreting this report

Datablock: co-5-20211210

Bond precision: C-C = 0.0078 A   Wavelength=0.71073

Cell:	a=32.9556(7)	b=32.9556(7)	c=54.5250(11)
	alpha=90	beta=90	gamma=90
Temperature:	100 K		

	Calculated	Reported
Volume	59218(3)	59218(3)
Space group	I 4/m	I 4/m
Hall group	-I 4	-I 4
Moiety formula	C452 H396 Co24 N24 O126 S24, 4(C H3) [+ solvent]	C456 H408 Co24 N24 O126 S24
Sum formula	C456 H408 Co24 N24 O126 S24 [+ solvent]	C456 H408 Co24 N24 O126 S24
Mr	10423.85	10423.80
Dx,g cm-3	0.585	0.585
Z	2	2
Mu (mm-1)	0.402	0.402
F000	10704.0	10704.0
F000'	10731.15	
h,k,lmax	39,39,64	39,39,64
Nref	26475	26404
Tmin,Tmax	0.764,0.883	0.811,1.000
Tmin'	0.764	

Correction method= # Reported T Limits: Tmin=0.811 Tmax=1.000
AbsCorr = MULTI-SCAN

Data completeness= 0.997   Theta(max)= 25.026

R(reflections)= 0.0803(14302) | wR2(reflections)= 0.2762(26404)

S = 1.084 | Npar= 936

The following ALERTS were generated. Each ALERT has the format
test-name_ALERT_alert-type_alert-level.
Click on the hyperlinks for more details of the test.

Alert level B

```
PLAT196_ALERT_1_B No TEMP record and _measurement_temperature .NE.       293 Degree
PLAT733_ALERT_1_B Torsion Calc   -161.5(3), Rep     -162(2) ......      6.67 s.u.-R
            CO2 -O1AA-C2AA-C1AA  1_555  1_555  1_555  4_555     #   14 Check
PLAT733_ALERT_1_B Torsion Calc    166.2(3), Rep      166(2) ......      6.67 s.u.-R
            CO3 -O0AA-C2AA-C1AA  1_555  1_555  1_555  4_555     #   18 Check
```

Alert level C

```
PLAT084_ALERT_3_C High wR2 Value (i.e. > 0.25) ...................      0.28 Report
PLAT220_ALERT_2_C NonSolvent   Resd 1  C   Ueq(max)/Ueq(min) Range      3.3 Ratio
PLAT241_ALERT_2_C High   'MainMol' Ueq as Compared to Neighbors of      O1AA Check
PLAT241_ALERT_2_C High   'MainMol' Ueq as Compared to Neighbors of       C58 Check
PLAT241_ALERT_2_C High   'MainMol' Ueq as Compared to Neighbors of       C59 Check
PLAT242_ALERT_2_C Low    'MainMol' Ueq as Compared to Neighbors of       C20 Check
PLAT242_ALERT_2_C Low    'MainMol' Ueq as Compared to Neighbors of       C30 Check
PLAT242_ALERT_2_C Low    'MainMol' Ueq as Compared to Neighbors of       C37 Check
PLAT242_ALERT_2_C Low    'MainMol' Ueq as Compared to Neighbors of       C38 Check
PLAT242_ALERT_2_C Low    'MainMol' Ueq as Compared to Neighbors of       C63 Check
PLAT245_ALERT_2_C U(iso) H57A      Smaller than U(eq) N57       by     0.035 Ang**2
PLAT245_ALERT_2_C U(iso) H57B      Smaller than U(eq) N57       by     0.035 Ang**2
PLAT260_ALERT_2_C Large Average Ueq of Residue Including      Co1      0.110 Check
PLAT260_ALERT_2_C Large Average Ueq of Residue Including     C4AA      0.185 Check
PLAT341_ALERT_3_C Low Bond Precision on  C-C Bonds ...............   0.00785 Ang.
PLAT420_ALERT_2_C D-H Bond Without Acceptor  N10      --H10A     .    Please Check
PLAT420_ALERT_2_C D-H Bond Without Acceptor  N27      --H27A     .    Please Check
PLAT420_ALERT_2_C D-H Bond Without Acceptor  N37      --H37A     .    Please Check
PLAT420_ALERT_2_C D-H Bond Without Acceptor  N37      --H37B     .    Please Check
PLAT420_ALERT_2_C D-H Bond Without Acceptor  N39      --H39B     .    Please Check
PLAT420_ALERT_2_C D-H Bond Without Acceptor  N57      --H57B     .    Please Check
PLAT703_ALERT_1_C Torsion Calc   -161.5(3), Rep     -162(2), Dev..      1.67 Sigma
            CO2 -O1AA-C2AA-C1AA  1_555  1_555  1_555  4_555     #   14 Check
```

Alert level G

```
PLAT002_ALERT_2_G Number of Distance or Angle Restraints on AtSite        37 Note
PLAT003_ALERT_2_G Number of Uiso or Uij Restrained non-H Atoms ...        52 Report
PLAT007_ALERT_5_G Number of Unrefined Donor-H Atoms ..............         8 Report
PLAT012_ALERT_1_G No      _shelx_res_checksum Found in CIF ......    Please Check
PLAT014_ALERT_1_G No      _shelx_fab_checksum Found in CIF ......    Please Check
PLAT042_ALERT_1_G Calc. and Reported MoietyFormula Strings  Differ   Please Check
PLAT063_ALERT_4_G Crystal Size Possibly too Large for Beam Size ..      0.67 mm
PLAT072_ALERT_2_G SHELXL First  Parameter in WGHT  Unusually Large      0.15 Report
PLAT172_ALERT_4_G The CIF-Embedded .res File Contains DFIX Records        54 Report
PLAT174_ALERT_4_G The CIF-Embedded .res File Contains FLAT Records         1 Report
PLAT186_ALERT_4_G The CIF-Embedded .res File Contains ISOR Records         4 Report
```

```
PLAT187_ALERT_4_G The CIF-Embedded .res File Contains RIGU Records          6 Report
PLAT300_ALERT_4_G Atom Site Occupancy of N10       Constrained at        0.5 Check
PLAT300_ALERT_4_G Atom Site Occupancy of N27       Constrained at        0.5 Check
PLAT300_ALERT_4_G Atom Site Occupancy of N37       Constrained at        0.5 Check
PLAT300_ALERT_4_G Atom Site Occupancy of N39       Constrained at        0.5 Check
PLAT300_ALERT_4_G Atom Site Occupancy of C1        Constrained at        0.5 Check
PLAT300_ALERT_4_G Atom Site Occupancy of C0AA      Constrained at        0.5 Check
PLAT300_ALERT_4_G Atom Site Occupancy of C2        Constrained at        0.5 Check
PLAT300_ALERT_4_G Atom Site Occupancy of C3        Constrained at        0.5 Check
PLAT300_ALERT_4_G Atom Site Occupancy of C3AA      Constrained at        0.5 Check
PLAT300_ALERT_4_G Atom Site Occupancy of C5        Constrained at        0.5 Check
PLAT300_ALERT_4_G Atom Site Occupancy of C6        Constrained at        0.5 Check
PLAT300_ALERT_4_G Atom Site Occupancy of C5AA      Constrained at        0.5 Check
PLAT300_ALERT_4_G Atom Site Occupancy of C7        Constrained at        0.5 Check
PLAT300_ALERT_4_G Atom Site Occupancy of C6AA      Constrained at        0.5 Check
PLAT300_ALERT_4_G Atom Site Occupancy of C12       Constrained at        0.5 Check
PLAT300_ALERT_4_G Atom Site Occupancy of C13       Constrained at        0.5 Check
PLAT300_ALERT_4_G Atom Site Occupancy of C17       Constrained at        0.5 Check
PLAT300_ALERT_4_G Atom Site Occupancy of C22       Constrained at        0.5 Check
PLAT300_ALERT_4_G Atom Site Occupancy of C26       Constrained at        0.5 Check
PLAT300_ALERT_4_G Atom Site Occupancy of C27       Constrained at        0.5 Check
PLAT300_ALERT_4_G Atom Site Occupancy of C28       Constrained at        0.5 Check
PLAT300_ALERT_4_G Atom Site Occupancy of C31       Constrained at        0.5 Check
PLAT300_ALERT_4_G Atom Site Occupancy of C43       Constrained at        0.5 Check
PLAT300_ALERT_4_G Atom Site Occupancy of C56       Constrained at        0.5 Check
PLAT300_ALERT_4_G Atom Site Occupancy of C62       Constrained at        0.5 Check
PLAT300_ALERT_4_G Atom Site Occupancy of C71       Constrained at        0.5 Check
PLAT300_ALERT_4_G Atom Site Occupancy of C73       Constrained at        0.5 Check
PLAT300_ALERT_4_G Atom Site Occupancy of C77       Constrained at        0.5 Check
PLAT300_ALERT_4_G Atom Site Occupancy of C78       Constrained at        0.5 Check
PLAT300_ALERT_4_G Atom Site Occupancy of C79       Constrained at        0.5 Check
PLAT300_ALERT_4_G Atom Site Occupancy of C88       Constrained at        0.5 Check
PLAT300_ALERT_4_G Atom Site Occupancy of C89       Constrained at        0.5 Check
PLAT300_ALERT_4_G Atom Site Occupancy of C97       Constrained at        0.5 Check
PLAT300_ALERT_4_G Atom Site Occupancy of C103      Constrained at        0.5 Check
PLAT300_ALERT_4_G Atom Site Occupancy of C112      Constrained at        0.5 Check
PLAT300_ALERT_4_G Atom Site Occupancy of C113      Constrained at        0.5 Check
PLAT300_ALERT_4_G Atom Site Occupancy of C169      Constrained at        0.5 Check
PLAT300_ALERT_4_G Atom Site Occupancy of H3AA      Constrained at        0.5 Check
PLAT300_ALERT_4_G Atom Site Occupancy of H2        Constrained at        0.5 Check
PLAT300_ALERT_4_G Atom Site Occupancy of H3        Constrained at        0.5 Check
PLAT300_ALERT_4_G Atom Site Occupancy of H5AA      Constrained at        0.5 Check
PLAT300_ALERT_4_G Atom Site Occupancy of H5A       Constrained at        0.5 Check
PLAT300_ALERT_4_G Atom Site Occupancy of H5B       Constrained at        0.5 Check
PLAT300_ALERT_4_G Atom Site Occupancy of H5C       Constrained at        0.5 Check
PLAT300_ALERT_4_G Atom Site Occupancy of H5AB      Constrained at        0.5 Check
PLAT300_ALERT_4_G Atom Site Occupancy of H6A       Constrained at        0.5 Check
PLAT300_ALERT_4_G Atom Site Occupancy of H6B       Constrained at        0.5 Check
PLAT300_ALERT_4_G Atom Site Occupancy of H6C       Constrained at        0.5 Check
PLAT300_ALERT_4_G Atom Site Occupancy of H5AC      Constrained at        0.5 Check
PLAT300_ALERT_4_G Atom Site Occupancy of H7A       Constrained at        0.5 Check
PLAT300_ALERT_4_G Atom Site Occupancy of H7B       Constrained at        0.5 Check
PLAT300_ALERT_4_G Atom Site Occupancy of H7C       Constrained at        0.5 Check
PLAT300_ALERT_4_G Atom Site Occupancy of H6AA      Constrained at        0.5 Check
PLAT300_ALERT_4_G Atom Site Occupancy of H6AB      Constrained at        0.5 Check
PLAT300_ALERT_4_G Atom Site Occupancy of H6AC      Constrained at        0.5 Check
PLAT300_ALERT_4_G Atom Site Occupancy of H10A      Constrained at        0.5 Check
```

```
PLAT300_ALERT_4_G Atom Site Occupancy of H10B       Constrained at        0.5 Check
PLAT300_ALERT_4_G Atom Site Occupancy of H11A       Constrained at        0.5 Check
PLAT300_ALERT_4_G Atom Site Occupancy of H11B       Constrained at        0.5 Check
PLAT300_ALERT_4_G Atom Site Occupancy of H11C       Constrained at        0.5 Check
PLAT300_ALERT_4_G Atom Site Occupancy of H12A       Constrained at        0.5 Check
PLAT300_ALERT_4_G Atom Site Occupancy of H12B       Constrained at        0.5 Check
PLAT300_ALERT_4_G Atom Site Occupancy of H12C       Constrained at        0.5 Check
PLAT300_ALERT_4_G Atom Site Occupancy of H13        Constrained at        0.5 Check
PLAT300_ALERT_4_G Atom Site Occupancy of H17A       Constrained at        0.5 Check
PLAT300_ALERT_4_G Atom Site Occupancy of H17B       Constrained at        0.5 Check
PLAT300_ALERT_4_G Atom Site Occupancy of H17C       Constrained at        0.5 Check
PLAT300_ALERT_4_G Atom Site Occupancy of H22        Constrained at        0.5 Check
PLAT300_ALERT_4_G Atom Site Occupancy of H26A       Constrained at        0.5 Check
PLAT300_ALERT_4_G Atom Site Occupancy of H26B       Constrained at        0.5 Check
PLAT300_ALERT_4_G Atom Site Occupancy of H26C       Constrained at        0.5 Check
PLAT300_ALERT_4_G Atom Site Occupancy of H27        Constrained at        0.5 Check
PLAT300_ALERT_4_G Atom Site Occupancy of H27A       Constrained at        0.5 Check
PLAT300_ALERT_4_G Atom Site Occupancy of H27B       Constrained at        0.5 Check
PLAT300_ALERT_4_G Atom Site Occupancy of H28A       Constrained at        0.5 Check
PLAT300_ALERT_4_G Atom Site Occupancy of H28B       Constrained at        0.5 Check
PLAT300_ALERT_4_G Atom Site Occupancy of H28C       Constrained at        0.5 Check
PLAT300_ALERT_4_G Atom Site Occupancy of H31A       Constrained at        0.5 Check
PLAT300_ALERT_4_G Atom Site Occupancy of H31B       Constrained at        0.5 Check
PLAT300_ALERT_4_G Atom Site Occupancy of H31C       Constrained at        0.5 Check
PLAT300_ALERT_4_G Atom Site Occupancy of H37A       Constrained at        0.5 Check
PLAT300_ALERT_4_G Atom Site Occupancy of H37B       Constrained at        0.5 Check
PLAT300_ALERT_4_G Atom Site Occupancy of H39A       Constrained at        0.5 Check
PLAT300_ALERT_4_G Atom Site Occupancy of H39B       Constrained at        0.5 Check
PLAT300_ALERT_4_G Atom Site Occupancy of H43A       Constrained at        0.5 Check
PLAT300_ALERT_4_G Atom Site Occupancy of H43B       Constrained at        0.5 Check
PLAT300_ALERT_4_G Atom Site Occupancy of H43C       Constrained at        0.5 Check
PLAT300_ALERT_4_G Atom Site Occupancy of H56A       Constrained at        0.5 Check
PLAT300_ALERT_4_G Atom Site Occupancy of H56B       Constrained at        0.5 Check
PLAT300_ALERT_4_G Atom Site Occupancy of H56C       Constrained at        0.5 Check
PLAT300_ALERT_4_G Atom Site Occupancy of H62A       Constrained at        0.5 Check
PLAT300_ALERT_4_G Atom Site Occupancy of H62B       Constrained at        0.5 Check
PLAT300_ALERT_4_G Atom Site Occupancy of H62C       Constrained at        0.5 Check
PLAT300_ALERT_4_G Atom Site Occupancy of H73A       Constrained at        0.5 Check
PLAT300_ALERT_4_G Atom Site Occupancy of H73B       Constrained at        0.5 Check
PLAT300_ALERT_4_G Atom Site Occupancy of H73C       Constrained at        0.5 Check
PLAT300_ALERT_4_G Atom Site Occupancy of H77A       Constrained at        0.5 Check
PLAT300_ALERT_4_G Atom Site Occupancy of H77B       Constrained at        0.5 Check
PLAT300_ALERT_4_G Atom Site Occupancy of H77C       Constrained at        0.5 Check
PLAT300_ALERT_4_G Atom Site Occupancy of H78        Constrained at        0.5 Check
PLAT300_ALERT_4_G Atom Site Occupancy of H79        Constrained at        0.5 Check
PLAT300_ALERT_4_G Atom Site Occupancy of H88        Constrained at        0.5 Check
PLAT300_ALERT_4_G Atom Site Occupancy of H97A       Constrained at        0.5 Check
PLAT300_ALERT_4_G Atom Site Occupancy of H97B       Constrained at        0.5 Check
PLAT300_ALERT_4_G Atom Site Occupancy of H97C       Constrained at        0.5 Check
PLAT300_ALERT_4_G Atom Site Occupancy of H103       Constrained at        0.5 Check
PLAT300_ALERT_4_G Atom Site Occupancy of H113       Constrained at        0.5 Check
PLAT300_ALERT_4_G Atom Site Occupancy of H169       Constrained at        0.5 Check
PLAT300_ALERT_4_G Atom Site Occupancy of C4AA       Constrained at        0.5 Check
PLAT300_ALERT_4_G Atom Site Occupancy of H4AA       Constrained at        0.5 Check
PLAT300_ALERT_4_G Atom Site Occupancy of H4AB       Constrained at        0.5 Check
PLAT300_ALERT_4_G Atom Site Occupancy of H4AC       Constrained at        0.5 Check
PLAT301_ALERT_3_G Main Residue  Disorder .............(Resd  1  )         23% Note
```

```
PLAT302_ALERT_4_G Anion/Solvent/Minor-Residue Disorder (Resd  2  )       100% Note
PLAT367_ALERT_2_G Long?  C(sp?)-C(sp?) Bond  C14      - C20       .       1.57 Ang.
PLAT367_ALERT_2_G Long?  C(sp?)-C(sp?) Bond  C18      - C76       .       1.59 Ang.
PLAT410_ALERT_2_G Short Intra H...H Contact  H2       ..H50       .       2.04 Ang.
                                                      x,y,z  =     1_555 Check
PLAT410_ALERT_2_G Short Intra H...H Contact  H50      ..H113      .       2.12 Ang.
                                                      x,y,z  =     1_555 Check
PLAT412_ALERT_2_G Short Intra XH3 .. XHn     H8       ..H97C      .       2.02 Ang.
                                                      x,y,z  =     1_555 Check
PLAT412_ALERT_2_G Short Intra XH3 .. XHn     H12C     ..H54       .       1.95 Ang.
                                                      x,y,z  =     1_555 Check
PLAT412_ALERT_2_G Short Intra XH3 .. XHn     H26B     ..H39       .       2.04 Ang.
                                                      x,y,z  =     1_555 Check
PLAT412_ALERT_2_G Short Intra XH3 .. XHn     H39      ..H77B      .       1.96 Ang.
                                                      x,y,z  =     1_555 Check
PLAT412_ALERT_2_G Short Intra XH3 .. XHn     H54      ..H56A      .       2.06 Ang.
                                                      x,y,z  =     1_555 Check
PLAT432_ALERT_2_G Short Inter X...Y Contact  C4AA     ..C18       .       1.79 Ang.
                                                      x,y,z  =     1_555 Check
PLAT432_ALERT_2_G Short Inter X...Y Contact  C4AA     ..C76       .       2.48 Ang.
                                                      x,y,z  =     1_555 Check
PLAT432_ALERT_2_G Short Inter X...Y Contact  C4AA     ..C60       .       3.01 Ang.
                                                      x,y,z  =     1_555 Check
PLAT606_ALERT_4_G Solvent Accessible VOID(S) in Structure ........          ! Info
PLAT720_ALERT_4_G Number of Unusual/Non-Standard Labels ..........         20 Note
PLAT773_ALERT_2_G Check long C-C Bond in CIF: C18      --C4AA          1.79 Ang.
PLAT790_ALERT_4_G Centre of Gravity not Within Unit Cell: Resd.  #          2 Note
              C H3
PLAT793_ALERT_4_G Model has Chirality at S7          (Centro SPGR)          S Verify
PLAT794_ALERT_5_G Tentative Bond Valency for Co1       (II)       .       2.10 Info
PLAT794_ALERT_5_G Tentative Bond Valency for Co2       (II)       .       2.09 Info
PLAT794_ALERT_5_G Tentative Bond Valency for Co3       (II)       .       2.06 Info
PLAT860_ALERT_3_G Number of Least-Squares Restraints .............        608 Note
```

```
   0 ALERT level A = Most likely a serious problem - resolve or explain
   3 ALERT level B = A potentially serious problem, consider carefully
  22 ALERT level C = Check. Ensure it is not caused by an omission or oversight
 147 ALERT level G = General information/check it is not something unexpected

   7 ALERT type 1 CIF construction/syntax error, inconsistent or missing data
  35 ALERT type 2 Indicator that the structure model may be wrong or deficient
   4 ALERT type 3 Indicator that the structure quality may be low
 122 ALERT type 4 Improvement, methodology, query or suggestion
   4 ALERT type 5 Informative message, check
```

It is advisable to attempt to resolve as many as possible of the alerts in all categories. Often the minor alerts point to easily fixed oversights, errors and omissions in your CIF or refinement strategy, so attention to these fine details can be worthwhile. In order to resolve some of the more serious problems it may be necessary to carry out additional measurements or structure refinements. However, the purpose of your study may justify the reported deviations and the more serious of these should normally be commented upon in the discussion or experimental section of a paper or in the "special_details" fields of the CIF. checkCIF was carefully designed to identify outliers and unusual parameters, but every test has its limitations and alerts that are not important in a particular case may appear. Conversely, the absence of alerts does not guarantee there are no aspects of the results needing attention. It is up to the individual to critically assess their own results and, if necessary, seek expert advice.

Publication of your CIF in IUCr journals

A basic structural check has been run on your CIF. These basic checks will be run on all CIFs submitted for publication in IUCr journals (*Acta Crystallographica*, *Journal of Applied Crystallography*, *Journal of Synchrotron Radiation*); however, if you intend to submit to *Acta Crystallographica Section C* or *E* or *IUCrData*, you should make sure that full publication checks are run on the final version of your CIF prior to submission.

Publication of your CIF in other journals

Please refer to the *Notes for Authors* of the relevant journal for any special instructions relating to CIF submission.

PLATON version of 12/09/2022; check.def file version of 09/08/2022

Datablock co-5-20211210 - ellipsoid plot

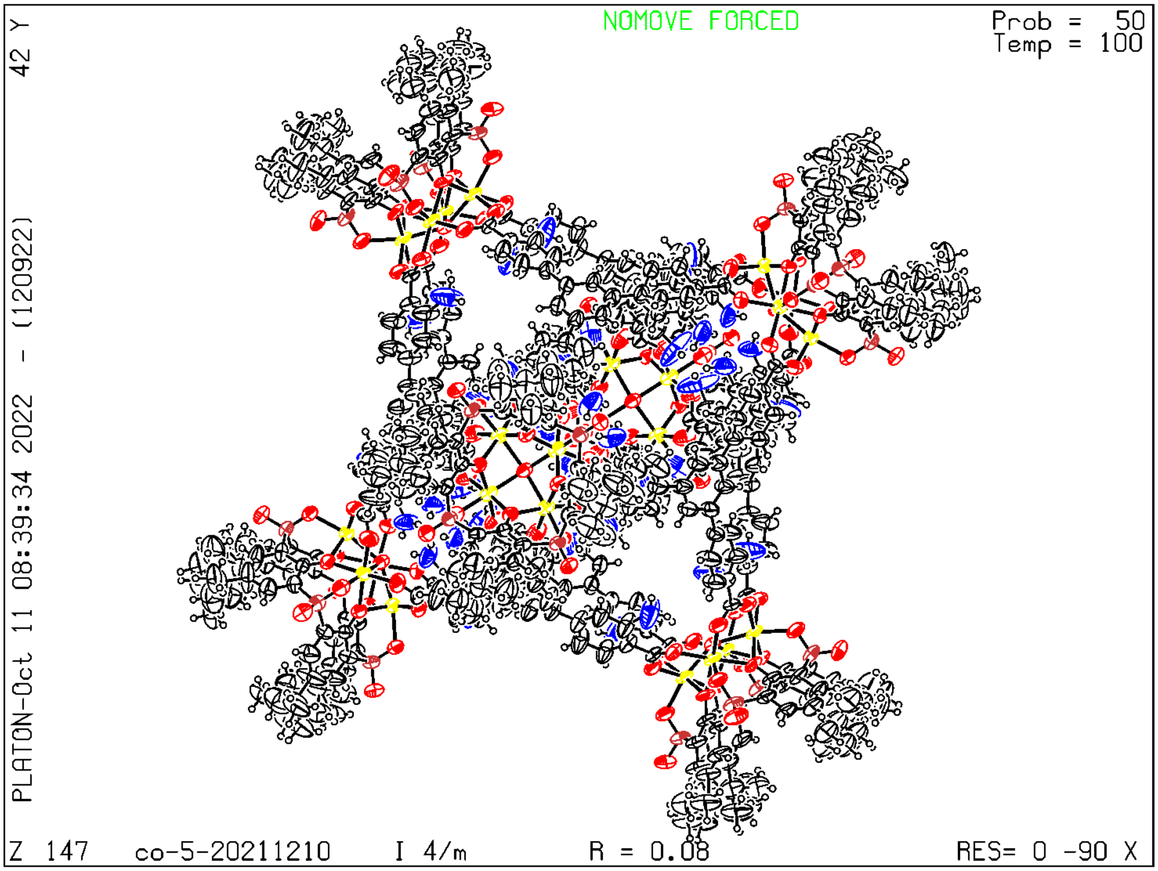

Supplement: Supplementary file 11 — oc3c00027_si_011.pdf [file oc3c00027_si_011.pdf]
