# Supplementary material for: Bridging the Gap between Charge Storage Site and Transportation Pathway in Molecular-Cage-Based Flexible Electrodes
Source: ACS Cent Sci. 2023 Apr 5;9(4):805–15. doi: 10.1021/acscentsci.3c00027 (PMC10141610; doi:10.1021/acscentsci.3c00027)

checkCIF/PLATON report

You have not supplied any structure factors. As a result the full set of tests cannot be run.

THIS REPORT IS FOR GUIDANCE ONLY. IF USED AS PART OF A REVIEW PROCEDURE FOR PUBLICATION, IT SHOULD NOT REPLACE THE EXPERTISE OF AN EXPERIENCED CRYSTALLOGRAPHIC REFEREE.

No syntax errors found. CIF dictionary Interpreting this report

Datablock: mg24

```
Bond precision:   C-C = 0.0062 A          Wavelength=1.54184

Cell:             a=33.5540(12)     b=33.5540(12)     c=54.7983(17)
                  alpha=90          beta=90           gamma=90
Temperature:      173 K
```

	Calculated	Reported
Volume	61696(5)	61696(5)
Space group	I 4/m	I 4/m
Hall group	-I 4	-I 4
Moiety formula	C452 H396 Mg24 N24 O126 S24, 4(C H3) [+ solvent]	C456 H408 Mg24 N24 O126 S24
Sum formula	C456 H408 Mg24 N24 O126 S24 [+ solvent]	C456 H408 Mg24 N24 O126 S24
Mr	9592.95	9592.92
Dx,g cm-3	0.516	0.516
Z	2	2
Mu (mm-1)	0.783	0.783
F000	9984.0	9984.0
F000’	10036.94	
h,k,lmax	38,38,62	36,38,62
Nref	24163	22741
Tmin,Tmax	0.738,0.778	0.939,1.000
Tmin’	0.670	

```
Correction method= # Reported T Limits: Tmin=0.939 Tmax=1.000
AbsCorr = MULTI-SCAN

Data completeness= 0.941            Theta(max)= 61.398
```

R(reflections)= 0.0904(11848)

wR2(reflections)= 0.2971(22741)

S = 0.990 Npar= 962

The following ALERTS were generated. Each ALERT has the format
test-name_ALERT_alert-type_alert-level.
Click on the hyperlinks for more details of the test.

Alert level B

THETM01_ALERT_3_B The value of sine(theta_max)/wavelength is less than 0.575
Calculated sin(theta_max)/wavelength = 0.5694
PLAT029_ALERT_3_B _diffrn_measured_fraction_theta_full value Low . 0.941 Why?
PLAT196_ALERT_1_B No TEMP record and _measurement_temperature .NE. 293 Degree

Alert level C

PLAT084_ALERT_3_C High wR2 Value (i.e. > 0.25) .... 0.30 Report
PLAT220_ALERT_2_C NonSolvent Resd 1 C Ueq(max)/Ueq(min) Range 3.9 Ratio
PLAT241_ALERT_2_C High 'MainMol' Ueq as Compared to Neighbors of O3 Check
PLAT242_ALERT_2_C Low 'MainMol' Ueq as Compared to Neighbors of C31 Check
PLAT260_ALERT_2_C Large Average Ueq of Residue Including C3 0.161 Check
PLAT340_ALERT_3_C Low Bond Precision on C-C Bonds .... 0.00621 Ang.
PLAT420_ALERT_2_C D-H Bond Without Acceptor N12 --H12A . Please Check
PLAT420_ALERT_2_C D-H Bond Without Acceptor N13 --H13B . Please Check
PLAT420_ALERT_2_C D-H Bond Without Acceptor N16 --H16A . Please Check
PLAT420_ALERT_2_C D-H Bond Without Acceptor N24 --H24A . Please Check
PLAT420_ALERT_2_C D-H Bond Without Acceptor N25 --H25B . Please Check

Alert level G

PLAT002_ALERT_2_G Number of Distance or Angle Restraints on AtSite 30 Note
PLAT003_ALERT_2_G Number of Uiso or Uij Restrained non-H Atoms ... 57 Report
PLAT007_ALERT_5_G Number of Unrefined Donor-H Atoms .... 12 Report
PLAT012_ALERT_1_G No _shelx_res_checksum Found in CIF ...... Please Check
PLAT014_ALERT_1_G No _shelx_fab_checksum Found in CIF ...... Please Check
PLAT042_ALERT_1_G Calc. and Reported MoietyFormula Strings Differ Please Check
PLAT072_ALERT_2_G SHELXL First Parameter in WGHT Unusually Large 0.18 Report
PLAT172_ALERT_4_G The CIF-Embedded .res File Contains DFIX Records 41 Report
PLAT186_ALERT_4_G The CIF-Embedded .res File Contains ISOR Records 6 Report
PLAT187_ALERT_4_G The CIF-Embedded .res File Contains RIGU Records 6 Report
PLAT300_ALERT_4_G Atom Site Occupancy of N12 Constrained at 0.5 Check
PLAT300_ALERT_4_G Atom Site Occupancy of N13 Constrained at 0.5 Check
PLAT300_ALERT_4_G Atom Site Occupancy of N16 Constrained at 0.5 Check
PLAT300_ALERT_4_G Atom Site Occupancy of N24 Constrained at 0.5 Check
PLAT300_ALERT_4_G Atom Site Occupancy of N25 Constrained at 0.75 Check
PLAT300_ALERT_4_G Atom Site Occupancy of C1AA Constrained at 0.5 Check
PLAT300_ALERT_4_G Atom Site Occupancy of C14 Constrained at 0.5 Check
PLAT300_ALERT_4_G Atom Site Occupancy of C18 Constrained at 0.75 Check
PLAT300_ALERT_4_G Atom Site Occupancy of C20 Constrained at 0.5 Check
PLAT300_ALERT_4_G Atom Site Occupancy of C22 Constrained at 0.5 Check
PLAT300_ALERT_4_G Atom Site Occupancy of C26 Constrained at 0.5 Check
PLAT300_ALERT_4_G Atom Site Occupancy of C49 Constrained at 0.5 Check
PLAT300_ALERT_4_G Atom Site Occupancy of C51B Constrained at 0.75 Check
PLAT300_ALERT_4_G Atom Site Occupancy of C56A Constrained at 0.75 Check

```
PLAT300_ALERT_4_G Atom Site Occupancy of C59A       Constrained at        0.5 Check
PLAT300_ALERT_4_G Atom Site Occupancy of C59B       Constrained at        0.5 Check
PLAT300_ALERT_4_G Atom Site Occupancy of C60A       Constrained at        0.5 Check
PLAT300_ALERT_4_G Atom Site Occupancy of C60B       Constrained at        0.5 Check
PLAT300_ALERT_4_G Atom Site Occupancy of C63A       Constrained at        0.5 Check
PLAT300_ALERT_4_G Atom Site Occupancy of C63B       Constrained at        0.5 Check
PLAT300_ALERT_4_G Atom Site Occupancy of C64A       Constrained at       0.75 Check
PLAT300_ALERT_4_G Atom Site Occupancy of C69A       Constrained at        0.5 Check
PLAT300_ALERT_4_G Atom Site Occupancy of C69B       Constrained at        0.5 Check
PLAT300_ALERT_4_G Atom Site Occupancy of C70        Constrained at       0.75 Check
PLAT300_ALERT_4_G Atom Site Occupancy of C71A       Constrained at        0.5 Check
PLAT300_ALERT_4_G Atom Site Occupancy of C71B       Constrained at        0.5 Check
PLAT300_ALERT_4_G Atom Site Occupancy of C73        Constrained at        0.5 Check
PLAT300_ALERT_4_G Atom Site Occupancy of C73A       Constrained at       0.75 Check
PLAT300_ALERT_4_G Atom Site Occupancy of C74A       Constrained at        0.5 Check
PLAT300_ALERT_4_G Atom Site Occupancy of C74B       Constrained at        0.5 Check
PLAT300_ALERT_4_G Atom Site Occupancy of C81A       Constrained at        0.5 Check
PLAT300_ALERT_4_G Atom Site Occupancy of C81B       Constrained at        0.5 Check
PLAT300_ALERT_4_G Atom Site Occupancy of C86        Constrained at        0.5 Check
PLAT300_ALERT_4_G Atom Site Occupancy of C90        Constrained at       0.75 Check
PLAT300_ALERT_4_G Atom Site Occupancy of C91        Constrained at        0.5 Check
PLAT300_ALERT_4_G Atom Site Occupancy of C92A       Constrained at        0.5 Check
PLAT300_ALERT_4_G Atom Site Occupancy of C92B       Constrained at        0.5 Check
PLAT300_ALERT_4_G Atom Site Occupancy of C104       Constrained at        0.5 Check
PLAT300_ALERT_4_G Atom Site Occupancy of C105       Constrained at        0.5 Check
PLAT300_ALERT_4_G Atom Site Occupancy of N106       Constrained at       0.25 Check
PLAT300_ALERT_4_G Atom Site Occupancy of C2AA       Constrained at       0.25 Check
PLAT300_ALERT_4_G Atom Site Occupancy of C51A       Constrained at       0.25 Check
PLAT300_ALERT_4_G Atom Site Occupancy of C56B       Constrained at       0.25 Check
PLAT300_ALERT_4_G Atom Site Occupancy of C64B       Constrained at       0.25 Check
PLAT300_ALERT_4_G Atom Site Occupancy of C73B       Constrained at       0.25 Check
PLAT300_ALERT_4_G Atom Site Occupancy of C116       Constrained at       0.25 Check
PLAT300_ALERT_4_G Atom Site Occupancy of C204       Constrained at       0.25 Check
PLAT300_ALERT_4_G Atom Site Occupancy of H1AA       Constrained at        0.5 Check
PLAT300_ALERT_4_G Atom Site Occupancy of H1AB       Constrained at        0.5 Check
PLAT300_ALERT_4_G Atom Site Occupancy of H1AC       Constrained at        0.5 Check
PLAT300_ALERT_4_G Atom Site Occupancy of H10C       Constrained at        0.5 Check
PLAT300_ALERT_4_G Atom Site Occupancy of H10D       Constrained at        0.5 Check
PLAT300_ALERT_4_G Atom Site Occupancy of H10E       Constrained at        0.5 Check
PLAT300_ALERT_4_G Atom Site Occupancy of H10F       Constrained at        0.5 Check
PLAT300_ALERT_4_G Atom Site Occupancy of H10G       Constrained at        0.5 Check
PLAT300_ALERT_4_G Atom Site Occupancy of H10H       Constrained at        0.5 Check
PLAT300_ALERT_4_G Atom Site Occupancy of H12A       Constrained at        0.5 Check
PLAT300_ALERT_4_G Atom Site Occupancy of H12B       Constrained at        0.5 Check
PLAT300_ALERT_4_G Atom Site Occupancy of H13A       Constrained at        0.5 Check
PLAT300_ALERT_4_G Atom Site Occupancy of H13B       Constrained at        0.5 Check
PLAT300_ALERT_4_G Atom Site Occupancy of H14A       Constrained at        0.5 Check
PLAT300_ALERT_4_G Atom Site Occupancy of H14B       Constrained at        0.5 Check
PLAT300_ALERT_4_G Atom Site Occupancy of H14C       Constrained at        0.5 Check
PLAT300_ALERT_4_G Atom Site Occupancy of H16A       Constrained at        0.5 Check
PLAT300_ALERT_4_G Atom Site Occupancy of H16B       Constrained at        0.5 Check
PLAT300_ALERT_4_G Atom Site Occupancy of H18A       Constrained at       0.75 Check
PLAT300_ALERT_4_G Atom Site Occupancy of H18B       Constrained at       0.75 Check
PLAT300_ALERT_4_G Atom Site Occupancy of H18C       Constrained at       0.75 Check
PLAT300_ALERT_4_G Atom Site Occupancy of H20A       Constrained at        0.5 Check
PLAT300_ALERT_4_G Atom Site Occupancy of H20B       Constrained at        0.5 Check
PLAT300_ALERT_4_G Atom Site Occupancy of H20C       Constrained at        0.5 Check
```

```
PLAT300_ALERT_4_G Atom Site Occupancy of H22A       Constrained at        0.5 Check
PLAT300_ALERT_4_G Atom Site Occupancy of H22B       Constrained at        0.5 Check
PLAT300_ALERT_4_G Atom Site Occupancy of H22C       Constrained at        0.5 Check
PLAT300_ALERT_4_G Atom Site Occupancy of H24A       Constrained at        0.5 Check
PLAT300_ALERT_4_G Atom Site Occupancy of H24B       Constrained at        0.5 Check
PLAT300_ALERT_4_G Atom Site Occupancy of H25A       Constrained at       0.75 Check
PLAT300_ALERT_4_G Atom Site Occupancy of H25B       Constrained at       0.75 Check
PLAT300_ALERT_4_G Atom Site Occupancy of H26A       Constrained at        0.5 Check
PLAT300_ALERT_4_G Atom Site Occupancy of H26B       Constrained at        0.5 Check
PLAT300_ALERT_4_G Atom Site Occupancy of H26C       Constrained at        0.5 Check
PLAT300_ALERT_4_G Atom Site Occupancy of H49A       Constrained at        0.5 Check
PLAT300_ALERT_4_G Atom Site Occupancy of H49B       Constrained at        0.5 Check
PLAT300_ALERT_4_G Atom Site Occupancy of H49C       Constrained at        0.5 Check
PLAT300_ALERT_4_G Atom Site Occupancy of H51B       Constrained at       0.75 Check
PLAT300_ALERT_4_G Atom Site Occupancy of H59A       Constrained at        0.5 Check
PLAT300_ALERT_4_G Atom Site Occupancy of H60A       Constrained at        0.5 Check
PLAT300_ALERT_4_G Atom Site Occupancy of H60B       Constrained at        0.5 Check
PLAT300_ALERT_4_G Atom Site Occupancy of H63A       Constrained at        0.5 Check
PLAT300_ALERT_4_G Atom Site Occupancy of H64A       Constrained at       0.75 Check
PLAT300_ALERT_4_G Atom Site Occupancy of H69A       Constrained at        0.5 Check
PLAT300_ALERT_4_G Atom Site Occupancy of H70A       Constrained at       0.75 Check
PLAT300_ALERT_4_G Atom Site Occupancy of H70B       Constrained at       0.75 Check
PLAT300_ALERT_4_G Atom Site Occupancy of H70C       Constrained at       0.75 Check
PLAT300_ALERT_4_G Atom Site Occupancy of H71A       Constrained at        0.5 Check
PLAT300_ALERT_4_G Atom Site Occupancy of H71B       Constrained at        0.5 Check
PLAT300_ALERT_4_G Atom Site Occupancy of H73A       Constrained at       0.75 Check
PLAT300_ALERT_4_G Atom Site Occupancy of H73B       Constrained at        0.5 Check
PLAT300_ALERT_4_G Atom Site Occupancy of H73C       Constrained at        0.5 Check
PLAT300_ALERT_4_G Atom Site Occupancy of H73D       Constrained at        0.5 Check
PLAT300_ALERT_4_G Atom Site Occupancy of H74A       Constrained at        0.5 Check
PLAT300_ALERT_4_G Atom Site Occupancy of H74B       Constrained at        0.5 Check
PLAT300_ALERT_4_G Atom Site Occupancy of H81A       Constrained at        0.5 Check
PLAT300_ALERT_4_G Atom Site Occupancy of H86A       Constrained at        0.5 Check
PLAT300_ALERT_4_G Atom Site Occupancy of H86B       Constrained at        0.5 Check
PLAT300_ALERT_4_G Atom Site Occupancy of H86C       Constrained at        0.5 Check
PLAT300_ALERT_4_G Atom Site Occupancy of H90A       Constrained at       0.75 Check
PLAT300_ALERT_4_G Atom Site Occupancy of H90B       Constrained at       0.75 Check
PLAT300_ALERT_4_G Atom Site Occupancy of H90C       Constrained at       0.75 Check
PLAT300_ALERT_4_G Atom Site Occupancy of H91A       Constrained at        0.5 Check
PLAT300_ALERT_4_G Atom Site Occupancy of H91B       Constrained at        0.5 Check
PLAT300_ALERT_4_G Atom Site Occupancy of H91C       Constrained at        0.5 Check
PLAT300_ALERT_4_G Atom Site Occupancy of H92A       Constrained at        0.5 Check
PLAT300_ALERT_4_G Atom Site Occupancy of H92B       Constrained at        0.5 Check
PLAT300_ALERT_4_G Atom Site Occupancy of H2AA       Constrained at       0.25 Check
PLAT300_ALERT_4_G Atom Site Occupancy of H2AB       Constrained at       0.25 Check
PLAT300_ALERT_4_G Atom Site Occupancy of H2AC       Constrained at       0.25 Check
PLAT300_ALERT_4_G Atom Site Occupancy of H10A       Constrained at       0.25 Check
PLAT300_ALERT_4_G Atom Site Occupancy of H10B       Constrained at       0.25 Check
PLAT300_ALERT_4_G Atom Site Occupancy of H11A       Constrained at       0.25 Check
PLAT300_ALERT_4_G Atom Site Occupancy of H11B       Constrained at       0.25 Check
PLAT300_ALERT_4_G Atom Site Occupancy of H11C       Constrained at       0.25 Check
PLAT300_ALERT_4_G Atom Site Occupancy of H20D       Constrained at       0.25 Check
PLAT300_ALERT_4_G Atom Site Occupancy of H20E       Constrained at       0.25 Check
PLAT300_ALERT_4_G Atom Site Occupancy of H20F       Constrained at       0.25 Check
PLAT300_ALERT_4_G Atom Site Occupancy of H51A       Constrained at       0.25 Check
PLAT300_ALERT_4_G Atom Site Occupancy of H56B       Constrained at       0.25 Check
PLAT300_ALERT_4_G Atom Site Occupancy of H64B       Constrained at       0.25 Check
```

```
PLAT300_ALERT_4_G Atom Site Occupancy of C3        Constrained at       0.5 Check
PLAT300_ALERT_4_G Atom Site Occupancy of H3A       Constrained at       0.5 Check
PLAT300_ALERT_4_G Atom Site Occupancy of H3B       Constrained at       0.5 Check
PLAT300_ALERT_4_G Atom Site Occupancy of H3C       Constrained at       0.5 Check
PLAT301_ALERT_3_G Main Residue  Disorder .............(Resd  1  )        29% Note
PLAT302_ALERT_4_G Anion/Solvent/Minor-Residue Disorder (Resd  2  )      100% Note
PLAT335_ALERT_2_G Check Large C6 Ring C-C Range C25      -C69B          0.15 Ang.
PLAT410_ALERT_2_G Short Intra H...H Contact  H36      ..H60B     .      2.13 Ang.
                                                      x,y,z  =     1_555 Check
PLAT410_ALERT_2_G Short Intra H...H Contact  H36      ..H74A     .      2.13 Ang.
                                                      x,y,z  =     1_555 Check
PLAT410_ALERT_2_G Short Intra H...H Contact  H36      ..H74B     .      2.09 Ang.
                                                      x,y,z  =     1_555 Check
PLAT410_ALERT_2_G Short Intra H...H Contact  H38      ..H71B     .      2.07 Ang.
                                                      x,y,z  =     1_555 Check
PLAT410_ALERT_2_G Short Intra H...H Contact  H38      ..H64B     .      1.95 Ang.
                                                      x,y,z  =     1_555 Check
PLAT412_ALERT_2_G Short Intra XH3 .. XHn     H0AA     ..H49A     .      1.99 Ang.
                                                      x,y,z  =     1_555 Check
PLAT412_ALERT_2_G Short Intra XH3 .. XHn     H0AA     ..H73B     .      2.01 Ang.
                                                      x,y,z  =     1_555 Check
PLAT412_ALERT_2_G Short Intra XH3 .. XHn     H6       ..H14A     .      1.92 Ang.
                                                      x,y,z  =     1_555 Check
PLAT412_ALERT_2_G Short Intra XH3 .. XHn     H6       ..H86A     .      1.98 Ang.
                                                      x,y,z  =     1_555 Check
PLAT412_ALERT_2_G Short Intra XH3 .. XHn     H52      ..H2AA     .      1.77 Ang.
                                                      x,y,z  =     1_555 Check
PLAT432_ALERT_2_G Short Inter X...Y Contact  C1       ..C3       .      1.78 Ang.
                                                      x,y,z  =     1_555 Check
PLAT432_ALERT_2_G Short Inter X...Y Contact  C3       ..C7       .      2.60 Ang.
                                                      x,y,z  =     1_555 Check
PLAT432_ALERT_2_G Short Inter X...Y Contact  C3       ..C11      .      3.20 Ang.
                                                      x,y,z  =     1_555 Check
PLAT606_ALERT_4_G Solvent Accessible VOID(S) in Structure .......          ! Info
PLAT720_ALERT_4_G Number of Unusual/Non-Standard Labels .........         10 Note
PLAT764_ALERT_4_G Overcomplete CIF Bond List Detected (Rep/Expd) .      1.12 Ratio
PLAT773_ALERT_2_G Check long C-C Bond in CIF: C1       --C3             1.78 Ang.
PLAT779_ALERT_4_G Suspect or Irrelevant (Bond) Angle(s) in CIF ...     33.24 Deg.
             O2   -S1   -MG4     1_555   1_555   1_555 ......     #    1 Check
PLAT779_ALERT_4_G Suspect or Irrelevant (Bond) Angle(s) in CIF ...     34.19 Deg.
             O13  -S2   -MG5     1_555   1_555   1_555 ......     #   18 Check
PLAT779_ALERT_4_G Suspect or Irrelevant (Bond) Angle(s) in CIF ...     33.34 Deg.
             O1   -S3   -MG6     1_555   1_555   1_555 ......     #   21 Check
PLAT779_ALERT_4_G Suspect or Irrelevant (Bond) Angle(s) in CIF ...     33.84 Deg.
             O16  -S7   -MG8     1_555   1_555   1_555 ......     #  142 Check
PLAT811_ALERT_5_G No ADDSYM Analysis: Too Many Excluded Atoms ....         ! Info
PLAT860_ALERT_3_G Number of Least-Squares Restraints ............        776 Note
PLAT950_ALERT_5_G Calculated (ThMax) and CIF-Reported Hmax Differ          2 Units
```

---

0 **ALERT level A** = Most likely a serious problem - resolve or explain
3 **ALERT level B** = A potentially serious problem, consider carefully
11 **ALERT level C** = Check. Ensure it is not caused by an omission or oversight
169 **ALERT level G** = General information/check it is not something unexpected

4 ALERT type 1 CIF construction/syntax error, inconsistent or missing data

```
 27 ALERT type 2 Indicator that the structure model may be wrong or deficient
  6 ALERT type 3 Indicator that the structure quality may be low
143 ALERT type 4 Improvement, methodology, query or suggestion
  3 ALERT type 5 Informative message, check
```

It is advisable to attempt to resolve as many as possible of the alerts in all categories. Often the minor alerts point to easily fixed oversights, errors and omissions in your CIF or refinement strategy, so attention to these fine details can be worthwhile. In order to resolve some of the more serious problems it may be necessary to carry out additional measurements or structure refinements. However, the purpose of your study may justify the reported deviations and the more serious of these should normally be commented upon in the discussion or experimental section of a paper or in the "special_details" fields of the CIF. checkCIF was carefully designed to identify outliers and unusual parameters, but every test has its limitations and alerts that are not important in a particular case may appear. Conversely, the absence of alerts does not guarantee there are no aspects of the results needing attention. It is up to the individual to critically assess their own results and, if necessary, seek expert advice.

Publication of your CIF in IUCr journals

A basic structural check has been run on your CIF. These basic checks will be run on all CIFs submitted for publication in IUCr journals (*Acta Crystallographica*, *Journal of Applied Crystallography*, *Journal of Synchrotron Radiation*); however, if you intend to submit to *Acta Crystallographica Section C* or *E* or *IUCrData*, you should make sure that full publication checks are run on the final version of your CIF prior to submission.

Publication of your CIF in other journals

Please refer to the *Notes for Authors* of the relevant journal for any special instructions relating to CIF submission.

PLATON version of 12/09/2022; check.def file version of 09/08/2022

Datablock mg24 - ellipsoid plot

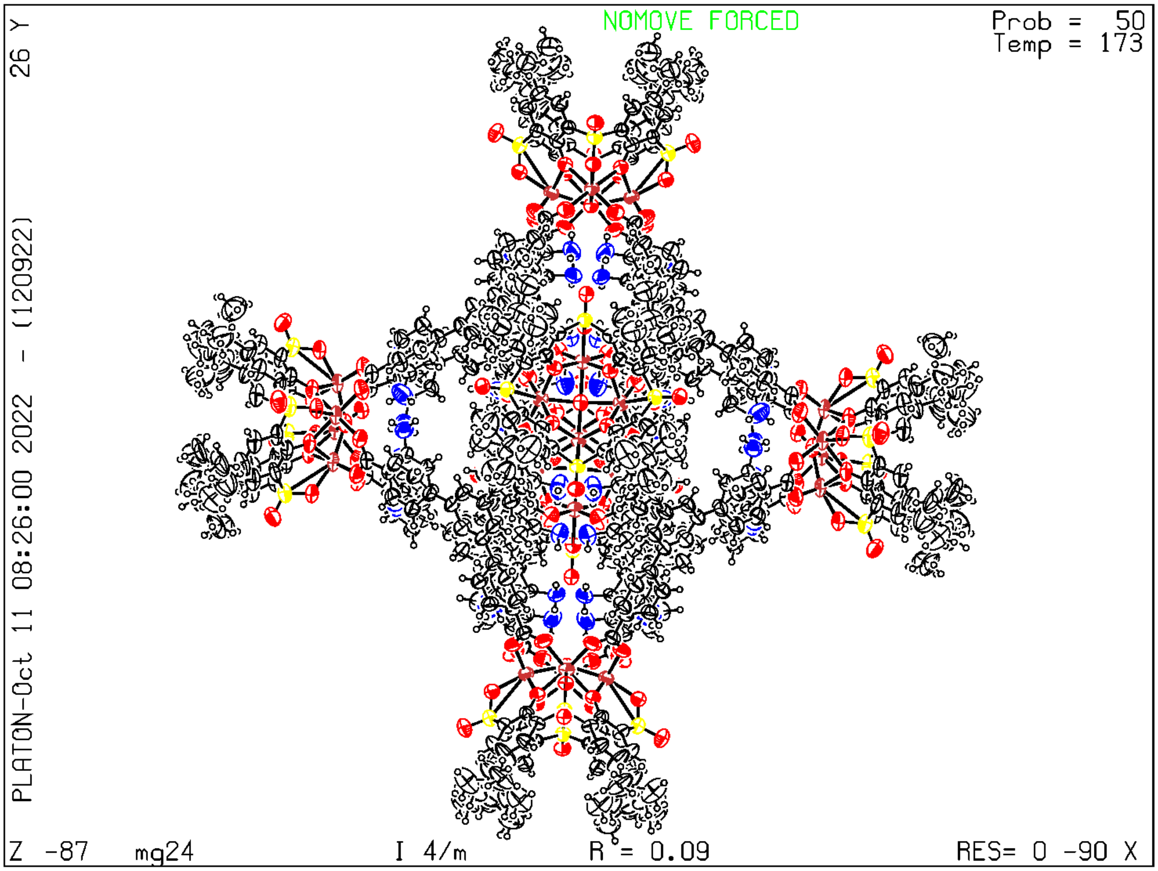

Supplement: Supplementary file 13 — oc3c00027_si_013.pdf [file oc3c00027_si_013.pdf]
