# Supplementary material for: Bridging the Gap between Charge Storage Site and Transportation Pathway in Molecular-Cage-Based Flexible Electrodes
Source: ACS Cent Sci. 2023 Apr 5;9(4):805–15. doi: 10.1021/acscentsci.3c00027 (PMC10141610; doi:10.1021/acscentsci.3c00027)

checkCIF/PLATON report

You have not supplied any structure factors. As a result the full set of tests cannot be run.

THIS REPORT IS FOR GUIDANCE ONLY. IF USED AS PART OF A REVIEW PROCEDURE FOR PUBLICATION, IT SHOULD NOT REPLACE THE EXPERTISE OF AN EXPERIENCED CRYSTALLOGRAPHIC REFEREE.

No syntax errors found. CIF dictionary Interpreting this report

Datablock: mn24

Bond precision:	C-C = 0.0122 A		Wavelength=1.54184
Cell:	a=33.8607(19)	b=33.8607(19)	c=55.247(3)
	alpha=90	beta=90	gamma=90
Temperature:	173 K		

	Calculated	Reported
Volume	63343(8)	63344(8)
Space group	I 4/m	I 4/m
Hall group	-I 4	-I 4
Moiety formula	C456 H408 Mn24 N24 O126 S24 [+ solvent]	C456 H408 Mn24 N24 O126 S24
Sum formula	C456 H408 Mn24 N24 O126 S24 [+ solvent]	C456 H408 Mn24 N24 O126 S24
Mr	10328.08	10328.04
Dx,g cm-3	0.541	0.541
Z	2	2
Mu (mm-1)	2.505	2.505
F000	10608.0	10608.0
F000'	10628.89	
h,k,lmax	38,38,62	38,38,62
Nref	24773	23299
Tmin,Tmax	0.620,0.687	0.677,1.000
Tmin'	0.563	

Correction method= # Reported T Limits: Tmin=0.677 Tmax=1.000
AbsCorr = MULTI-SCAN

Data completeness= 0.940 Theta(max)= 61.307

R(reflections)= 0.0844(7749) wR2(reflections)= 0.2600(23299)

S = 0.874 Npar= 973

The following ALERTS were generated. Each ALERT has the format
test-name_ALERT_alert-type_alert-level.
Click on the hyperlinks for more details of the test.

Alert level B

```
THETM01_ALERT_3_B  The value of sine(theta_max)/wavelength is less than 0.575
            Calculated sin(theta_max)/wavelength =    0.5689
PLAT026_ALERT_3_B Ratio Observed / Unique Reflections (too) Low ..        33% Check
PLAT029_ALERT_3_B _diffrn_measured_fraction_theta_full value Low .      0.940 Why?
PLAT196_ALERT_1_B No TEMP record and _measurement_temperature .NE.        293 Degree
```

Alert level C

```
RINTA01_ALERT_3_C  The value of Rint is greater than 0.12
            Rint given   0.139
PLAT020_ALERT_3_C The Value of Rint is Greater Than 0.12 .........      0.139 Report
PLAT084_ALERT_3_C High wR2 Value (i.e. > 0.25) ...................       0.26 Report
PLAT220_ALERT_2_C NonSolvent   Resd 1  C   Ueq(max)/Ueq(min) Range        3.6 Ratio
PLAT241_ALERT_2_C High   'MainMol' Ueq as Compared to Neighbors of       O1AA Check
PLAT241_ALERT_2_C High   'MainMol' Ueq as Compared to Neighbors of       O0AA Check
PLAT241_ALERT_2_C High   'MainMol' Ueq as Compared to Neighbors of         O9 Check
PLAT241_ALERT_2_C High   'MainMol' Ueq as Compared to Neighbors of        O28 Check
PLAT241_ALERT_2_C High   'MainMol' Ueq as Compared to Neighbors of        C51 Check
PLAT242_ALERT_2_C Low    'MainMol' Ueq as Compared to Neighbors of         C6 Check
PLAT242_ALERT_2_C Low    'MainMol' Ueq as Compared to Neighbors of        C79 Check
PLAT260_ALERT_2_C Large Average Ueq of Residue Including       Mn1      0.120 Check
PLAT341_ALERT_3_C Low Bond Precision on  C-C Bonds ...............    0.01215 Ang.
PLAT363_ALERT_2_C Long   C(sp3)-C(sp2) Bond  C51      - C76      .       1.65 Ang.
PLAT369_ALERT_2_C Long   C(sp2)-C(sp2) Bond  C38      - C49      .       1.54 Ang.
PLAT420_ALERT_2_C D-H Bond Without Acceptor  N2       --H2B      .     Please Check
PLAT420_ALERT_2_C D-H Bond Without Acceptor  N20      --H20B     .     Please Check
PLAT420_ALERT_2_C D-H Bond Without Acceptor  N22      --H22A     .     Please Check
PLAT420_ALERT_2_C D-H Bond Without Acceptor  N26      --H26A     .     Please Check
PLAT420_ALERT_2_C D-H Bond Without Acceptor  N29      --H29A     .     Please Check
PLAT420_ALERT_2_C D-H Bond Without Acceptor  N29      --H29B     .     Please Check
```

Alert level G

```
PLAT002_ALERT_2_G Number of Distance or Angle Restraints on AtSite         37 Note
PLAT003_ALERT_2_G Number of Uiso or Uij Restrained non-H Atoms ...         57 Report
PLAT007_ALERT_5_G Number of Unrefined Donor-H Atoms ..............         12 Report
PLAT012_ALERT_1_G No       _shelx_res_checksum Found in CIF ......     Please Check
PLAT014_ALERT_1_G No       _shelx_fab_checksum Found in CIF ......     Please Check
PLAT072_ALERT_2_G SHELXL First  Parameter in WGHT  Unusually Large       0.11 Report
PLAT172_ALERT_4_G The CIF-Embedded .res File Contains DFIX Records         54 Report
PLAT174_ALERT_4_G The CIF-Embedded .res File Contains FLAT Records          1 Report
PLAT186_ALERT_4_G The CIF-Embedded .res File Contains ISOR Records          6 Report
PLAT187_ALERT_4_G The CIF-Embedded .res File Contains RIGU Records          6 Report
PLAT300_ALERT_4_G Atom Site Occupancy of N2          Constrained at         0.5 Check
PLAT300_ALERT_4_G Atom Site Occupancy of N20         Constrained at         0.5 Check
```

```
PLAT300_ALERT_4_G Atom Site Occupancy of N22        Constrained at        0.5 Check
PLAT300_ALERT_4_G Atom Site Occupancy of N26        Constrained at        0.5 Check
PLAT300_ALERT_4_G Atom Site Occupancy of N29        Constrained at     0.6667 Check
PLAT300_ALERT_4_G Atom Site Occupancy of C1         Constrained at        0.5 Check
PLAT300_ALERT_4_G Atom Site Occupancy of C4AA       Constrained at        0.5 Check
PLAT300_ALERT_4_G Atom Site Occupancy of C0AA       Constrained at        0.5 Check
PLAT300_ALERT_4_G Atom Site Occupancy of C3         Constrained at        0.5 Check
PLAT300_ALERT_4_G Atom Site Occupancy of C1AA       Constrained at        0.5 Check
PLAT300_ALERT_4_G Atom Site Occupancy of C4         Constrained at        0.5 Check
PLAT300_ALERT_4_G Atom Site Occupancy of C3AA       Constrained at     0.6667 Check
PLAT300_ALERT_4_G Atom Site Occupancy of C2AA       Constrained at        0.5 Check
PLAT300_ALERT_4_G Atom Site Occupancy of C7AA       Constrained at        0.5 Check
PLAT300_ALERT_4_G Atom Site Occupancy of C10A       Constrained at        0.5 Check
PLAT300_ALERT_4_G Atom Site Occupancy of C10B       Constrained at        0.5 Check
PLAT300_ALERT_4_G Atom Site Occupancy of C19        Constrained at        0.5 Check
PLAT300_ALERT_4_G Atom Site Occupancy of C54B       Constrained at     0.6667 Check
PLAT300_ALERT_4_G Atom Site Occupancy of C61        Constrained at        0.5 Check
PLAT300_ALERT_4_G Atom Site Occupancy of C61A       Constrained at        0.5 Check
PLAT300_ALERT_4_G Atom Site Occupancy of C61B       Constrained at        0.5 Check
PLAT300_ALERT_4_G Atom Site Occupancy of C64B       Constrained at     0.6667 Check
PLAT300_ALERT_4_G Atom Site Occupancy of C67A       Constrained at        0.5 Check
PLAT300_ALERT_4_G Atom Site Occupancy of C67B       Constrained at        0.5 Check
PLAT300_ALERT_4_G Atom Site Occupancy of C70A       Constrained at        0.5 Check
PLAT300_ALERT_4_G Atom Site Occupancy of C70B       Constrained at        0.5 Check
PLAT300_ALERT_4_G Atom Site Occupancy of C83        Constrained at     0.6667 Check
PLAT300_ALERT_4_G Atom Site Occupancy of C85        Constrained at        0.5 Check
PLAT300_ALERT_4_G Atom Site Occupancy of C85A       Constrained at     0.6667 Check
PLAT300_ALERT_4_G Atom Site Occupancy of C86        Constrained at        0.5 Check
PLAT300_ALERT_4_G Atom Site Occupancy of C87        Constrained at     0.6667 Check
PLAT300_ALERT_4_G Atom Site Occupancy of C89        Constrained at     0.6667 Check
PLAT300_ALERT_4_G Atom Site Occupancy of C92A       Constrained at        0.5 Check
PLAT300_ALERT_4_G Atom Site Occupancy of C92B       Constrained at        0.5 Check
PLAT300_ALERT_4_G Atom Site Occupancy of C93A       Constrained at        0.5 Check
PLAT300_ALERT_4_G Atom Site Occupancy of C93B       Constrained at        0.5 Check
PLAT300_ALERT_4_G Atom Site Occupancy of C114       Constrained at        0.5 Check
PLAT300_ALERT_4_G Atom Site Occupancy of C124       Constrained at        0.5 Check
PLAT300_ALERT_4_G Atom Site Occupancy of C163       Constrained at        0.5 Check
PLAT300_ALERT_4_G Atom Site Occupancy of C214       Constrained at        0.5 Check
PLAT300_ALERT_4_G Atom Site Occupancy of N193       Constrained at     0.3333 Check
PLAT300_ALERT_4_G Atom Site Occupancy of C5AA       Constrained at     0.3333 Check
PLAT300_ALERT_4_G Atom Site Occupancy of C6AA       Constrained at     0.3333 Check
PLAT300_ALERT_4_G Atom Site Occupancy of C54A       Constrained at     0.3333 Check
PLAT300_ALERT_4_G Atom Site Occupancy of C56B       Constrained at     0.3333 Check
PLAT300_ALERT_4_G Atom Site Occupancy of C64A       Constrained at     0.3333 Check
PLAT300_ALERT_4_G Atom Site Occupancy of C85B       Constrained at     0.3333 Check
PLAT300_ALERT_4_G Atom Site Occupancy of C100       Constrained at     0.3333 Check
PLAT300_ALERT_4_G Atom Site Occupancy of H1         Constrained at        0.5 Check
PLAT300_ALERT_4_G Atom Site Occupancy of H4AA       Constrained at        0.5 Check
PLAT300_ALERT_4_G Atom Site Occupancy of H4AB       Constrained at        0.5 Check
PLAT300_ALERT_4_G Atom Site Occupancy of H2A        Constrained at        0.5 Check
PLAT300_ALERT_4_G Atom Site Occupancy of H2B        Constrained at        0.5 Check
PLAT300_ALERT_4_G Atom Site Occupancy of H3         Constrained at        0.5 Check
PLAT300_ALERT_4_G Atom Site Occupancy of H4AC       Constrained at        0.5 Check
PLAT300_ALERT_4_G Atom Site Occupancy of H4         Constrained at        0.5 Check
PLAT300_ALERT_4_G Atom Site Occupancy of H0AA       Constrained at        0.5 Check
PLAT300_ALERT_4_G Atom Site Occupancy of H0AB       Constrained at        0.5 Check
PLAT300_ALERT_4_G Atom Site Occupancy of H0AC       Constrained at        0.5 Check
```

```
PLAT300_ALERT_4_G Atom Site Occupancy of H1AA       Constrained at        0.5 Check
PLAT300_ALERT_4_G Atom Site Occupancy of H1AB       Constrained at        0.5 Check
PLAT300_ALERT_4_G Atom Site Occupancy of H1AC       Constrained at        0.5 Check
PLAT300_ALERT_4_G Atom Site Occupancy of H3AA       Constrained at     0.6667 Check
PLAT300_ALERT_4_G Atom Site Occupancy of H10A       Constrained at        0.5 Check
PLAT300_ALERT_4_G Atom Site Occupancy of H3AB       Constrained at     0.6667 Check
PLAT300_ALERT_4_G Atom Site Occupancy of H11A       Constrained at        0.5 Check
PLAT300_ALERT_4_G Atom Site Occupancy of H11B       Constrained at        0.5 Check
PLAT300_ALERT_4_G Atom Site Occupancy of H11C       Constrained at        0.5 Check
PLAT300_ALERT_4_G Atom Site Occupancy of H3AC       Constrained at     0.6667 Check
PLAT300_ALERT_4_G Atom Site Occupancy of H12A       Constrained at        0.5 Check
PLAT300_ALERT_4_G Atom Site Occupancy of H12B       Constrained at        0.5 Check
PLAT300_ALERT_4_G Atom Site Occupancy of H12C       Constrained at        0.5 Check
PLAT300_ALERT_4_G Atom Site Occupancy of H16A       Constrained at        0.5 Check
PLAT300_ALERT_4_G Atom Site Occupancy of H16B       Constrained at        0.5 Check
PLAT300_ALERT_4_G Atom Site Occupancy of H16C       Constrained at        0.5 Check
PLAT300_ALERT_4_G Atom Site Occupancy of H7AA       Constrained at        0.5 Check
PLAT300_ALERT_4_G Atom Site Occupancy of H19A       Constrained at        0.5 Check
PLAT300_ALERT_4_G Atom Site Occupancy of H19B       Constrained at        0.5 Check
PLAT300_ALERT_4_G Atom Site Occupancy of H19C       Constrained at        0.5 Check
PLAT300_ALERT_4_G Atom Site Occupancy of H7AB       Constrained at        0.5 Check
PLAT300_ALERT_4_G Atom Site Occupancy of H20A       Constrained at        0.5 Check
PLAT300_ALERT_4_G Atom Site Occupancy of H20B       Constrained at        0.5 Check
PLAT300_ALERT_4_G Atom Site Occupancy of H7AC       Constrained at        0.5 Check
PLAT300_ALERT_4_G Atom Site Occupancy of H21A       Constrained at        0.5 Check
PLAT300_ALERT_4_G Atom Site Occupancy of H21B       Constrained at        0.5 Check
PLAT300_ALERT_4_G Atom Site Occupancy of H21C       Constrained at        0.5 Check
PLAT300_ALERT_4_G Atom Site Occupancy of H22A       Constrained at        0.5 Check
PLAT300_ALERT_4_G Atom Site Occupancy of H22B       Constrained at        0.5 Check
PLAT300_ALERT_4_G Atom Site Occupancy of H26A       Constrained at        0.5 Check
PLAT300_ALERT_4_G Atom Site Occupancy of H26B       Constrained at        0.5 Check
PLAT300_ALERT_4_G Atom Site Occupancy of H29A       Constrained at     0.6667 Check
PLAT300_ALERT_4_G Atom Site Occupancy of H29B       Constrained at     0.6667 Check
PLAT300_ALERT_4_G Atom Site Occupancy of H54B       Constrained at     0.6667 Check
PLAT300_ALERT_4_G Atom Site Occupancy of H61A       Constrained at        0.5 Check
PLAT300_ALERT_4_G Atom Site Occupancy of H61B       Constrained at        0.5 Check
PLAT300_ALERT_4_G Atom Site Occupancy of H61C       Constrained at        0.5 Check
PLAT300_ALERT_4_G Atom Site Occupancy of H61D       Constrained at        0.5 Check
PLAT300_ALERT_4_G Atom Site Occupancy of H61E       Constrained at        0.5 Check
PLAT300_ALERT_4_G Atom Site Occupancy of H67A       Constrained at        0.5 Check
PLAT300_ALERT_4_G Atom Site Occupancy of H70A       Constrained at        0.5 Check
PLAT300_ALERT_4_G Atom Site Occupancy of H83A       Constrained at     0.6667 Check
PLAT300_ALERT_4_G Atom Site Occupancy of H83B       Constrained at     0.6667 Check
PLAT300_ALERT_4_G Atom Site Occupancy of H83C       Constrained at     0.6667 Check
PLAT300_ALERT_4_G Atom Site Occupancy of H85A       Constrained at     0.6667 Check
PLAT300_ALERT_4_G Atom Site Occupancy of H85C       Constrained at        0.5 Check
PLAT300_ALERT_4_G Atom Site Occupancy of H85D       Constrained at        0.5 Check
PLAT300_ALERT_4_G Atom Site Occupancy of H85E       Constrained at        0.5 Check
PLAT300_ALERT_4_G Atom Site Occupancy of H86A       Constrained at        0.5 Check
PLAT300_ALERT_4_G Atom Site Occupancy of H86B       Constrained at        0.5 Check
PLAT300_ALERT_4_G Atom Site Occupancy of H86C       Constrained at        0.5 Check
PLAT300_ALERT_4_G Atom Site Occupancy of H87A       Constrained at     0.6667 Check
PLAT300_ALERT_4_G Atom Site Occupancy of H87B       Constrained at     0.6667 Check
PLAT300_ALERT_4_G Atom Site Occupancy of H87C       Constrained at     0.6667 Check
PLAT300_ALERT_4_G Atom Site Occupancy of H89        Constrained at     0.6667 Check
PLAT300_ALERT_4_G Atom Site Occupancy of H92A       Constrained at        0.5 Check
PLAT300_ALERT_4_G Atom Site Occupancy of H92B       Constrained at        0.5 Check
```

```
PLAT300_ALERT_4_G Atom Site Occupancy of H93A       Constrained at       0.5 Check
PLAT300_ALERT_4_G Atom Site Occupancy of H93B       Constrained at       0.5 Check
PLAT300_ALERT_4_G Atom Site Occupancy of H10B       Constrained at    0.3333 Check
PLAT300_ALERT_4_G Atom Site Occupancy of H10C       Constrained at    0.3333 Check
PLAT300_ALERT_4_G Atom Site Occupancy of H10D       Constrained at    0.3333 Check
PLAT300_ALERT_4_G Atom Site Occupancy of H5AA       Constrained at    0.3333 Check
PLAT300_ALERT_4_G Atom Site Occupancy of H5AB       Constrained at    0.3333 Check
PLAT300_ALERT_4_G Atom Site Occupancy of H5AC       Constrained at    0.3333 Check
PLAT300_ALERT_4_G Atom Site Occupancy of H6AA       Constrained at    0.3333 Check
PLAT300_ALERT_4_G Atom Site Occupancy of H6AB       Constrained at    0.3333 Check
PLAT300_ALERT_4_G Atom Site Occupancy of H6AC       Constrained at    0.3333 Check
PLAT300_ALERT_4_G Atom Site Occupancy of H19D       Constrained at    0.3333 Check
PLAT300_ALERT_4_G Atom Site Occupancy of H19E       Constrained at    0.3333 Check
PLAT300_ALERT_4_G Atom Site Occupancy of H54A       Constrained at    0.3333 Check
PLAT300_ALERT_4_G Atom Site Occupancy of H64A       Constrained at    0.3333 Check
PLAT300_ALERT_4_G Atom Site Occupancy of H85B       Constrained at    0.3333 Check
PLAT301_ALERT_3_G Main Residue  Disorder .............(Resd  1  )        29% Note
PLAT367_ALERT_2_G Long?  C(sp?)-C(sp?) Bond  C79      - C84      .      1.62 Ang.
PLAT410_ALERT_2_G Short Intra H...H Contact  H4       ..H47      .      2.09 Ang.
                                                    x,y,z  =     1_555 Check
PLAT410_ALERT_2_G Short Intra H...H Contact  H41      ..H61A     .      2.01 Ang.
                                                    x,y,z  =     1_555 Check
PLAT410_ALERT_2_G Short Intra H...H Contact  H41      ..H61B     .      2.13 Ang.
                                                    x,y,z  =     1_555 Check
PLAT410_ALERT_2_G Short Intra H...H Contact  H42      ..H93B     .      2.03 Ang.
                                                    x,y,z  =     1_555 Check
PLAT410_ALERT_2_G Short Intra H...H Contact  H47      ..H54A     .      2.11 Ang.
                                                    x,y,z  =     1_555 Check
PLAT412_ALERT_2_G Short Intra XH3 .. XHn     H4AA     ..H58      .      2.09 Ang.
                                                    x,y,z  =     1_555 Check
PLAT412_ALERT_2_G Short Intra XH3 .. XHn     H0AC     ..H52      .      1.92 Ang.
                                                    x,y,z  =     1_555 Check
PLAT412_ALERT_2_G Short Intra XH3 .. XHn     H1AB     ..H59      .      1.89 Ang.
                                                   -y,x,z  =     2_555 Check
PLAT412_ALERT_2_G Short Intra XH3 .. XHn     H11A     ..H46      .      1.88 Ang.
                                                    x,y,z  =     1_555 Check
PLAT412_ALERT_2_G Short Intra XH3 .. XHn     H50      ..H10D     .      2.13 Ang.
                                                    x,y,z  =     1_555 Check
PLAT412_ALERT_2_G Short Intra XH3 .. XHn     H52      ..H85C     .      2.13 Ang.
                                                    x,y,z  =     1_555 Check
PLAT606_ALERT_4_G Solvent Accessible VOID(S) in Structure ........         ! Info
PLAT720_ALERT_4_G Number of Unusual/Non-Standard Labels ..........        32 Note
PLAT764_ALERT_4_G Overcomplete CIF Bond List Detected (Rep/Expd) .      1.18 Ratio
PLAT793_ALERT_4_G Model has Chirality at S5         (Centro SPGR)          R Verify
PLAT793_ALERT_4_G Model has Chirality at S6         (Centro SPGR)          S Verify
PLAT793_ALERT_4_G Model has Chirality at S7         (Centro SPGR)          R Verify
PLAT794_ALERT_5_G Tentative Bond Valency for Mn2       (II)      .      2.23 Info
PLAT794_ALERT_5_G Tentative Bond Valency for Mn3       (II)      .      2.23 Info
PLAT811_ALERT_5_G No ADDSYM Analysis: Too Many Excluded Atoms ....         ! Info
PLAT860_ALERT_3_G Number of Least-Squares Restraints ............        791 Note
```

---

0 **ALERT level A** = Most likely a serious problem - resolve or explain
4 **ALERT level B** = A potentially serious problem, consider carefully
21 **ALERT level C** = Check. Ensure it is not caused by an omission or oversight
165 **ALERT level G** = General information/check it is not something unexpected

```
  3 ALERT type 1 CIF construction/syntax error, inconsistent or missing data
 32 ALERT type 2 Indicator that the structure model may be wrong or deficient
  9 ALERT type 3 Indicator that the structure quality may be low
142 ALERT type 4 Improvement, methodology, query or suggestion
  4 ALERT type 5 Informative message, check
```

It is advisable to attempt to resolve as many as possible of the alerts in all categories. Often the minor alerts point to easily fixed oversights, errors and omissions in your CIF or refinement strategy, so attention to these fine details can be worthwhile. In order to resolve some of the more serious problems it may be necessary to carry out additional measurements or structure refinements. However, the purpose of your study may justify the reported deviations and the more serious of these should normally be commented upon in the discussion or experimental section of a paper or in the "special_details" fields of the CIF. checkCIF was carefully designed to identify outliers and unusual parameters, but every test has its limitations and alerts that are not important in a particular case may appear. Conversely, the absence of alerts does not guarantee there are no aspects of the results needing attention. It is up to the individual to critically assess their own results and, if necessary, seek expert advice.

Publication of your CIF in IUCr journals

A basic structural check has been run on your CIF. These basic checks will be run on all CIFs submitted for publication in IUCr journals (*Acta Crystallographica*, *Journal of Applied Crystallography*, *Journal of Synchrotron Radiation*); however, if you intend to submit to *Acta Crystallographica Section C* or *E* or *IUCrData*, you should make sure that full publication checks are run on the final version of your CIF prior to submission.

Publication of your CIF in other journals

Please refer to the *Notes for Authors* of the relevant journal for any special instructions relating to CIF submission.

PLATON version of 12/09/2022; check.def file version of 09/08/2022

Datablock mn24 - ellipsoid plot

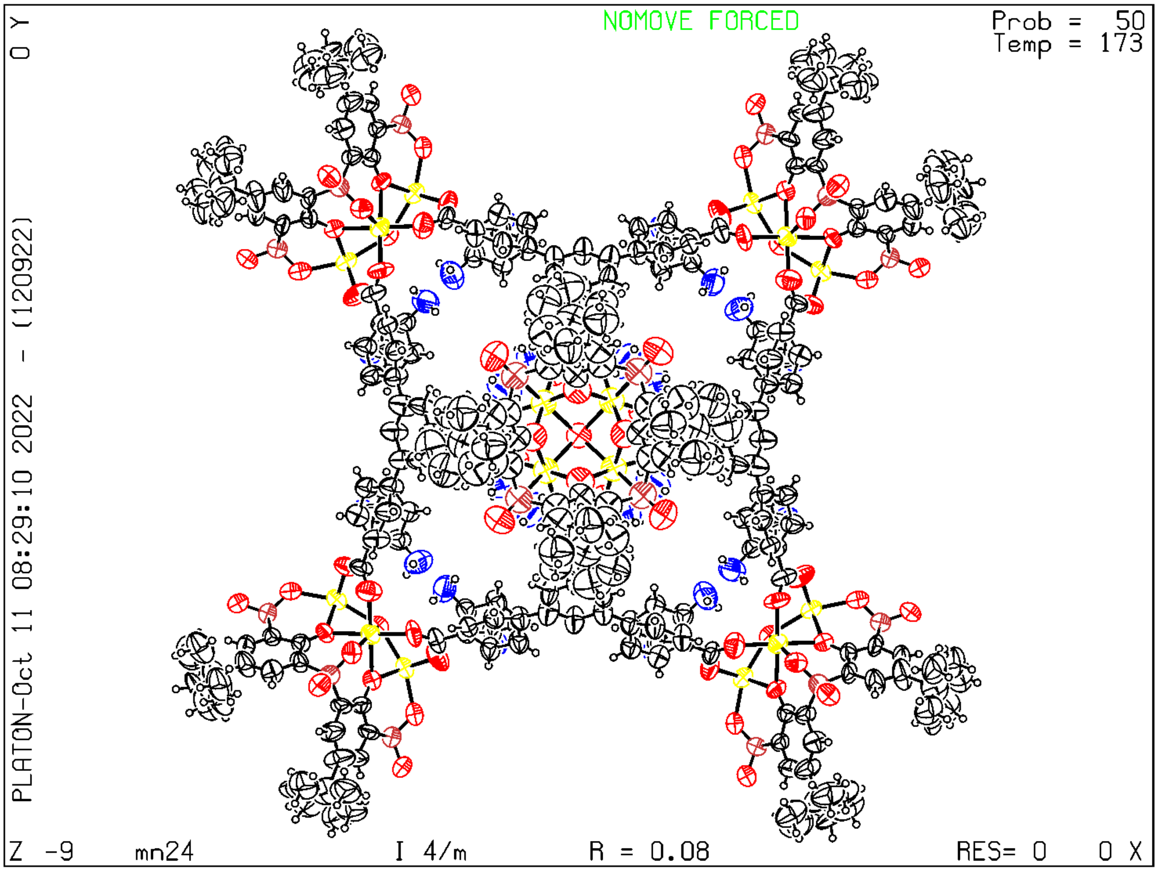

Supplement: Supplementary file 15 — oc3c00027_si_015.pdf [file oc3c00027_si_015.pdf]
